# DiICz MR-TADF emitters as potent energy transfer photocatalysts

**DOI:** 10.1039/d5sc04014k

**Published:** 2025-10-28

**Authors:** Lea Hämmerling, David Hall, Eliott Blin, Tabea Heil, Eli Zysman-Colman

**Affiliations:** a Organic Semiconductor Centre, EaStCHEM School of Chemistry, University of St Andrews St Andrews KY16 9ST UK eli.zysman-colman@st-andrews.ac.uk

## Abstract

Photoinduced energy transfer (PEnT) reactions are a subset of photochemical reactions that involve the indirect photoactivation of substrates following an energy transfer from a photoexcited sensitizer/photocatalyst. Examples of PEnT reactions include *E*/*Z* isomerizations, [2 + 2] cycloadditions, and sigmatropic shifts. Here we introduce a family of diindolocarbazole (DiICz) multi-resonant thermally activated delayed fluorescent (MR-TADF) photocatalysts (PCs), DiICztBu_4_, DiICzMes_4_, DiICztBuCz_4_, and DiICztBuDPA_4_, which have relatively high triplet energies (*E*_T_). We cross-compare their photocatalytic behavior and that of relevant literature reference PCs' in five distinct PEnT reactions. We demonstrate that the use of the DiICz PCs consistently leads to more rapid reaction rates and higher yields compared to the widely used 4CzIPN. DiICztBu_4_, DiICzMes_4_, and DiICztBuCz_4_ possess similar *E*_T_ but decreasing singlet–triplet energy gaps, Δ*E*_ST_, enabling for the first time a comparison of the dependency of both the reaction kinetics and the final yield on this photophysical parameter. We observed that when the reaction kinetics are fast, there is little sensitivity to quenching of the excited PC by oxygen, implying that Dexter Energy Transfer (DET) to the substrate is competitive with DET to oxygen. Importantly, this means that some of the DET reactions using these PCs can be performed in air without adversely affecting reaction yield.

## Abstract 2

Here we introduce a family of diindolocarbazole (DiICz) multi-resonant thermally activated delayed fluorescent (MR-TADF) compounds DiICztBu_4_, DiICzMes_4_, DiICztBuCz_4_, and DiICztBuDPA_4_, as potent energy transfer photocatalysts (PCs).

## Graphical abstract

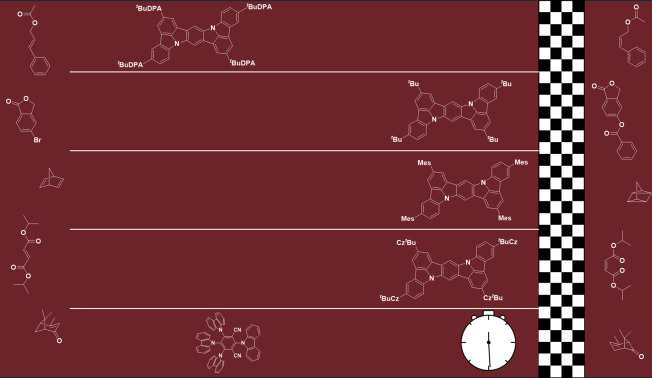

## Introduction

One major class of photochemical reactions consists of those that proceed *via* a photoinduced energy transfer (PEnT) mechanism, which typically involves an electronically excited photocatalyst (PC*) transferring its energy to a substrate (sub). The substrate in its electronically excited state (sub*) then undergoes a photochemical transformation.^1–3^ The majority of PEnT reactions proceed *via* Dexter Energy Transfer (DET) reactions. DET involves the simultaneous double electron exchange between the PC* and the sub to generate a PC in its S_0_ state and a sub*. This can take place from the singlet state of the ^1^PC* to generate ^1^sub* or more commonly from the triplet state of ^3^PC* to generate ^3^sub* (Fig. S1) and does not involve a change in the overall multiplicity.^1^ DET processes occur over shorter distances, typically less than 10 Å,^4^ as orbital overlap between the PC and the sub is required, which implies a collisional interaction is needed for DET to occur intermolecularly.

In cases where DET occurs from the ^3^PC* to ^3^sub, it is the spectral overlap of the phosphorescence of the ^3^PC* and the spin-forbidden absorption of the substrate that is relevant to generate ^3^sub*. Notably, as the spin-forbidden absorption spectrum is of negligible intensity in most organic substrates, the low-energy onset of the low-temperature phosphorescence spectrum of the sub is used as a surrogate estimation of the energy at which spectral overlap no longer occurs, which is none other than the energy of the T_1_ state (*E*_T_) of the sub. It is generally accepted that DET will occur when *E*_T_(PC*) > *E*_T_(sub*), and the closer these two values are, the more likely DET is to occur.^5^ Given that the ^3^sub* has a biradical-like character, this enables a diversity of reactions such as *E*/*Z* isomerization,^2,6–8^ cycloadditions such as [2 + 2],^9–11^ sensitization of metal complexes,^12–14^ homolytic bond cleavages such as N_2_ release from benzoyl azides, scission of S–S bonds, and N–O dissociation of oxime esters to instigate the formation of carbon-centered and nitrogen-centered radicals in concert with the loss of CO_2_.^15–17^

**Fig. 1:**
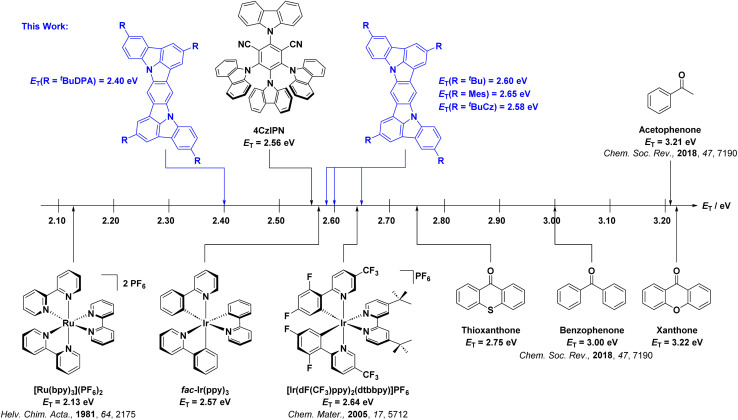
Chemical structures and *E*_T_ for selected literature PCs used for DET reactions and for the four TADF PCs investigated in this work. Quoted *E*_T_ values for 4CzIPN, DiICztBu_4_, DiICzMes_4_, DiICztBuCz_4_ and DiICztBuDPA_4_ were estimated from *E*_T_ = *E*_S_ − Δ*E*_ST_, with *E*_S_ being the onset of the steady-state emission in DCM at room temperature and Δ*E*_ST_ determined in 2-MeTHF glass at 77 K. *E*_T_ for *fac*-Ir(ppy)_3_ was taken from the onset of the room temperature emission in 2-MeTHF. *E*_T_ for [Ru(bpy)_3_](PF_6_)_2_ was taken from the emission maximum at 77 K in 4 : 1 MeOH/EtOH glass.^22^*E*_T_ for [Ir(dF(CF_3_)ppy)_2_(dtbbpy)]PF_6_ was taken from the room temperature emission maximum in MeCN.^23^ For the organic PCs, *E*_T_ is measured from the phosphorescence spectra at 77 K.^5^

Of the PCs typically employed in PEnT reactions, organic carbonyl-based photosensitizers have the highest *E*_T_. For instance, xanthone and acetophenone (Fig. 1) have similar *E*_T_ values of 3.22 eV (74.2 kcal mol^−1^, 310.5 kJ mol^−1^) and 3.21 eV (74.0 kcal mol^−1^, 309.6 kJ mol^−1^).^1,2,18^ Organometallic PCs are particularly popular for PEnT reactions in part because the presence of the heavy metal center ensures an essentially quantitative triplet yield as a result of fast intersystem crossing (ISC) mediated by its large spin–orbit coupling (SOC).^19,20^ Two of the most commonly used organometallic PCs for PEnT reactions include [Ir(dF(CF_3_)ppy)_2_(dtbbpy)](PF_6_) (*E*_T_ = 2.68 eV)^5^ for reactions requiring a relatively high *E*_T_ and [Ru(bpy)_3_](PF_6_)_2_ (*E*_T_ = 2.12 eV)^21^ for those that do not (Fig. 1).

Over the last decade or so, organic thermally activated delayed fluorescence (TADF) compounds have been increasingly used in the academic community as alternatives to organometallic complexes, initially as emissive materials for organic light-emitting diodes (OLEDs),^24^ and later, as PCs.^25,26^ This is because TADF compounds have accessible triplet states owing to the small energy gap (Δ*E*_ST_) between their lowest excited singlet (S_1_) and triplet (T_1_) states, which allows for relatively fast ISC and reverse ISC (RISC) to take place, despite the small SOC between these states in the absence of heavy atoms.^27,28^ Molecules with small Δ*E*_ST_ are those where the donor (D) and acceptor (A) moieties are sufficiently electronically decoupled such that there is minimal orbital overlap between the highest occupied molecular orbital (HOMO) and the lowest unoccupied molecular orbital (LUMO), *i.e.*, the exchange energy is small. This spatial separation also produces an emissive excited state having charge transfer (CT) character. The most widely employed molecular design is one that adopts a strongly twisted D–A structure, exemplified in 4CzIPN and 4DPAIPN (Fig. S2).^26^ Owing to the distance between D and A units, the emissive S_1_ state possesses long-range charge transfer (LRCT) character.^29^ D–A TADF PCs based on carbazoyl dicyanobenzenes (CDCBs) have been extensively employed in myriad photocatalytic reactions. Examples from this family of TADF PCs cover a broad range of *E*_T_. For example, the *E*_T_ values of 4DPAIPN, 4CzTPN, 2CzIPN, 4CzIPN, and 3,5-2CzBN increase from 2.30 eV ^30^ to 2.34,^31^ 2.72,^31^ 2.73,^32^ and 3.03 eV,^31^ respectively. These values overlap with those of, for example, [Ir(dF(CF_3_)ppy)_2_(dtbbpy)]PF_6_ (*E*_T_ = 2.64 eV ^23^) and benzophenone (*E*_T_ = 3.00 eV ^33^). Beyond CDCBs, other D–A TADF PEnT PCs include pDTCz-DPmS^34^ and DI-PF^35^ (Fig. S2). pDTCz-DPmS (*E*_T_ = 2.93 eV) possesses a suitable *E*_T_ to photocatalyze the *E*/*Z* isomerization of diisopropyl fumarate (*E*_T_ = 2.7 eV),^36^ producing 81% of diisopropyl maleate while 4CzIPN only yields 6%, owing to its lower *E*_T_ of 2.68 eV.^37^DI-PF (*E*_T_ = 2.38 eV) yielded 66% *Z*-stilbene in the *E*/*Z* isomerization of *E*-stilbene (*E*_T_ = 2.2 eV) despite its lower triplet energy, while 4CzIPN affords 87% of the desired product.^35^ Another class of TADF PCs contains an imidazo-phenothiazine (IPTZ) acceptor unit connected with different donor groups. The PCs ACR-IMAC, ACR-IPTZ, and SACR-IPTZ have similar triplet energies of *E*_T_ = 2.76 eV,^38^ 2.76 eV,^39^ and 2.77 eV,^39^ respectively. Their use in a range of reactions, such as the [2 + 2] cycloaddition of an indole and dimethylphenylvinylsilane, afforded near quantitative product yields.^38,39^ A second class of TADF compounds is the so-called multi-resonant TADF (MR-TADF) emitters. These are rigid polycyclic aromatic hydrocarbons that are typically doped with both electron-rich and electron-deficient groups. Suitable regiochemistry of these p- and n-dopants produces an alternating pattern of increasing and decreasing electron density in the excited state as compared to the ground state, leading to the necessary small exchange energy that turns on TADF. Given the short distance between D and A motifs, the emissive excited state is of short-range CT (SRCT) character.^40^ Compared to D–A TADF compounds, MR-TADF emitters have more intense low energy absorption bands, narrower emission spectra, smaller Stokes shifts, and their emission spectra show only minimal positive solvatochromism—all potentially attractive properties for their use as PCs.^40^ Our group has demonstrated the broad utility of two families of MR-TADF PCs in a range of photoinduced electron transfer (PET) and PEnT reactions.^41,42^ These two families contain nitrogen donor atoms and either boron or carbonyl groups as n-dopants within the polycyclic aromatic hydrocarbon framework.

**Fig. 2:**
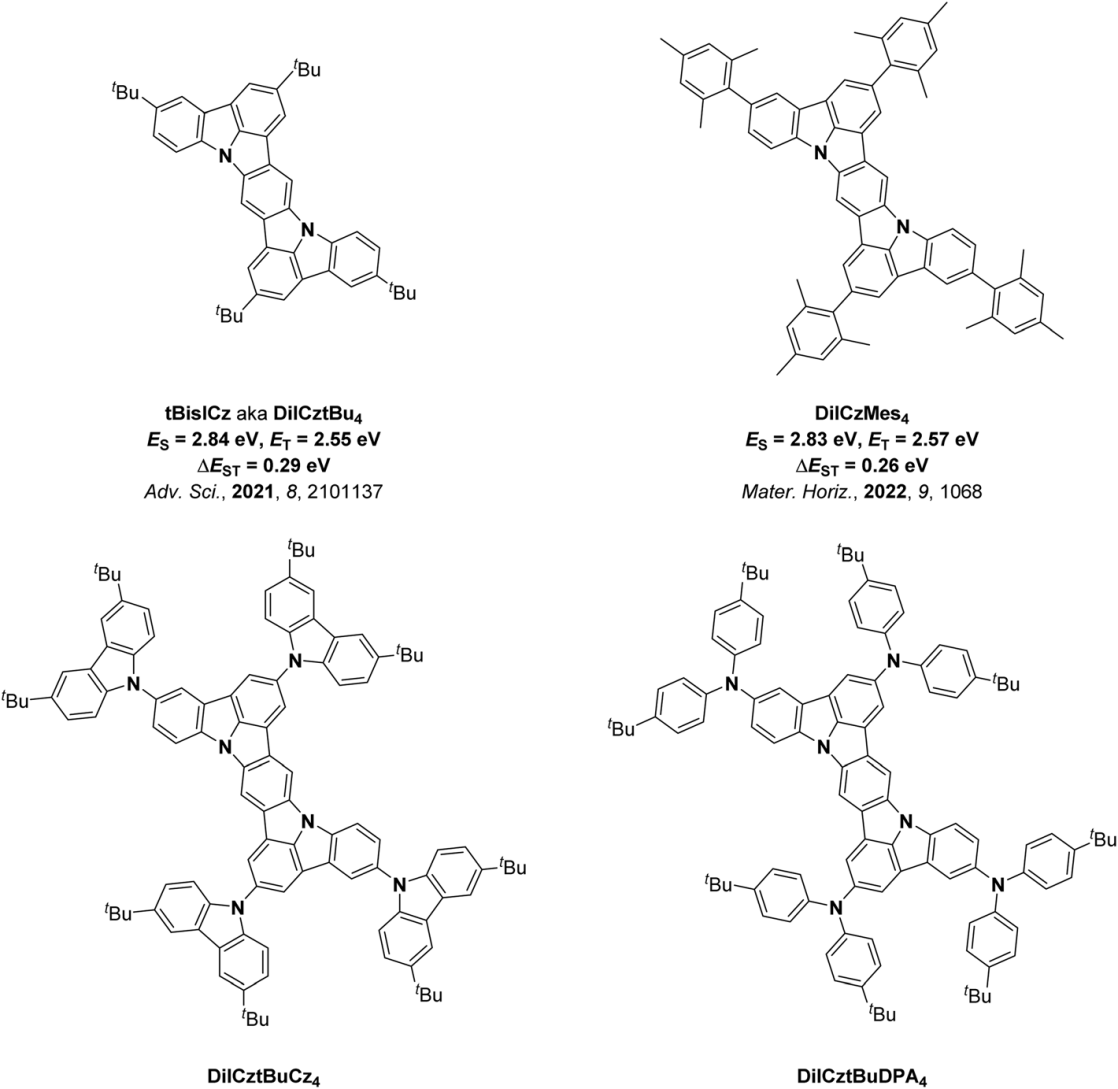
Chemical structures of DiICztBu_4_, DiICzMes_4_, DiICztBuCz_4_, and DiICztBuDPA_4_.

We, as well as Lee and co-workers, recently showed that the acceptor moiety is not required in MR-TADF emitter design for OLEDs.^43–47^ Indeed, when the nitrogen atoms in diindolocarbazoles are *para*-disposed, there is very weak TADF as Δ*E*_ST_ is moderately large, resulting in slow *k*_RISC_. DiICztBu_4_ and DiICzMes_4_ have similar *E*_T_ of 2.55 (in DCM)^43^ and 2.57 eV (in toluene),^44^ respectively, while their reported Δ*E*_ST_ values are 0.29 and 0.26 eV. Recognizing their potential as PCs, herein we investigated a series of four structurally related DiICz-based TADF PCs, DiICztBu_4_, DiICzMes_4_, DiICztBuCz_4_, and DiICztBuDPA_4_, in DET reactions (Fig. 2); the first two have previously been reported, while DiICztBuCz_4_ and DiICztBuDPA_4_ were, coincident with this work, recently reported by Lee and co-workers.^48^ Across the first three compounds in this series, there is a progressive stabilization of the S_1_ state, while the T_1_ energy is largely unaffected; both S_1_ and T_1_ are stabilized in DiICztBuDPA_4_. Thus, the impact of Δ*E*_ST_ on the performance in DET is probed for the first time. While we did not observe a dependency of the efficiency of the PEnT reactions as a function of the Δ*E*_ST_ of the PC across five different PEnT reactions, four involving direct DET to the substrate and one nickel-sensitized dual photocatalysis reaction, we did discover that these PCs initiate fast PEnT reactions that show remarkably little sensitivity to the presence of O_2_, despite it being a competitive triplet quencher. In the four PEnT reactions with direct energy transfer to the substrate, DiICztBu_4_, DiICzMes_4_, and DiICztBuCz_4_ consistently outperform 4CzIPN both in terms of NMR yield and reaction rate.

## Results and discussion

**Fig. 3:**
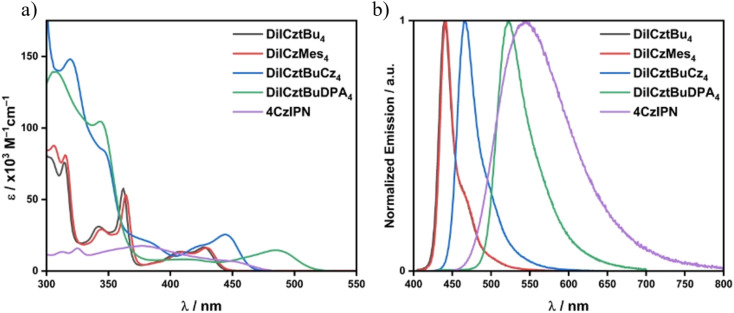
(a) Absorption and (b) steady-state PL spectra in DCM of DiICztBu_4_ (*λ*_exc_ = 340 nm), DiICzMes_4_ (*λ*_exc_ = 380 nm), DiICztBuCz_4_ (*λ*_exc_ = 340 nm), DiICztBuDPA_4_ (*λ*_exc_ = 400 nm), and 4CzIPN (*λ*_exc_ = 378 nm).

The syntheses of DiICztBu_4_, DiICzMes_4_, DiICztBuCz_4_, and DiICztBuDPA_4_ are detailed in the SI. The UV-vis absorption spectra of these compounds in DCM, along with that of the reference PC 4CzIPN, are shown in Fig. 3a. DiICztBu_4_, DiICzMes_4_, DiICztBuCz_4_, and DiICztBuDPA_4_ all exhibit low-energy absorption bands in the visible region that are more intense than that of 4CzIPN. The presence of the mesityl (Mes) groups in DiICzMes_4_ leads to a very slight red shift in the absorption spectrum compared to DiICztBu_4_, while the substitution of the diindolocarbazole with progressively stronger carbazole (Cz) and diarylamine donors results in a larger red shift of these bands (Fig. 3a and S37). A similar trend is observed in their photoluminescence (PL) spectra (Fig. 3b), where the PL maximum in DCM shifts from *λ*_PL_ of 440 to 442, 466, and 532 nm for DiICztBu_4_, DiICzMes_4_, DiICztBuCz_4_, and DiICztBuDPA_4_, respectively (Fig. 3b and Table 2). Their emission is more narrowband compared to the broad PL of 4CzIPN, reflecting their more rigid structures.

The S_1_ and T_1_ energies were determined from the onsets of the steady-state PL and delayed emission (gate time: 1–9 ms) spectra in 2-MeTHF glass at 77 K, respectively (Fig. S40); the Δ*E*_ST_ value is the difference in energy between these two (Fig. S40 and Table 1). DiICztBu_4_, DiICzMes_4_, and DiICztBuCz_4_ have very similar *E*_T_ of 2.58, 2.57, and 2.55 eV, respectively. As their emission profiles are similar, so too will be their spectral overlap with the sub, and thus the *k*_DET_ should be comparable across these three PCs. The S_1_ energy, *E*_S_, however, progressively decreases from 2.91 to 2.84 and 2.77 eV (measured at 77 K in 2-MeTHF); thus, the Δ*E*_ST_ likewise narrows from 0.33 to 0.27 and 0.22 eV. The *E*_S_ and *E*_T_ for DiICztBuDPA_4_ are 2.54 and 2.41 eV, resulting in the smallest Δ*E*_ST_ of 0.13 eV. The *E*_T_ values determined at cryogenic temperatures of these four derivatives are all lower than the 2.73 eV of 4CzIPN (Fig. S40e). DiICztBu_4_ and DiICzMes_4_ have previously been reported as MR-TADF emitters, and given the similar photophysical properties of DiICztBuCz_4_ and DiICztBuDPA_4_ to these two, the high molar absorptivity of the low-energy absorption band, the minimal positive PL solvatochromism, the narrowband emission and the small Δ*E*_ST_, they can also be classified as MR-TADF (Table 2).

**Table 1:** Excited-state energies for DiICztBu_4_, DiICzMes_4_, DiICztBuCz_4_, DiICztBuDPA_4_, and 4CzIPN

Compound	77 K values^a^			Room temperature *E*_T_^b^/eV			
	*E*_S_/eV	*E*_T_/eV	Δ*E*_ST_/eV	Toluene	EtOAc	DCM	DMF
DiICztBu_4_	2.91	2.58	0.33	2.60	2.63	2.60	2.61
DiICzMes_4_	2.84	2.57	0.27	2.64	2.66	2.65	2.65
DiICztBuCz_4_	2.77	2.55	0.22	2.58	2.61	2.58	2.58
DiICztBuDPA_4_	2.54	2.41	0.13	2.44	2.45	2.40	2.46
4CzIPN	2.77	2.73	0.04	2.67	2.66	2.56	2.54

a 77 K values were measured in 2-MeTHF glass. *E*_S_ was determined from the onset of the steady-state PL and *E*_T_ from the onset of the gated emission acquired in a time window of 1–9 ms after excitation. Δ*E*_ST_ = *E*_S_ − *E*_T_. *λ*_exc_ = 390 nm for DiICztBu_4_, DiICzMes_4_, and DiICztBuCz; *λ*_exc_ = 400 nm for DiICztBuDPA_4_ and *λ*_exc_ = 380 nm for 4CzIPN.

b Room temperature *E*_T_ values were estimated following our previously reported methodology,^49^ where *E*_T_(RT) = *E*_S_(RT) − Δ*E*_ST_(LT), with *E*_S_(RT) being the onset of the steady-state PL in the respective solvents and Δ*E*_ST_(LT) being measured in 2-MeTHF at 77 K.

**Table 2:** Solution-state photophysical properties of DiICztBu_4_, DiICzMes_4_, DiICztBuCz_4_, and DiICztBuDPA_4_ and the reference photocatalysts 4CzIPN and *fac*-Ir(ppy)_3_

Compound	*λ*_abs_ a/nm (*ε*/10^3^ M^−1^ cm^−1^)	*λ*_PL_ a/nm	*E*_S_ b/eV	*E*_T_ b/eV	Δ*E*_ST_	*τ*_PL_ c/ns
DiICztBu_4_	305 (58), 315 (67), 343 (29), 362 (54), 408 (13), 427 (16)	440	2.91	2.58	0.33	9 (8)
DiICzMes_4_	300 (84), 306 (88), 315 (81), 344 (29), 364 (53), 409 (13), 430 (16)	442	2.84	2.57	0.27	10 (8)
DiICztBuCz_4_	319 (152), 347 s (84), 385 s (20), 420 s (17), 444 (25)	466	2.77	2.55	0.22	10 (8)
DiICztBuDPA_4_	305 (141), 343 (104), 411 (7), 485 (14)	523	2.54	2.41	0.13	16 (13)
4CzIPN	313 (14), 325 (16), 377 (18), 450 s (7)	544	2.77	2.73	0.04	1896 (218)^d^
*fac*-Ir(ppy)_3_	344 (10), 378 (13), 409 (8), 455 (3)	520	—	2.58	—	1391 (54)

a In DCM at 77 K, taken from the onset of steady-state PL spectrum for *E*_S_ and the onset of the gated emission spectrum (1–9 ms) for *E*_T_.

b In 2-MeTHF at 77 K, taken from the onset of steady-state PL spectrum for *E*_S_ and the onset of the gated emission spectrum (1–9 ms) for *E*_T_.

c Under degassed conditions, excited-state lifetimes in air in parentheses.

d Lifetime is the average lifetime of 4CzIPN calculated as *τ*_avg_ = *τ*_1_ × *w*_1_ + *τ*_2_ × *w*_2_.

While the low temperature (LT) measurements, at 77 K, permit a robust estimation of Δ*E*_ST_, noting that in the vast majority of TADF compounds the S_1_ state has greater CT character than T_1_, this measurement will not accurately capture *E*_S_ at room temperature (RT) in solution. This is because CT states are stabilized as a function of solvent polarity, and the greater the CT character, the stronger the stabilization of the state.^50^ As triplet states tend to show lesser CT character than singlets, the estimated *E*_T_ = *E*_S_(RT) − Δ*E*_ST_(LT) represents the outer bound value for what *E*_T_ may be at room temperature. Thus, given the LRCT character of the S_1_ state of 4CzIPN, the change in *E*_T_ at room temperature is more pronounced than for the four DiICz MR-TADF compounds (Table 1). This implies that in DET reactions, the spectral overlap between the PC* and the sub may change significantly, especially when the T_1_ state possesses some CT character. For MR-TADF compounds, this stabilization effect is small given the SRCT character of the S_1_ and T_1_ states. So, at low temperature, the *E*_T_ values are 2.58, 2.57, 2.55, and 2.41 eV in 2-MeTHF for DiICztBu_4_, DiICzMes_4_, DiICztBuCz_4_, and DiICztBuDPA_4_, respectively, while in DCM at room temperature, the *E*_T_ values remain essentially the same at 2.60, 2.65, 2.58, and 2.40 eV, respectively (Table 1). This contrasts with the effect on the T_1_ energy of 4CzIPN (*E*_T_(LT) = 2.73 eV *vs. E*_T_(RT) = 2.56 eV; where LT is low temperature, 77 K, and RT is room temperature). This is illustrated in a comparison between the T_1_ energies in 2-MeTHF glass and DCM solutions, Δ*E*_T–T_, which is 0.08 eV for DiICzMes_4_ and 0.17 eV for 4CzIPN. Thus, these four MR-TADF compounds maintain their *E*_T_ in polar solvents, while for 4CzIPN, the *E*_T_ is much more stabilized. The time-resolved PL decays of DiICztBu_4_, DiICzMes_4_, DiICztBuCz_4_, and DiICztBuDPA_4_ in DCM are shown in Fig. S40 and S41 and are listed in Table 2. There is no delayed emission, and PL lifetimes range from 9–16 ns. Given the moderately large Δ*E*_ST_, it is not uncommon for MR-TADF emitters to not show delayed emission in solution as non-radiative decay competes with RISC, while in the solid state, this is largely suppressed, and delayed emission becomes apparent.

## DET reactions

We began by studying intramolecular DET reactions with photocatalysts of increasing triplet energy to focus on the interaction between the PC and a single molecule. We first investigated the *E*/*Z* isomerization of an alkene, *E*-cinnamyl acetate, with a T_1_ energy similar to those of the MR-TADF emitters; notably, the structurally similar *E*-methyl cinnamate has a *E*_T_ = 2.38 eV ^51^ (Table S7). We expect the triplet energy of *E*-cinnamyl acetate, which contains a methylene group between the styrenyl moiety and the ester group, to be slightly higher compared to *E*-methyl cinnamate, which has a larger conjugation length. The geometric isomerization does not take place in the absence of an irradiated PC (Table 3, entries 1 and 2). After 24 h of irradiation under N_2_, the use of DiICztBu_4_, DiICzMes_4_, and DiICztBuCz_4_ as PCs yielded comparable *E*/*Z* ratios of 18/82, 19/81, and 19/81, respectively (Table 3, entries 4, 6, and 8), performing as well in this reaction as 4CzIPN (*E*/*Z* ratio of 14/86, Table 3, entry 12). Similar *E*/*Z* ratios are observed under air (*E*/*Z* = 19/81, 17/84, 20/80, and 15/85 for DiICztBu_4_, DiICzMes_4_, DiICztBuCz_4_, and 4CzIPN, respectively, Table 3, entries 3, 5, 7, and 11). Amazingly, the end ratio obtained for this DET reaction is unaffected despite O_2_ being a competitive triplet quencher. This is, however, not the case with DiICztBuDPA_4_, which under N_2_ yielded an *E*/*Z* ratio of 28/72, a lower ratio than those obtained with the other PCs, while there is effectively no isomerization observed in air (Table 3, entries 9 and 10).

**Table 3:** *E*/*Z* isomerization of cinnamyl acetate^a^

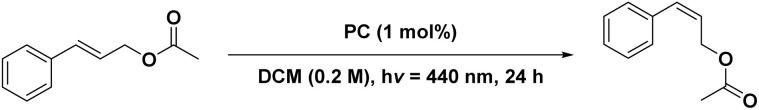			
Entry	Photocatalyst	Conditions	*E*/*Z* ratio
1	None	Air	99/1 ± 0
2	None	N_2_	99/1 ± 0
3	DiICztBu_4_	Air	19/81 ± 0
4	DiICztBu_4_	N_2_	18/82 ± 0
5	DiICzMes_4_	Air	16/84 ± 1
6	DiICzMes_4_	N_2_	19/81 ± 0
7	DiICztBuCz_4_	Air	20/80 ± 0
8	DiICztBuCz_4_	N_2_	19/81 ± 0
9	DiICztBuDPA_4_	Air	98/2 ± 0
10	DiICztBuDPA_4_	N_2_	28/72 ± 0
11	4CzIPN	Air	15/85 ± 0
12	4CzIPN	N_2_	14/86 ± 0

a Cinnamyl acetate (0.2 mmol) in DCM (0.2 M) with 1 mol% PC loading. *E*/*Z* ratios were determined *via*^1^H NMR spectroscopy. Reported yields are the mean from at least two reactions with associated standard deviations.

**Fig. 4:**
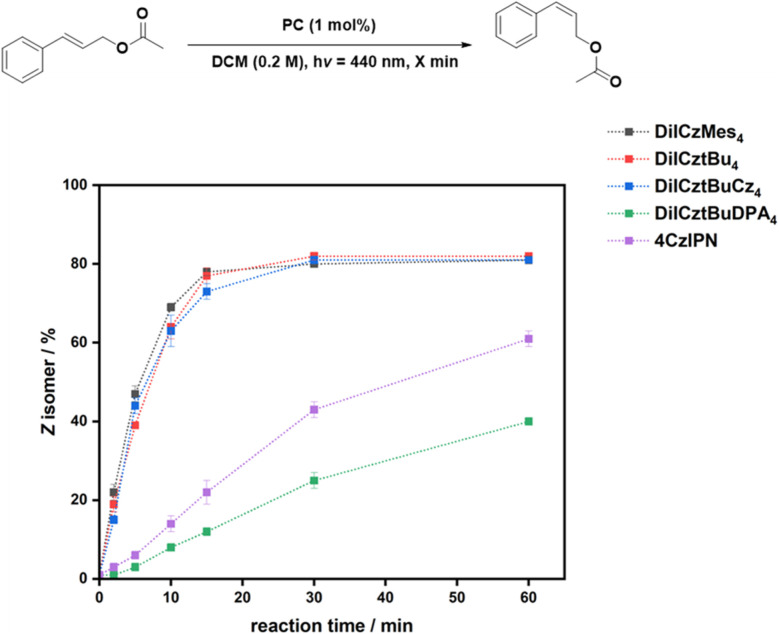
Reaction progression for the *E*/*Z* isomerization of cinnamyl acetate in air unless otherwise noted. *E*/*Z* ratios are given in % *Z* isomer after *x* minutes (*λ*_exc_ = 440 nm). Cinnamyl acetate (0.2 mmol) in DCM (0.2 M) and PC (1 mol%). *E*/*Z* ratios were determined by ^1^H NMR spectroscopy. Reactions with DiICztBuDPA_4_ as the PC were performed under N_2_.

Fig. 4 shows the reaction progression for the *E*/*Z* isomerization of cinnamyl acetate in air at short times, while for DiICztBuDPA_4_, the reaction under N_2_ is shown. The conversion saturates after 30 min for DiICztBu_4_, DiICzMes_4_, and DiICztBuCz_4_, with these PCs having similar reaction rates. There is no correlation observed between Δ*E*_ST_ and either the *E*/*Z* ratio or reaction rate for this transformation. The absence of any substantive change in the post-reaction absorption spectra of DiICztBu_4_, DiICzMes_4_, and DiICztBuCz_4_ revealed that these compounds are photochemically stable to O_2_ under the reaction conditions (Fig. S46a–c). Given that O_2_ does not affect the *E*/*Z* ratio, we investigated the effect on the reaction kinetics as a function of the presence/absence of O_2_ by comparing the *E*/*Z* ratios after 5 min. The reaction rates slightly increase in the absence of O_2_, leading to slightly improved *E*/*Z* ratios of 48/52, 51/49, and 49/51 for DiICztBu_4_, DiICzMes_4_, and DiICztBuCz_4_, respectively, from those in air (53/47, 61/39, and 56/44, respectively); it is not clear why the change is more dramatic with DiICzMes_4_. Hence, O_2_ acts as an ineffective yet competitive quencher of the triplet excited state of these PCs, as the change in the *E*/*Z* ratio at early times is marginal. The contrasting results with DiICztBuDPA_4_ are discussed in the SI.

The isomerization reaction with 4CzIPN is significantly slower than with DiICztBu_4_, DiICzMes_4_, and DiICztBuCz_4_, only producing a 39/61 *E*/*Z* ratio after 60 min (Fig. 4). Thus, despite similar thermodynamic driving forces (*i.e.*, similar *E*_T_), the origin of the higher yields of the *Z* isomer and faster reaction kinetics could be due to either the higher molar absorptivity of these three MR-TADF compounds at the photoexcitation wavelength and/or an increased spectral overlap of the phosphorescence of the MR-TADF emitters with the spin-forbidden absorption of the sub. 4CzIPN is mostly photostable; however, a slight decrease in the absorbance of the CT band is observed in air, suggesting that some degradation is taking place if the reaction is carried out when O_2_ is present (Fig. S46e). Furthermore, there is a significant dependency of the kinetics of the reaction with respect to the presence/absence of O_2_. After 5 min, the *E*/*Z* ratios are 80/20 and 94/6 under N_2_ and air, respectively. These results reveal that 4CzIPN photocatalyzes this reaction much more slowly than DiICztBu_4_, DiICzMes_4_, and DiICztBuCz_4_ and that its triplet excited state is also more strongly quenched by O_2_. The excited-state lifetimes of the PCs under aerated conditions are 8, 8, 8, and 13 ns for DiICztBu_4_, DiICzMes_4_, DiICztBuCz_4_, and DiICztBuDPA_4_, respectively. These are not affected by the presence of *E*-cinnamyl acetate (*τ*_PL_ of 8, 8, 8, and 12 ns for DiICztBu_4_, DiICzMes_4_, DiICztBuCz_4_, and DiICztBuDPA_4_, respectively, Fig. S47).

We had previously assessed MR-TADF compounds DABNA-1, *t*DABNA, CzBN, and DtBuCzB as photocatalysts in the *E*/*Z* isomerization of cinnamyl acetate. Of these, CzBN produced the highest conversion to the *Z* isomer in a ratio of 19/81 after two hours in air (in THF); the ratio remained the same after 24 hours.^42^ In our previous study, we did not monitor the reaction kinetics at shorter times. Thus, CzBN yields a similar *E*/*Z* ratio as the DiICz photocatalysts (19/81 for CzBN after 2 h and 18/82 for DiICztBu_4_ after 1 h). This is consistent with their comparable *E*_T_ values (2.58 eV for CzBN and 2.58 eV for DiICztBu_4_, respectively, in 2-MeTHF at 77 K).^42^

We next investigated the *E*/*Z* isomerization of diisopropyl fumarate, which has a higher *E*_T_ of 2.7 eV,^31^ to produce the corresponding maleate (*E*_T_ = 3.1 eV)^31^ (Table S8). Despite the *E*_T_s of the PCs being lower than 2.7 eV (Table 2), both DiICztBu_4_ (*E*_T_ = 2.60 in DCM) and DiICzMes_4_ (*E*_T_ = 2.65 in DCM) photocatalyzed the reaction, producing identical *E*/*Z* ratios of 13/87 after 24 h (Table 4, entries 4 and 6). DiICztBuCz_4_ and 4CzIPN were less effective, producing *E*/*Z* ratios of 66/34 and 69/31, respectively (Table 4, entries 8 and 12). Surprisingly, it seems that the slightly lower *E*_T_ of DiICztBuCz_4_ (2.58 eV in DCM) compared to DiICzMes_4_ (*E*_T_ = 2.65 eV in DCM) and DiICztBu_4_ (*E*_T_ = 2.60 eV in DCM) is responsible for these large changes in *E*/*Z* ratios; notably, DiICztBuCz_4_ is unstable under the reaction conditions (Fig. S46c). These results also clearly illustrate the effect of solvent polarity on the magnitude of the spectral overlap between the PC and sub, and the corresponding efficiency to photocatalyze the isomerization. Despite 4CzIPN having the highest *E*_T_ of 2.73 eV in 2-MeTHF at 77 K, meaning that if these values were reflective of accessible triplet energies under the reaction conditions, then it should be able to photocatalyze the isomerization to a similar extent as the DiICz PCs. The triplet state of 4CzIPN, however, shows the greatest stabilization as a function of solvent polarity (*E*_T_ in DCM is 2.56 eV at room temperature, Table 1). This accounts for the poorer *E*/*Z* ratio of 69/31 (Table 4, entry 12). Notably, as 4CzIPN is photostable under these reaction conditions when performed under N_2_, its poor performance cannot be attributed to PC degradation (Fig. S45e).

**Table 4:** *E*/*Z* isomerization of diisopropyl fumarate^a^

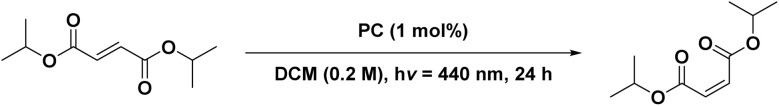			
Entry	Photocatalyst	Conditions	*E*/*Z* ratio
1	None	Air	100/0 ± 0
2	None	N_2_	100/0 ± 0
3	DiICztBu_4_	Air	95/5 ± 1
4	DiICztBu_4_	N_2_	13/87 ± 0
5	DiICzMes_4_	Air	92/8 ± 2
6	DiICzMes_4_	N_2_	13/87 ± 0
7	DiICztBuCz_4_	Air	88/12 ± 0
8	DiICztBuCz_4_	N_2_	66/34 ± 4
9	DiICztBuDPA_4_	Air	100/0 ± 0
10	DiICztBuDPA_4_	N_2_	100/0 ± 0
11	4CzIPN	Air	91/9 ± 4
12	4CzIPN	N_2_	69/31 ± 4

a Diisopropyl fumarate (0.2 mmol) in DCM (0.2 M) with 1 mol% PC loading. *E*/*Z* ratios were determined by ^1^H NMR spectroscopy. Reported yields are the mean from at least two reactions with associated standard deviations.

In contrast to cinnamyl acetate (Table 3), the *E*/*Z* isomerization of diisopropyl fumarate essentially does not proceed to any appreciable extent in the presence of O_2_, with *E*/*Z* ratios of 96/5, 93/8, 88/12, and 91/9 for DiICztBu_4_, DiICzMes_4_, DiICztBuCz_4_, and 4CzIPN, respectively (Table 4, entries 3, 5, 7, and 11). DiICztBuDPA_4_ could not photocatalyze the reaction (Table 4, entries 9 and 10), given its *E*_T_ of 2.41 eV.

**Fig. 5:**
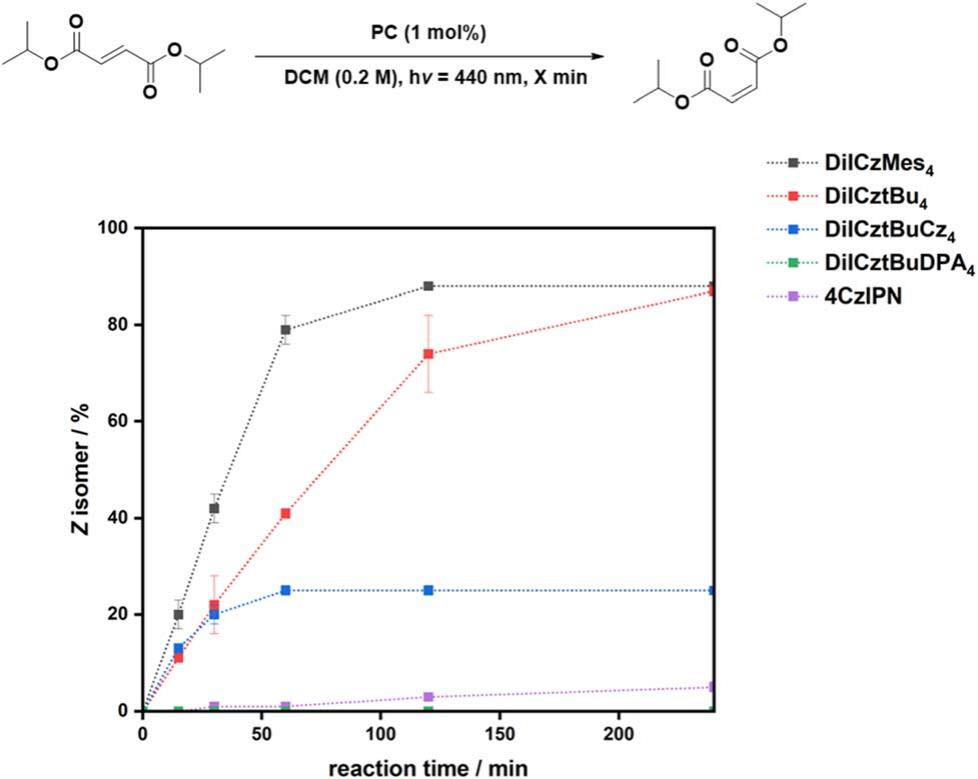
Reaction progression for the *E*/*Z* isomerization of diisopropyl fumarate under N_2_. *E*/*Z* ratios are given in % *Z* isomer after *x* minutes irradiated at 440 nm. Diisopropyl fumarate (0.2 mmol) in DCM (0.2 M) and PC (1 mol%). *E*/*Z* ratios were determined by ^1^H NMR spectroscopy.

To understand the divergence in the behavior of these two isomerization reactions in the presence of O_2_, we interrogated the reaction rates of diisopropyl fumarate under N_2_ (Fig. 5). If the rates are slower compared to those with cinnamyl acetate, then the lower *E*/*Z* ratios may be explained by competitive O_2_ quenching of the T_1_ state to that of the fumarate. Unlike the reaction with cinnamyl acetate, the reaction rates under N_2_ differ between DiICzMes_4_, DiICztBu_4_, and DiICztBuCz_4_, but still are all faster than 4CzIPN. The fastest conversion to the *Z* isomer uses DiICzMes_4_, yielding an *E*/*Z* ratio of 22/79 ± 3 after 60 min (13 : 87 after 24 h, Table 4, entry 6), while DiICztBu_4_ only produces an *E*/*Z* ratio of 60/41 ± 1 after 60 min, yet affords the same *E*/*Z* ratio after 24 h as DiICzMes_4_ (13 : 87 after 24 h, Table 4, entry 4). The reaction reaches its steady-state *E*/*Z* ratio after 2 h for DiICzMes_4_ (*E*/*Z* ratio of 12/88), while for DiICztBu_4_ it takes 4 h to reach the 13/87 *E*/*Z* ratio. These rates are slower than those with cinnamyl acetate, where the reaction was completed after 30 min. Given that both DiICzMes_4_ and DiICztBu_4_ are stable during the reaction when conducted under N_2_, photodegradation of the PC can be excluded as the origin of the slower reaction kinetics observed for DiICztBu_4_. As the phosphorescence of both DiICztBu_4_ and DiICzMes_4_ is the same, the spectral overlap between the phosphorescence spectra of these two PCs and the spin-forbidden absorption spectrum of the sub will therefore be similar. A plausible conclusion is that the *tert*-butyl groups are bulkier than mesityl groups and impede the collisional interaction more, thus adversely affecting the reaction kinetics with DiICztBu_4_. We observed that these two PCs do photodegrade when the reaction is carried out under air. Thus, there is a divergence in the photochemical outcome wherein the kinetics of the *E*/*Z* isomerization of cinnamyl acetate outcompetes photodegradation, while this is not the case with the *E*/*Z* isomerization of diisopropyl fumarate (Fig. S45a and b). Surprisingly, while the initial rate of isomerization using DiICztBuCz_4_ is comparatively as fast as using DiICzMes_4_, there is a significant off-cycle photodegradation both in air and N_2_ that effectively caps the *E*/*Z* ratio at only 66/34 ± 4 (Fig. S45c). DiICztBuDPA_4_ is photostable when the reaction is conducted under N_2_, while there are changes in the absorption spectrum when the reaction is conducted in air (Fig. S46d). Hence, the fact that the reaction does not proceed is a consequence of there being no spectral overlap between the phosphorescence of DiICztBuDPA_4_ and the spin-forbidden absorption of the substrate.

4CzIPN is significantly slower and less efficient in this isomerization reaction, producing a steady-state *E*/*Z* ratio of only 69/31 ± 4 after 24 h (Table 4, entry 12); indeed, after 4 h, there is only an *E*/*Z* ratio of 95/5 ± 1 (Table S8). Under these conditions, 4CzIPN is photostable under N_2_; however, in air, photodegradation of 4CzIPN is observed (Fig. S46e). Thus, the poor performance of 4CzIPN can be explained by the too-small spectral overlap with the fumarate substrate.

The *E*/*Z* isomerization of diisopropyl fumarate studies demonstrate that three of these PCs can catalyze PEnT reactions with substrates having *E*_T_ of 2.7 eV.^31^ Thus, we next explored the intramolecular [2 + 2] cycloaddition of norbornadiene (*E*_T_ = 2.7 eV ^52^) to quadricyclane (Table S9). Schmid *et al.* had reported a 99% yield after one hour using an anionic Ir complex having an *E*_T_ of 2.99 eV (Table 5, entry 14).

**Table 5:** [2 + 2] cycloaddition of norbornadiene^a^

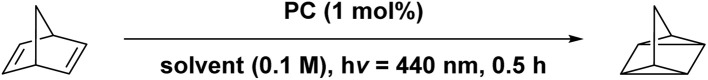			
Entry	Photocatalyst	Conditions	Yield/%
1	None	Air	0 ± 0
2	None	N_2_	0 ± 0
3	DiICztBu_4_	Air	64 ± 4
4	DiICztBu_4_	N_2_, 2 h	87 ± 1
5	DiICztBu_4_	N_2_	89 ± 2
6	DiICzMes_4_	Air	71 ± 6
7	DiICzMes_4_	N_2_	85 ± 5
8	DiICztBuCz_4_	Air	75 ± 2
9	DiICztBuCz_4_	N_2_	88 ± 0
10	DiICztBuDPA_4_	Air	0 ± 0
11	DiICztBuDPA_4_	N_2_	0 ± 0
12	4CzIPN	Air	0 ± 0
13	4CzIPN	N_2_	0 ± 0
14	[Ir(L)_2_(BCF)_2_]^−^	Degassed	99^b^

a Norbornadiene (0.2 mmol) and PC (1 mol%) in DCM (2 mL). Yields were determined by ^1^H NMR spectroscopy using 1,3,5-trimethoxybenzene as the internal standard.

b From ref. 52 in CD_3_CN, *λ*_exc_ = 415 nm after 1 h, norbornadiene (0.05 mmol), PC (0.3 mol%), CD_3_CN (0.6 mL). Reported yields are the mean from at least two reactions with associated standard deviations.

The reaction does not take place in the absence of a PC (Table 5, entries 1 and 2). After 30 min reaction time using DiICztBu_4_, quadricyclane was obtained in an 89% yield under N_2_ (Table 5, entry 5). In contrast to the *E*/*Z* isomerization of fumarate, DiICzMes_4_ and DiICztBuCz_4_ perform as well as DiICztBu_4_, yielding 85, 88, and 89%, respectively, after 30 min (Table 5, entries 5, 7, and 9). The reaction is slightly quenched in the presence of O_2_, producing yields of 64, 71, and 75% after 30 min when using DiICztBu_4_, DiICzMes_4_, and DiICztBuCz_4_, respectively (Table 5, entries 3, 6, and 8). Given the fast reaction rates, there is thus significantly less quenching of the ^3^PC* by O_2_ than what has been observed with diisopropyl fumarate. Again, it is not surprising that DiICztBuDPA_4_ does not photocatalyze the reaction, given its too low *E*_T_ (Table 5, entries 10 and 11). Similarly, given that 4CzIPN did not photoisomerize diisopropyl fumarate to diisopropyl maleate after 30 min to any appreciable extent, this PC did not photocatalyze the [2 + 2] cycloaddition of norbornadiene within 30 min (Table 5, entries 12 and 13). These results not only demonstrate the value of three of these MR-TADF PCs to photocatalyze a demanding PEnT reaction but also that *E*_T_ as a parameter is too coarse when cross-comparing different reactions using substrates with similar *E*_T_. We had previously demonstrated that DiKTa, another MR-TADF photocatalyst (*E*_T_ = 2.62 eV),^41^ could efficiently photoisomerize diisopropyl fumarate into diisopropyl maleate in a ratio of 10/90 in MeCN over 16 h, a similar ratio to that using DiICzMes_4_ (8/92); in this earlier study, the reaction progression at shorter reaction times was not analysed.^41^

The photostability of the PCs was tested under the reaction conditions under N_2_ and in air (Fig. S51). The absorption spectra of DiICztBu_4_, DiICzMes_4_, and DiICztBuCz_4_ show only a minimal decrease in intensity under N_2_, while there are greater changes with O_2_ present that suggest more photodegradation, which is consistent with the less efficient performance when the reaction is carried out in air (Fig. S51a–c). Interestingly, the small degree of photodegradation of the MR-TADF PCs in the [2 + 2] cycloaddition in air contrasts with the significant photodegradation in the *E*/*Z* isomerization with diisopropyl fumarate in air and the associated absence of product formation, despite both substrates having the same reported *E*_T_.

There is significant photodegradation observed for 4CzIPN under N_2_, and the profile is similar to that in air (Fig. S51e). The absorption band at *λ*_abs_ = 377 nm and the shoulder at *λ*_abs_ = 450 nm disappear, while a more red-shifted, less intense band at *λ*_abs_ = 520 nm appears. These results imply that the substrate reacts with 4CzIPN and that the [2 + 2] cycloaddition is likely a radical stepwise process as opposed to a concerted mechanism (Table 5, entry 12 and 13).

We then explored a PEnT reaction with a substrate having a higher *E*_T_, the sigmatropic shift of (*S*)-verbenone (*E*_T_ = 3.0 eV)^52^ to chrysanthenone (Table S10). This rearrangement has been used in the synthesis of the natural product xishacorene B, where verbenone was directly irradiated in the first step with UV light (*λ*_exc_ = 365 nm), yielding 67% chrysanthenone.^53^ Schmid *et al.* showed that this transformation can be photocatalyzed using the same iridium isocyanoborato complex as was used in the [2 + 2] cycloaddition of norbornadiene,^52^ whereupon irradiating the solution at 415 nm produced the desired product in 80% yield after 180 min (Table 6, entry 10). With DiICztBu_4_, DiICzMes_4_, and DiICztBuCz_4_ as the PCs, the yields were generally low after 24 h at 29, 22, and 18%, respectively, using 1 mol% PC (Table 6, entries 2, 4, and 6); evidently, DiICztBuDPA_4_ cannot photocatalyze this reaction due to its too low *E*_T_ (Table 6, entry 8). The yields were improved by increasing the PC loading to 5 mol%, resulting in yields of 37, 38, and 21% for DiICztBu_4_, DiICzMes_4_, and DiICztBuCz_4_, respectively (Table 6, entries 3, 5, and 7). Using 4CzIPN afforded only 5% product yield at 5 mol% PC loading (Table 6, entry 9).

**Table 6:** Sigmatropic shift of (*S*)-verbenone^a^

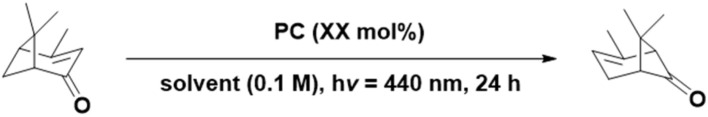			
Entry	PC	Loading/mol%	Yield/%
1	None	—	0 ± 0
2	DiICztBu_4_	1	29 ± 3
3	DiICztBu_4_	5	37 ± 1
4	DiICzMes_4_	1	22 ± 2
5	DiICzMes_4_	5	38 ± 6
6	DiICztBuCz_4_	1	18 ± 1
7	DiICztBuCz_4_	5	21 ± 2
8	DiICztBuDPA_4_	1	0 ± 0
9	4CzIPN	5	5 ± 1
10	[Ir(L)_2_(BCF)_2_]^−^	0.3	80^b^

a (*S*)-verbenone (0.2 mmol) in DCM (0.1 M) with given PC loadings. Yields were determined by ^1^H NMR spectroscopy using 1,3,5-trimethoxybenzene as the internal standard.

b From ref. 52 in CD_3_CN with *hv* = 415 nm after 3 h. Reported yields are the mean from at least two reactions with associated standard deviations.

There is essentially no change in the absorption profiles of DiICztBu_4_, DiICzMes_4_, and DiICztBuCz_4_ before and after irradiation, suggesting that any photodegradation of the PCs is not the cause for the low yields in this reaction but rather the too low triplet energy (Fig. S53a–c). 4CzIPN and DiICztBuDPA_4_ are stable under the reaction conditions (Fig. S53d and e), and the 5% yield for 4CzIPN and the 0% yield for DiICztBuDPA_4_ can be attributed to each having a T_1_ energy that is effectively too low to enable DET with any degree of efficiency.

Thus far, we have explored four intramolecular PEnT reactions. To expand the portfolio of available reactions, we next investigated an example of a bimolecular cross-coupling reaction. This is frequently achieved by combining the photocatalytic cycle with a Ni-mediated cross-coupling reaction. In such dual-catalyzed reactions, the PC can either be involved in a PET mechanism, a so-called metallaphotoredox reaction, or in a PEnT reaction to sensitize the Ni co-catalyst by DET after it has undergone oxidative addition and transmetallation steps. This photoactivation accelerates the reductive elimination of the product.^31^ One such cross-coupling reaction where the PC is purported to engage in DET is an esterification involving aryl halides being cross-coupled with carboxylic acids, where Welin *et al.* used *fac*-Ir(ppy)_3_ as the PC (*E*_T_(*fac*-Ir(ppy)_3_) = 2.58 eV).^13^ In this cross-coupling reaction, an undesired side product is lactone 2 (Table 7). Several groups have employed organic PCs such as 4DPAPN or SACR-IPTZ for these reactions (Fig. S1).^31,39^4DPAPN, after optimization of the reaction conditions, yielded 91% of 4-(trifluoromethyl)phenyl benzoate in the cross-coupling of benzoic acid with 4-bromobenzotrifluoride as substrates.^31^ With the same substrates but under slightly different conditions, Welin *et al.* reported an 86% yield using *fac*-Ir(ppy)_3_ as the PC. The reaction photocatalyzed using SACR-IPTZ yielded 99% of the coupled product between 5-bromophthalide and benzoic acid, and no protodehalogenated product was observed.^39^

**Table 7:** Energy transfer-mediated Ni-catalyzed cross-coupling esterification^a^

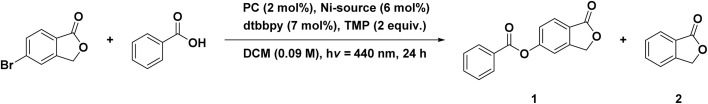				
Entry	Photocatalyst	Conditions	Yield 1/%	Yield 2/%
1	None	Ni(COD)_2_	10 ± 1	11 ± 2
2	None	NiBr_2_·glyme	0 ± 0	0 ± 0
3	DiICztBu_4_	Ni(COD)_2_	59 ± 2	28 ± 0
4	DiICztBu_4_	NiBr_2_·glyme	31 ± 4	15 ± 1
5	DiICzMes_4_	Ni(COD)_2_	65 ± 4	28 ± 3
6	DiICzMes_4_	NiBr_2_·glyme	41 ± 0	19 ± 1
7	DiICzMes_4_	[Ni(dtbbpy)(OH_2_)_4_]Cl_2_	68 ± 1	18 ± 0
8	DiICztBuCz_4_	Ni(COD)_2_	60 ± 6	31 ± 2
9	DiICztBuCz_4_	NiBr_2_·glyme	20 ± 1	20 ± 1
10	DiICztBuDPA_4_	Ni(COD)_2_	47 ± 5	14 ± 2
11	DiICztBuDPA_4_	NiBr_2_·glyme	27 ± 3	11 ± 0
12	4CzIPN	Ni(COD)_2_	43 ± 2	36 ± 4
13	4CzIPN	NiBr_2_·glyme	31 ± 0	19 ± 0
14	*fac*-Ir(ppy)_3_	Ni(COD)_2_	65 ± 1	15 ± 1
15	*fac*-Ir(ppy)_3_	NiBr_2_·glyme	49 ± 5	16 ± 1

a 5-Bromophthalide (0.188 mmol, 1.0 equiv.), benzoic acid (0.301 mmol, 1.6 equiv.), 2,2,6,6-tetramethylpiperidine (TMP) (0.375 mmol, 2.0 equiv.), Ni source (0.011 mmol, 6 mol%), 4,4′-di-*tert*-butyl-2,2′-bipyridine (dtbbpy) (0.013 mmol, 7 mol%) and PC (0.004 mmol, 2 mol%) in DCM (0.09 M). Yields were determined by ^1^H NMR spectroscopy using 1,3,5-trimethoxybenzene as the internal standard.

We first investigated the use of different Ni precursors in the presence of dtbbpy as an ancillary ligand under conditions similar to those reported by Hojo *et al.*^39^ We changed the solvent from DMF to DCM, as the DiICz PCs are more soluble in the latter.^39^ We observe a generally higher product yield in DCM when using Ni(COD)_2_ than with NiBr_2_·glyme, regardless of the choice of PC. The use of DiICzMes_4_ yielded 65% in combination with Ni(COD)_2_, while using NiBr_2_·glyme only results in a 41% yield (Table 7, entries 5 and 6). In both cases, side product 2 is observed in 30 and 19% yield (Table 7, entries 5 and 6). DiICztBu_4_, DiICzMes_4_, and DiICztBuCz_4_ perform similarly in combination with Ni(COD)_2_ with yields of 59, 65, and 60% of 1, respectively, while 2 formed in 28, 28, and 31% yield, respectively (Table 7, entries 3, 5, and 8). Changing the Ni source to NiBr_2_·glyme yielded 31/15 and 41/19% of 1 and 2 using DiICztBu_4_ and DiICzMes_4_, respectively, implying that the use of NiBr_2_·glyme results in more undesired protodehalogenated product for these two complexes, while with DiICztBuCz_4_, the reaction proceeds less readily, affording 20% of each of 1 and 2. These PCs produce higher yields of 1 in combination with Ni(COD)_2_ than using 4CzIPN (43%, Table 7, entry 12), and this is correlated to a greater amount of 2 forming (36%) with this latter PC. When *fac*-Ir(ppy)_3_ is employed as the PC in combination with Ni(COD)_2_, 65% of 1 and 15% of 2 form (Table 7, entry 14). A comparable yield is observed with DiICzMes_4_ (68/18% of 1 and 2, Table 7, entry 7), but the Ni source must be [Ni(dtbbpy)(OH_2_)_4_]Cl_2_, implying that the kinetics of the formation of the active Ni species are suboptimally aligned with the kinetics of DET with this PC. After solvent optimization with the best performing DiICz PC, DiICzMes_4_, the coupled product 1 was obtained in a 74% yield when using a 1 : 1 mixture of DMSO : toluene (Table 8, entry 4), while the control experiment with *fac*-Ir(ppy)_3_ yielded a comparable yield of 77% (Table 8, entry 6). The 1 : 1 DMSO : toluene solvent system had previously been shown to be optimal with SACR-IPTZ as the PC, yielding 99% of 1 (Table 8, entry 8).^39^ The reaction does not readily proceed in toluene, yielding only 10% product, while in DMSO an increase in the yield (64% of 1) compared to that in DCM is observed, ostensibly due to a partial suppression of the formation of 2 (Table 8, entry 1–3). Using 1 : 1 DMSO : toluene in combination with the preformed Ni complex [Ni(dtbbpy)(OH_2_)_4_]Cl_2_ and DiICzMes_4_ as the PC resulted in 81% yield of 1 and only 13% of 2 (Table 8, entry 7). Reducing the reaction time from 24 to 3 h resulted in a lower yield of 1 (54%) but not of 2 (13%) (Table 8, entry 5), while with *fac*-Ir(ppy)_3_ as the PC the product was obtained in 68% yield after 3 h (Table 8, entry 7). While the reaction with *fac*-Ir(ppy)_3_ is slightly faster than with DiICzMes_4_, effectively similar yields of 74 and 77% were obtained after 24 h using DiICzMes_4_ and *fac*-Ir(ppy)_3_, respectively.

**Table 8:** Reaction optimization of the Ni-co-catalyzed cross-coupling esterification reaction^a^

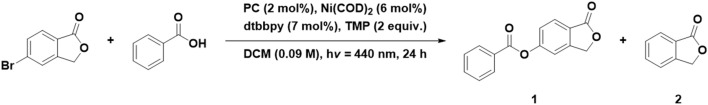					
Entry	Solvent	PC	Conditions	Yield 1/%	Yield 2/%
1	DCM	DiICzMes_4_	Ni(COD)_2_	60 ± 3	30 ± 1
2	Toluene	DiICzMes_4_	Ni(COD)_2_	10 ± 3	—
3	DMSO	DiICzMes_4_	Ni(COD)_2_	64 ± 5	21 ± 1
4	DMSO : toluene 1 : 1	DiICzMes_4_	Ni(COD)_2_	74 ± 2	15 ± 1
5	DMSO : toluene 1 : 1	DiICzMes_4_	Ni(COD)_2_, 3 h	54 ± 0	13 ± 0
6	DMSO : toluene 1 : 1	*fac*-Ir(ppy)_3_	Ni(COD)_2_	77 ± 1	15 ± 0
7	DMSO : toluene 1 : 1	*fac*-Ir(ppy)_3_	Ni(COD)_2_, 3 h	68 ± 2	16 ± 2
8	DMSO : toluene 1 : 1	DiICzMes_4_	[Ni(dtbbpy)(H_2_O)_4_]Cl_2_	81 ± 1	13 ± 1
9	DMSO : toluene 1 : 1	SACR-IPTZ^b^	Ni(COD)_2_	99	—

a 5-Bromophthalide (0.188 mmol, 1.0 equiv.), benzoic acid (0.301 mmol, 1.6 equiv.), TMP (0.375 mmol, 2.0 equiv.), Ni-source (0.011 mmol, 6 mol%), dtbbpy (0.013 mmol, 7 mol%) and PC (0.004 mmol, 2 mol%) in the respective solvent (0.09 M). Yields were determined by ^1^H NMR spectroscopy using 1,3,5-trimethoxybenzene as the internal standard.

b Literature yield taken from ref. 39 5-bromophthalide (0.188 mmol, 1.0 equiv.), benzoic acid (0.301 mmol, 1.6 equiv.), TMP (0.375 mmol, 2.0 equiv.), Ni-source (0.011 mmol, 6 mol%), dtbbpy (0.013 mmol, 7 mol%) and PC (0.004 mmol, 2 mol%) in the respective solvent (0.09 M), *λ*_exc_ = 400 nm.

## Conclusion

We have investigated four DiICz emitters as fast and efficient DET photocatalysts in five different energy transfer reactions, highlighting their ability to activate high triplet energy substrates and outperform literature reference PCs. There is scant solvent dependency of the T_1_ state energies of these four MR-TADF PCs, unlike D–A TADF PCs such as 4CzIPN. Surprisingly, we observed a dependency of the sensitivity of O_2_ quenching as a competitive triplet quencher as a function of the nature of the PEnT reaction, even when the *E*_T_ of the substrate was the same. Reactions that proceed sufficiently rapidly with these PCs show little to no O_2_ dependency on the final yield, which is a benefit of these compounds as photocatalysts. This suggests that the quenching of the excited state by O_2_ is not competitive with the DET kinetics to the substrate. Excitingly, DET to substrates with triplet energies as high as 3.0 eV is feasible with DiICztBu_4_ and DiICzMes_4_, both of which catalyze the sigmatropic shift of (*S*)-verbenone, yielding the product in around 38% yield. We concluded that there is no connection between either the yield or the reaction rate and the Δ*E*_ST_ of the PC. This study reveals the particularly valuable and wide utility of DiCzMes_4_ as a PEnT photocatalyst for substrates possessing high triplet energies.

## Author contributions

E. Z.-C. conceived and managed the project and supervised the work. D. H. synthesized DiICzMes_4_ and DiICztBuCz_4_, E. B. synthesized DiICztBuDPA_4_ and L. H. synthesized DiICztBu_4_. D. H. performed the steady-state emission and absorption measurements in toluene, T. H. measured the photoluminescence quantum yields of the four photocatalysts, and the emission lifetimes of the photocatalysts in presence of cinnamyl acetate, and L. H. performed steady-state emission and absorption measurements in DCM, DMF and EtOAc, the time-resolved photoluminescence measurements in DCM, and the determination of Δ*E*_ST_ in 2-MeTHF. L. H. carried out photocatalysis reactions and photostability studies. L. H., D. H. and E. Z.-C. contributed to the manuscript writing and discussion.

## Conflicts of interest

The authors declare no competing interests.

## Supplementary Material

SC-OLF-D5SC04014K-s001

## Data Availability

The research data supporting this publication can be accessed at https://doi.org/10.17630/22609279-98d6-4d3c-a1c1-b1f14b04b22d. Supplementary information: synthetic procedures, UV-vis absorption and photoluminescence spectra (room temperature steady-state, 77 K steady-state, and 77 K gated emission), time-resolved PL decays, photoluminescence quantum yield data, photocatalysis procedures, and photostability studies. See DOI: https://doi.org/10.1039/d5sc04014k.
