# Supplementary material for: DiICz MR-TADF emitters as potent energy transfer photocatalysts
Source: Chem Sci. 2025 Oct 28;16(48):23220–32. doi: 10.1039/d5sc04014k (PMC12587314; doi:10.1039/d5sc04014k)
Supplement: SC-016-D5SC04014K-s001 [file SC-016-D5SC04014K-s001.pdf]

Supplementary Information (SI) for Chemical Science.
This journal is © The Royal Society of Chemistry 2025

DiICz MR-TADF emitters as Potent Energy Transfer Photocatalysts

*Lea Hämmerling,[a] David Hall,[a] Eliott Blin,[a] Tabea Heil,[a] and Eli Zysman-Colman[a]**

[a]Organic Semiconductor Centre, EaStCHEM School of Chemistry, University of St Andrews, St Andrews, UK, KY16 9ST. E-mail: eli.zysman-colman@st-andrews.ac.uk

Supporting Information

Table of Contents

Supplemental Experimental Procedures S2

Synthesis S6

Photophysical measurements S39

Photocatalysis S48

References S64

Supplemental Experimental Procedures

General Synthetic Procedures. Starting materials were obtained from commercial sources and used as received. **4CzIPN**,[1] and ***fac*-Ir(ppy)$_3$**[2] were synthesized following literature procedures. Air-sensitive reactions were performed under a nitrogen atmosphere using Schlenk techniques. An MBraun SPS5 solvent purification system was used to obtain anhydrous THF, DCM, toluene, and acetonitrile (MeCN). Flash column chromatography was carried out using a Teledyne ISCO Combi*Flash*® NextGen 300+ system with RediSep® Normal-Phase Silica columns in sizes of 4 - 80 g silica packing. For analytical thin-layer-chromatography (TLC), silica plates with aluminum backings (250 µm with F-254 indicator) were used and visualised with a 254/365 nm UV lamp. HPLC was performed on a Shimadzu HPLC LC-40D instrument using a Shim-pack GIST 3µm C18 reverse phase analytical column in MeCN. GPC-HPLC was performed using a Shim-pack GPC-803 column with THF as the mobile phase. GCMS analysis was conducted using a Shimadzu QP2010SE GC-MS equipped with a Shimadzu SH-Rtx-1 column (30 m × 0.25 mm). ^{1}H and ^{13}C NMR spectra were recorded on a Bruker Advance spectrometer (400 or 500 MHz for ^{1}H and 126 MHz for ^{13}C). For the spectra analysis, the following abbreviations have been used: "s" for singlet, "d" for doublet, "t" for triplet, "q" for quartet, "m" for multiple, and "br" for broad and the spectra were referenced to residual solvent peaks. Melting points were measured using open-ended capillaries on an Electrothermal 1101D Mel-Temp apparatus and are uncorrected. High-resolution mass spectrometry (HRMS), ESI-MS and MALDI-TOF, was performed at the School of Chemistry, University of Edinburgh.

Photophysical Measurements. For the photophysical measurements, optically dilute solutions of concentrations on the order of 10^{-5} or 10^{-6} M were prepared in spectroscopic or HPLC grade solvent for absorption and emission spectra. Absorption spectra were recorded at room temperature using a Shimadzu UV-2600 double beam spectrophotometer with a 1 cm quartz cuvette. Molar absorptivity determination in DCM was verified by linear regression analysis of values obtained from five independent solutions varying concentrations from 4.23×10^{-5} M to 4.56×10^{-6} M.

For emission studies, degassed solutions were prepared via three freeze-pump-thaw cycles and spectra were taken using a home-made Schlenk quartz cuvette. An Edinburgh Instruments FS5 spectrofluorometer equipped with a 150 W ozone-free Xenon lamp and a 5 W pulsed Xenon flashlamp was used to collect steady-state emission spectra [λ_{exc}(**DiICztBu$_4$**) = 340 nm, λ_{exc}(**DiICzMes$_4$**) = 380 nm, λ_{exc}(**DiICztBuCz$_4$**) = 340 nm, λ_{exc}(**DiICztBuDPA$_4$**) = 400 nm]. An EPL-UV picosecond pulsed diode laser (Edinburgh Instruments, EPL-375, λ_{exc} = 375 nm) was used to collect time-resolved PL decays. An Open 150 mL liquid nitrogen EPR Dewar was used for ΔE_{ST} measurements of solution samples. Phosphorescence spectra were measured 1 ms after the excitation of Xenon flashlamp operating at 100 Hz with a gate time of 9 ms [λ_{exc}(**DiICztBu$_4$**) = 390 nm, λ_{exc}(**DiICzMes$_4$**) = 390 nm, λ_{exc}(**DiICztBuCz$_4$**) = 390 nm, λ_{exc}(**DiICztBuDPA$_4$**) = 400 nm, λ_{exc}(**4CzIPN**) = 380 nm]. Singlet (E_S)

and triplet (E_T) energies were determined from the corresponding high-energy onsets of the steady-state and phosphorescence spectra at 77 K in 2-MeTHF glass.

For photoluminescence quantum yield measurements, degassed solutions were prepared via three freeze-pump-thaw cycles and spectra were taken using home-made Schlenk quartz cuvette. Photoluminescence quantum yields for solutions were determined using the optically dilute method[3] in which four sample solutions with absorbances of ca. 0.08, 0.06, 0.05 and 0.03 for **DiICztBu$_4$**, of ca. 0.07, 0.06, 0.04 and 0.03 for **DiICzMes$_4$**, of ca. 0.06, 0.05, 0.04 and 0.02 for **DiICztBuCz$_4$** and ca. 0.10, 0.08, 0.06 and 0.04 for **DiICztBuDPA$_4$** were used. The Beer-Lambert law was found to remain linear at the concentrations of the solutions. For each sample, linearity between absorption and emission intensity was verified through linear regression analysis with the Pearson regression factor (R^2) for the linear fit of the data set surpassing 0.98. Individual relative quantum yield values were calculated for each solution and the values reported represent the slope obtained from the linear fit of these results. The quantum yield of the sample, Φ_{PL}, can be determined by the equation $\Phi_{PL} = (\Phi_r * \frac{A_r}{A_s} * \frac{I_s}{I_r} * \frac{n_s^2}{n_r^2})$, where *A* stands for the absorbance at the excitation wavelength (λ_{exc}: 360 nm), *I* is the integrated area under the corrected emission curve and *n* is the refractive index of the solvent with the subscripts "s" and "r" representing sample and reference respectively. Φ_r is the absolute quantum yield of the external reference quinine sulfate (Φ_r = 54.6% in 0.5 M H_2SO_4).[4] The experimental uncertainty in the emission quantum yields is conservatively estimated to be 10%, though we have found that statistically we can reproduce Φ_{PL} values to 3% relative error.

Fitting of time-resolved luminescence measurements: Time-resolved PL measurements were fitted to a sum of exponentials decay model with chi-squared (χ^2) values of between 1 and 2 using the EI software. Each component of the decay is assigned a weight (w_i), which is the contribution of the emission from each component to the total emission.

The average lifetime was then calculated using the following:

- Two exponential decay model:

$$\tau_{AVG} = \tau_1 w_1 + \tau_2 w_2$$

with weights defined as $w_1 = \frac{A1\tau_1}{A1\tau_1 + A2\tau_2}$ and $w_2 = \frac{A2\tau_2}{A1\tau_1 + A2\tau_2}$ where A1 and A2 are the preexponential-factors of each component.

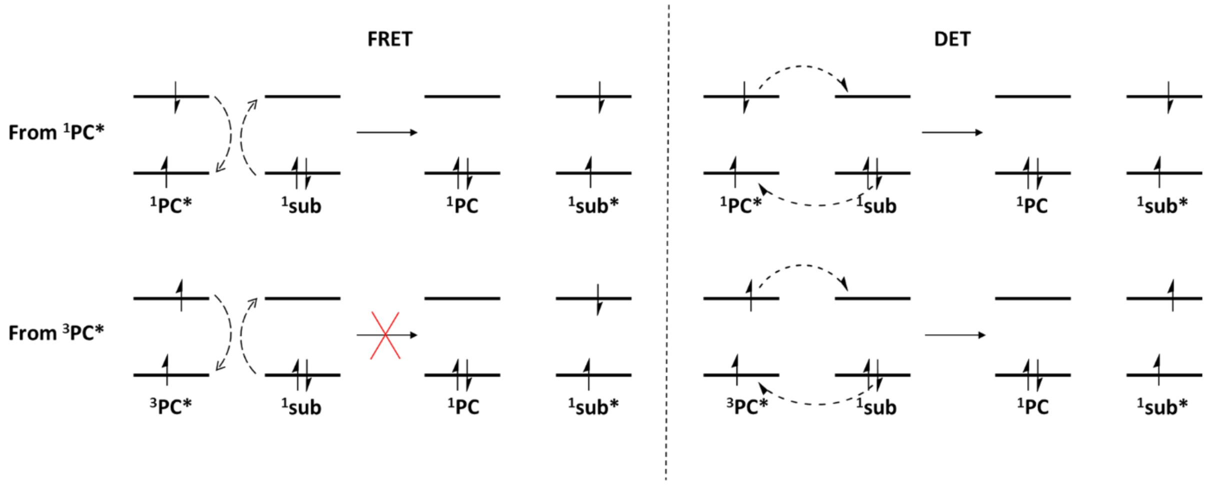

Figure **S1**. Simplified mechanism of the Förster Resonance Energy Transfer (FRET) and Dexter Energy Transfer (DET) from an excited photocatalyst either in the singlet $^{1}PC^{*}$ or the triplet $^{3}PC^{*}$ state to a substrate (sub).

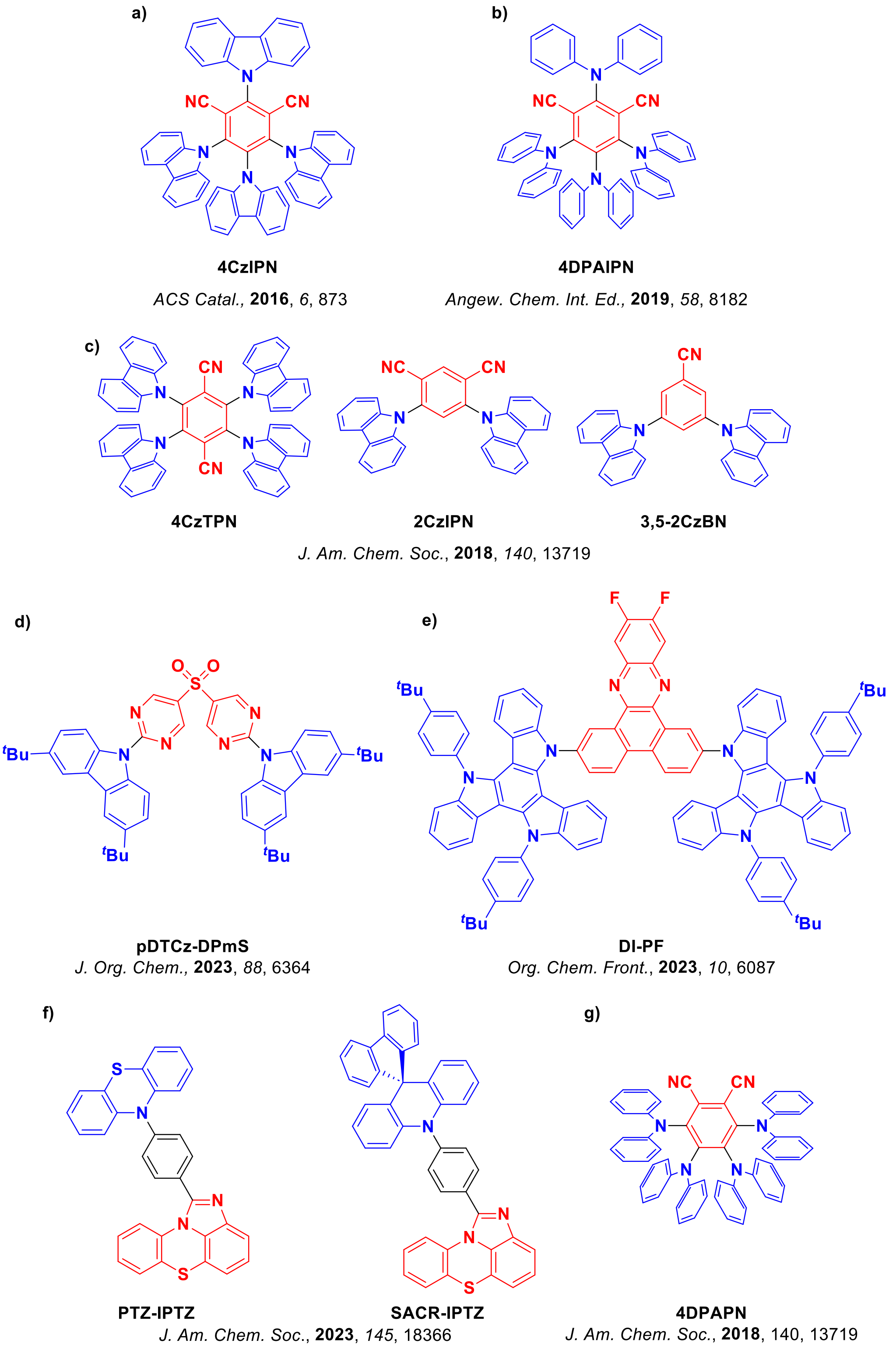

Figure **S2**. Examples of donor-acceptor thermally activated delayed fluorescence compounds used as photocatalysts. Chemical structures of a) **4CzIPN**,[5], b) **4DPAIPN**,[6] c) **4CzTPN**, **2CzIPN** and **3,5-2CzBN**,[7] d) **pDTCz-DPmS**,[8] e) **DI-PF**,[9] f) IPTZ-based photocatalysts, **PTZ-IPTZ** and **SACR-IPTZ** shown as two examples,[10] and g) **4DPAPN**.[7]

Synthesis

3,6-dibromo-9-(*tert*-butyldimethylsilyl)-9*H*-carbazole

3,6-dibromo-9-(*tert*-butyldimethylsilyl)-9*H*-carbazole has been synthesized following an adapted literature procedure.[11]

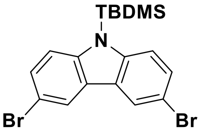

NaH (60 % in mineral oil, 5.91 g, 147.7 mmol, 1.2 equiv.) was added to dry THF (65 mL) under a nitrogen atmosphere. 3,6-Dibromo-9*H*-carbazole (40.0 g, 123.1 mmol, 1.0 equiv.) was added portion-wise and the mixture stirred at room temperature for 30 min. To this solution was added *tert*-butyl(chloro)dimethlysilane (22.3 g, 147.7 mmol, 1.2 equiv.) and the reaction stirred for 2 h. Water (50 mL) was added to quench the reaction before MeOH (100 mL) was added to form a precipitate, which was filtered off and washed with MeOH. The product was obtained as a white solid. **Yield:** 88% (47.5 g). **Mp:** 167 – 170 °C. **^{1}H NMR (400 MHz, $CDCl_3$) δ (ppm):** 8.11 (t, *J* = 1.3 Hz, 2H), 7.46 (d, *J* = 1.2 Hz, 4H), 1.01 (s, 9H), 0.74 (s, 6H). **^{13}C NMR (126 MHz, $CDCl_3$) δ (ppm):** 144.19, 128.80, 127.24, 122.87, 115.69, 113.00, 26.58, 20.67, -1.16. The characterization matches that previously reported.[11]

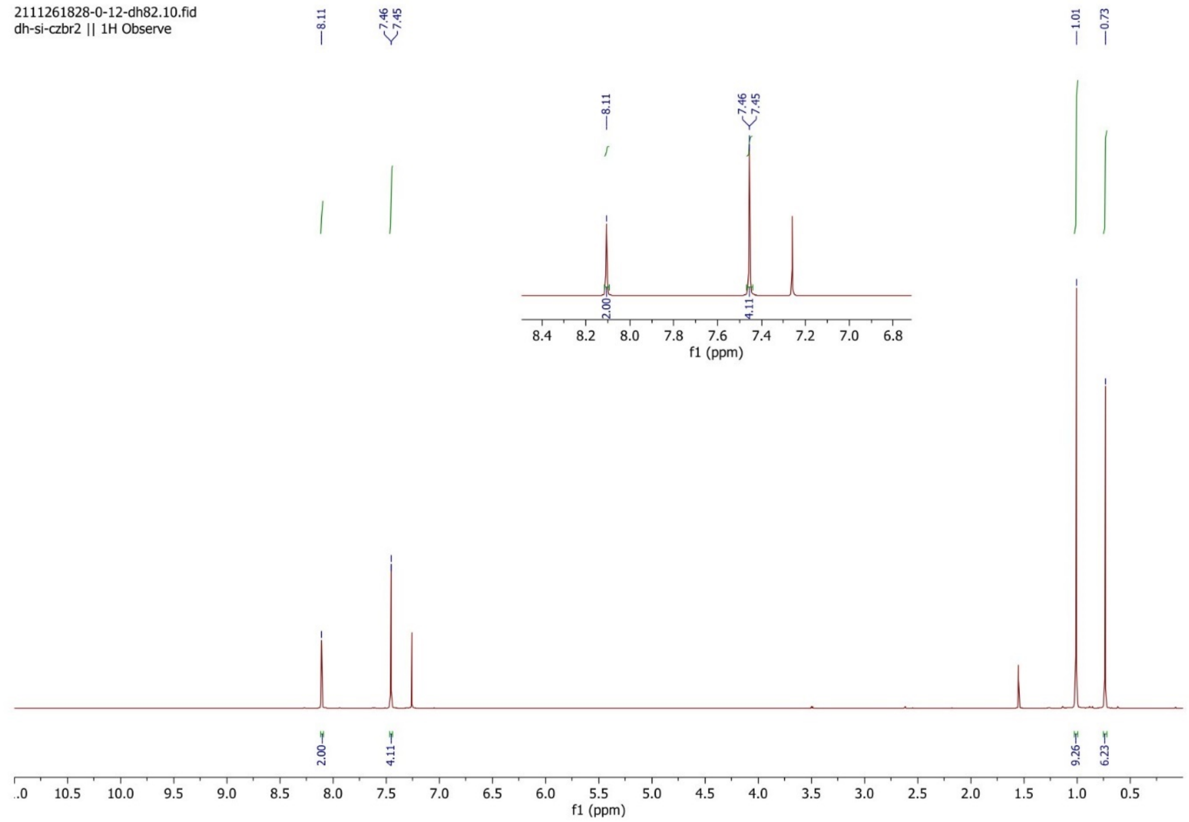

Figure **S3**. ^{1}H NMR spectrum of 3,6-dibromo-9-(*tert*-butyldimethylsilyl)-9*H*-carbazole in $CDCl_3$.

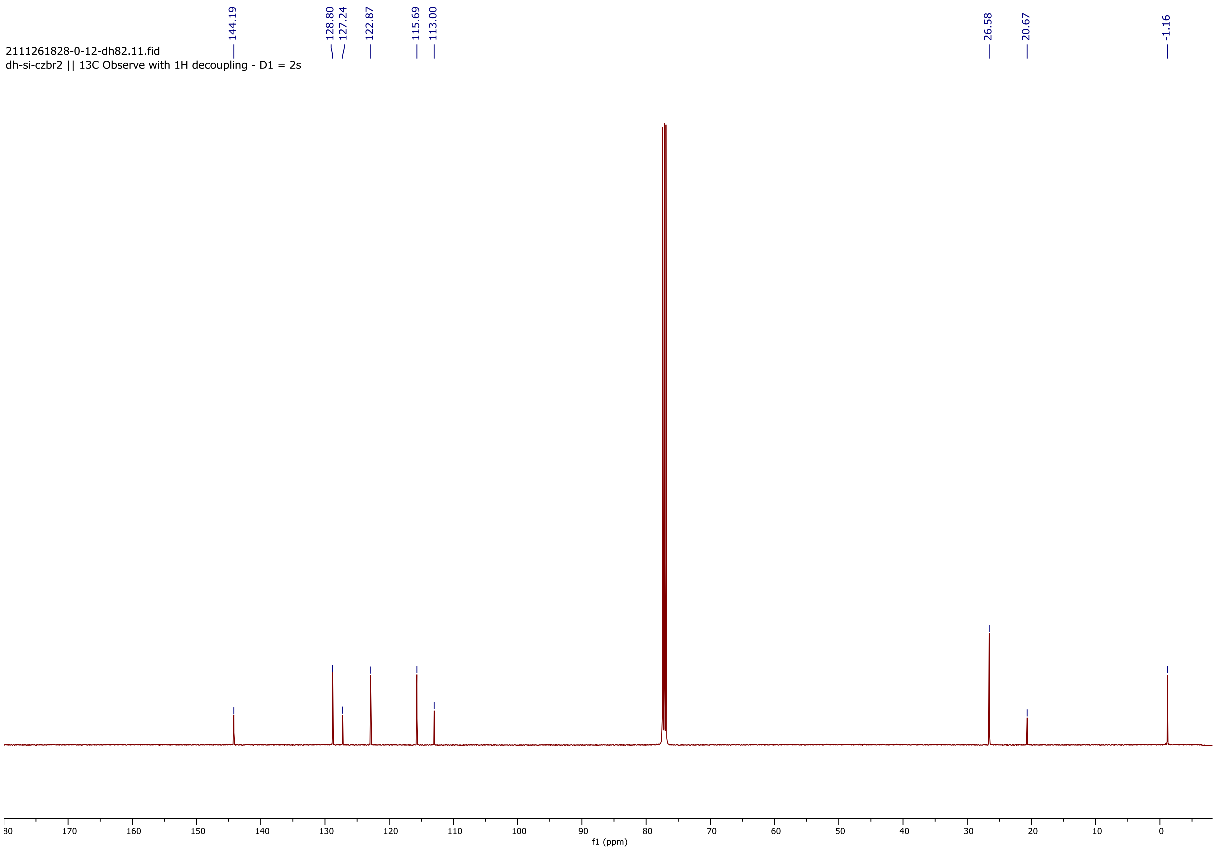

Figure **S4**. ^{13}C NMR spectrum of 3,6-dibromo-9-(*tert*-butyldimethylsilyl)-9*H*-carbazole in $CDCl_3$.

3,3'',6,6''-tetra-*tert*-butyl-9'H-9,3':6',9''-tercarbazole

3,3'',6,6''-tetra-*tert*-butyl-9'H-9,3':6',9''-tercarbazole has been synthesized following an adapted literature procedure.[12]

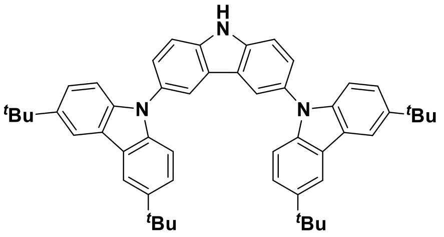

To an oven dried Schlenk purged with nitrogen was added toluene (500 mL). The solution was degassed with nitrogen bubbling for 15 min. NaO^{t}Bu (26.25 g, 273.2 mmol, 4.0 equiv.), XPhos (1.30 g, 2.73 mmol, 0.04 equiv.), 3,6-di-*tert*-butyl-9*H*-carbazole (39.12 g, 140.0 mmol, 2.05 equiv.) and 3,6-dibromo-9-(*tert*-butyldimethylsilyl)-9*H*-carbazole (30.00 g, 68.3 mmol, 1.0 equiv.) were added under a positive flow of nitrogen. The resulting solution was degassed by nitrogen bubbled for 15 min. Pd_2(dba)3 (1.88 g, 2.05 mmol, 0.03 equiv.) was added, and nitrogen was bubbled through the mixture for 5 min. The resulting solution was heated at 120 °C for 3 days. The reaction was cooled and filtered through a silica plug and washed with THF (4 × 100 mL). The resulting solution was concentrated under reduced pressure to produce a black oil. The black oil was dissolved in THF (500 mL) and tetrabutylammonium fluoride trihydrate (21.43 g, 82.0 mmol, 1.2 equiv.) was added in portions and the resulting solution was stirred under air for 2 h. The mixture was concentrated under reduced pressure. Methanol was added (500 mL) and the mixture was sonicated for 30 min and subsequently filtered. The filtered solid was washed with EtOAc (3 × 50 mL). The solid was oven dried at 80 °C for 3 h to afford 3,3'',6,6''-tetra-*tert*-

butyl-9'H-9,3':6',9''-tercarbazole as an off-white solid. **Yield:** 79% (39.0 g). **Mp:** decomposed at 366 °C (lit. Mp: >300 °C).[13]

^{1}H NMR (500 MHz, $CDCl_3$) δ (ppm): 8.42 (s, 1H), 8.17 (d, *J* = 1.6 Hz, 2H), 8.16 (d, *J* = 1.6 Hz, 4H), 7.67 (d, *J* = 8.5 Hz, 2H), 7.61 (dd, *J* = 8.5, 1.9 Hz, 2H), 7.45 (dd, *J* = 8.6, 1.8 Hz, 4H), 7.31 (d, *J* = 8.6 Hz, 4H), 1.47 (s, 36H). **^{13}C NMR (126 MHz, $CDCl_3$) δ (ppm):** 142.64, 140.33, 139.18, 130.59, 126.10, 124.24, 123.69, 123.21, 119.56, 116.32, 112.01, 109.25, 34.87, 32.19. The characterization matches that previously reported.[13]

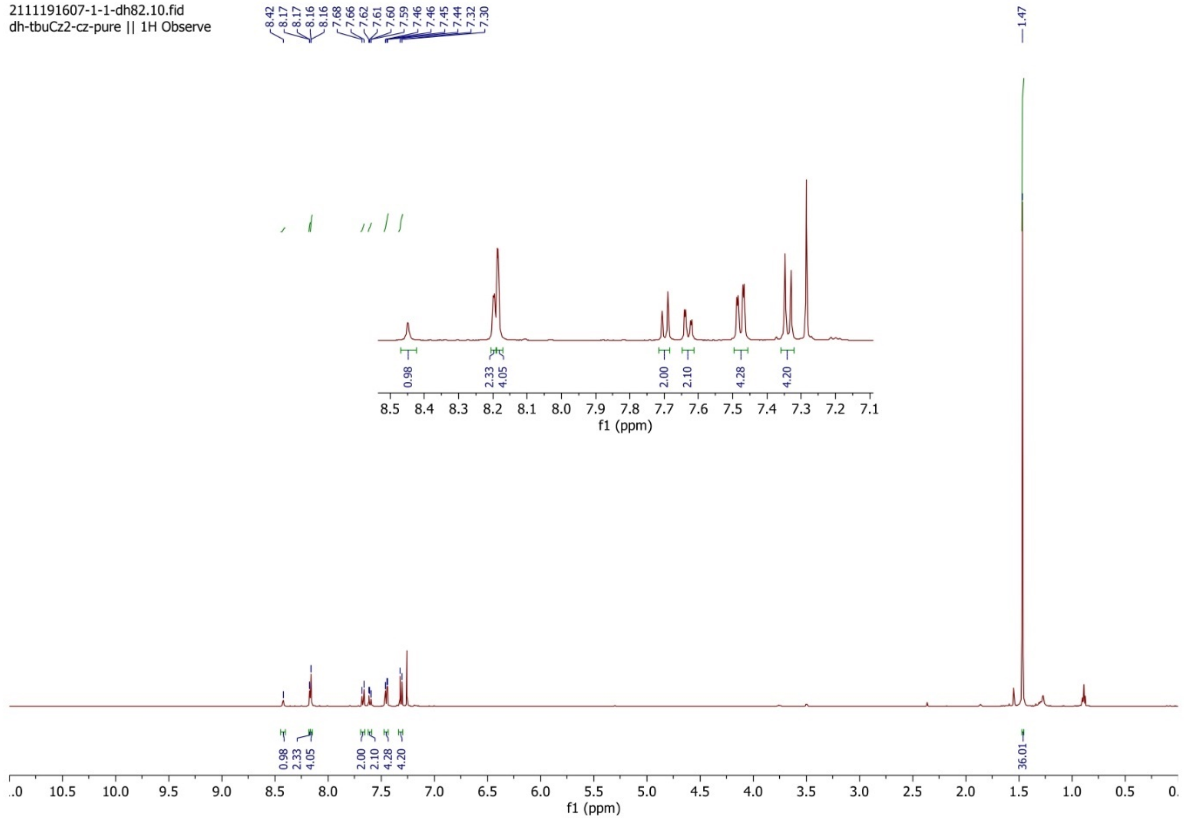

Figure **S5**. ^{1}H NMR spectrum of 3,3'',6,6''-tetra-*tert*-butyl-9'H-9,3':6',9''-tercarbazole in $CDCl_3$.

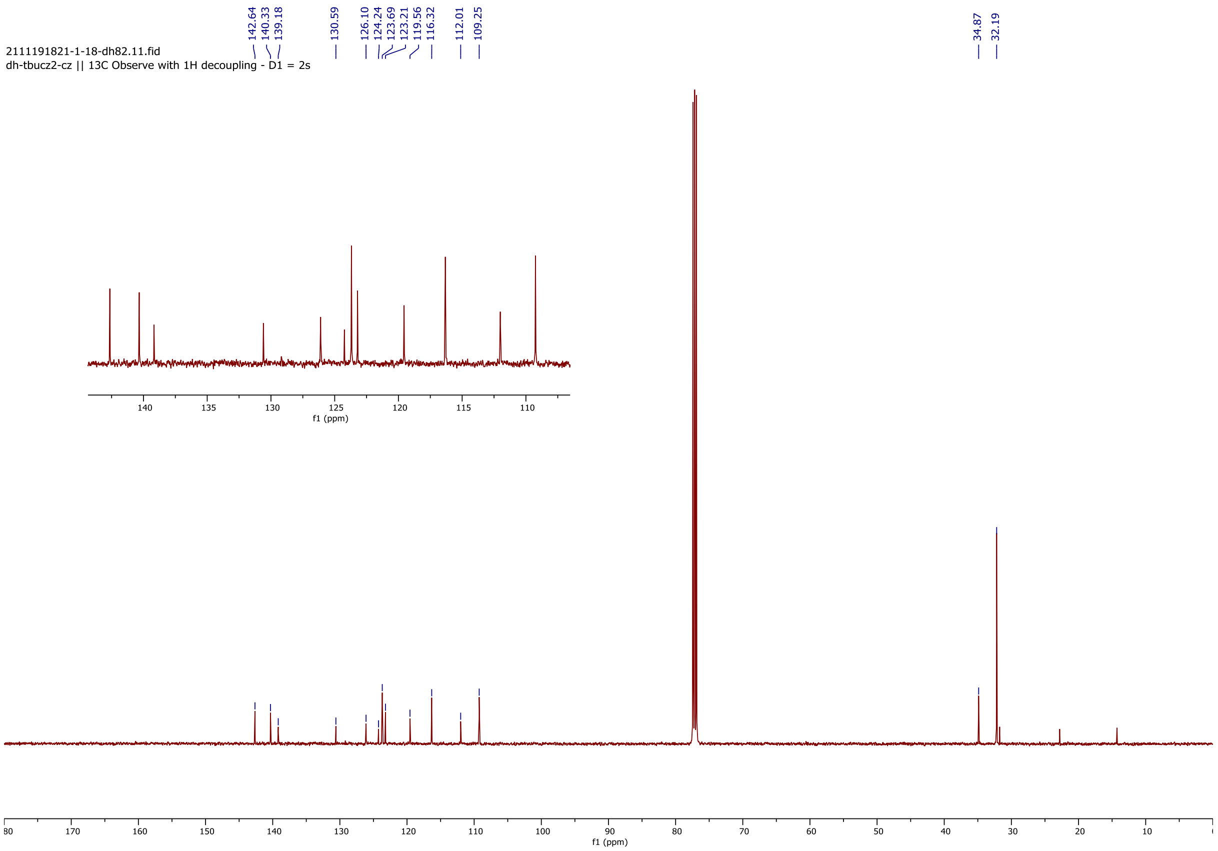

Figure **S6**. ^{13}C NMR spectrum of 3,3'',6,6''-tetra-*tert*-butyl-9'H-9,3':6',9''-tercarbazole in $CDCl_3$.

***N*^{3},*N*^{3},*N*^{6},*N*^{6}-tetrakis(4-(*tert*-butyl)phenyl)-9-(*tert*-butyldimethylsilyl)-9*H*-carbazole-3,6-diamine**

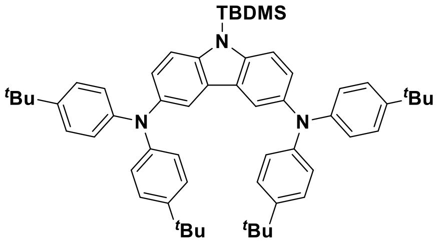

To an oven-dried Schlenk flask were added NaO^{t}Bu (1.64 g, 17.07 mmol, 3.0 equiv.) and 4,4'-di-*tert*-butyldiphenylamine (3.28 g, 11.67 mmol, 2.1 equiv.), and three vacuum/nitrogen cycles were performed. Dry xylene (115 mL) was added and the solution deoxygenated by bubbling of N_2 for 20 min. Then the 3,6-dibromo-9-(*tert*-butyldimethylsilyl)-9*H*-carbazole (2.50 g, 5.69 mmol, 1.0 equiv.) is added and again the solution bubbled with N_2 for further 20 min. Finally, tri-*tert*-butyl phosphonium tetrafluoroborate (247.70 mg, 0.85 mmol, 0.15 equiv.) and $Pd(OAc)_2$ (63.89 mg, 0.28 µmol, 0.05 equiv.) were added under a positive flow of nitrogen and the solution then stirred at 130 °C for 24 h. The solution was quenched with water (50 mL) and extracted with EtOAc (3 x 100 mL). The combined organic layers were dried over $MgSO_4$ and the solvent removed under reduced pressure. The crude product was purified by column chromatography (EtOAc:petroleum ether 5:95). The product was obtained in an off white solid. **Yield**: 52% (2.50 g). **R*$_f$***:** 0.32 (EtOAc:petroleum ether, 5:95). **Mp:** 233 – 236 °C (lit. Mp: 235 – 239 °C).[12]

^{1}H NMR (500 MHz, $CDCl_3$) δ (ppm): 7.70 (s, 2H), 7.47 (d, *J* = 8.9 Hz, 2H), 7.20 (d, *J* = 8.7 Hz, 8H), 7.14 (dd, *J* = 8.9, 2.3 Hz, 2H), 6.99 (d, *J* = 8.7 Hz, 8H), 1.29 (s, 36H), 1.07 (s, 9H), 0.72 (s, 6H).

^{13}C NMR (126 MHz, CDCl$_3$) δ (ppm): 146.10, 144.20, 142.54, 140.47, 127.16, 125.92, 124.98, 122.26, 117.69, 114.97, 68.13, 34.29, 31.61, 26.73, 20.72, -1.20. The characterization matches that previously reported.[12]

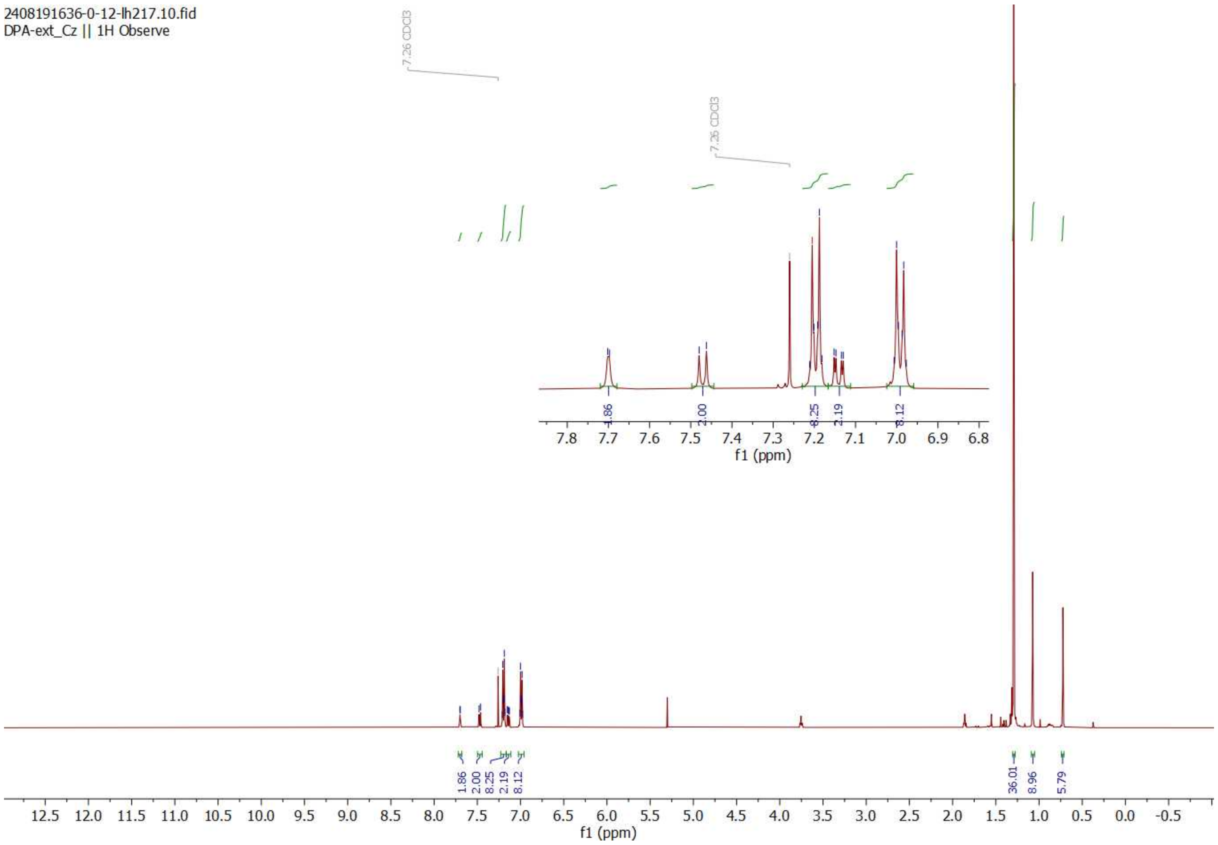

Figure **S7**. ^{1}H NMR spectrum of N^3,N^3,N^6,N^6-tetrakis(4-(*tert*-butyl)phenyl)-9-(*tert*-butyldimethylsilyl)-9*H*-carbazole-3,6-diamine in CDCl$_3$.

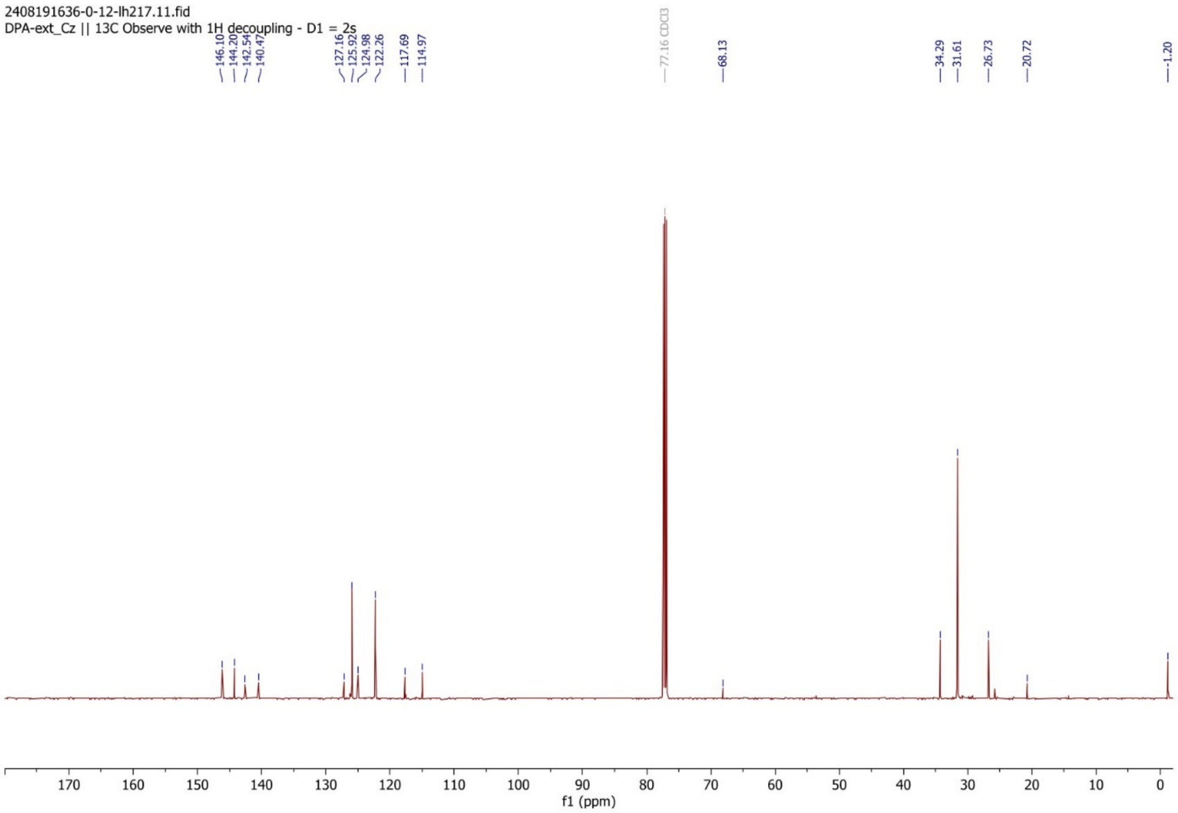

Figure **S8**. ^{13}C NMR spectrum of N^3,N^3,N^6,N^6-tetrakis(4-(*tert*-butyl)phenyl)-9-(*tert*-butyldimethylsilyl)-9*H*-carbazole-3,6-diamine in $CDCl_3$.

***N*^{3},*N*^{3},*N*^{6},*N*^{6}-tetrakis(4-(*tert*-butyl)phenyl)-9*H*-carbazole-3,6-diamine**

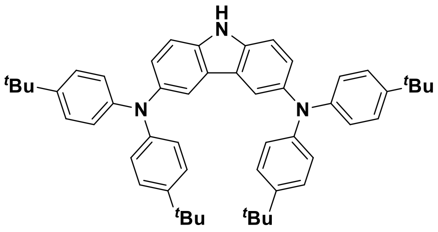

To a flask with toluene (15 mL) were added N^3,N^3,N^6,N^6-tetrakis(4-(*tert*-butyl)phenyl)-9-(*tert*-butyldimethylsilyl)-9*H*-carbazole-3,6-diamine (2.50 g, 2.98 mmol, 1.0 equiv.) and tetrabutylammonium fluoride•$3H_2O$ (1.13 g, 3.57 mmol, 1.2 equiv.), and the mixture stirred at room temperature for 2 h. The reaction was quenched with water (25 mL) and extracted with EtOAc (3 x 75 mL). The combined organic fractions were dried over $MgSO_4$ and the solvent removed under reduced pressure. The product was obtained as an off white solid. **Yield**: 83% (1.80 g). **Mp:** Decomposed at 319 − 322 °C (lit. Mp: 316 – 319 °C).[12]

^{1}H NMR (400 MHz, DMSO-d$_6$) δ (ppm): 11.30 (s, 1H), 7.87-7.78 (m, 2H), 7.47 (d, *J* = 8.6 Hz, 2H), 7.21 (d, *J* = 8.5 Hz, 8H), 7.13 (d, *J* = 9.2 Hz, 2H), 6.85 (d, *J* = 8.4 Hz, 8H), 1.23 (s, 36H). The characterization matches that previous reported. ^{13}C NMR spectrum could not be obtained due to too poor solubility, which matches the previous observation.[12]

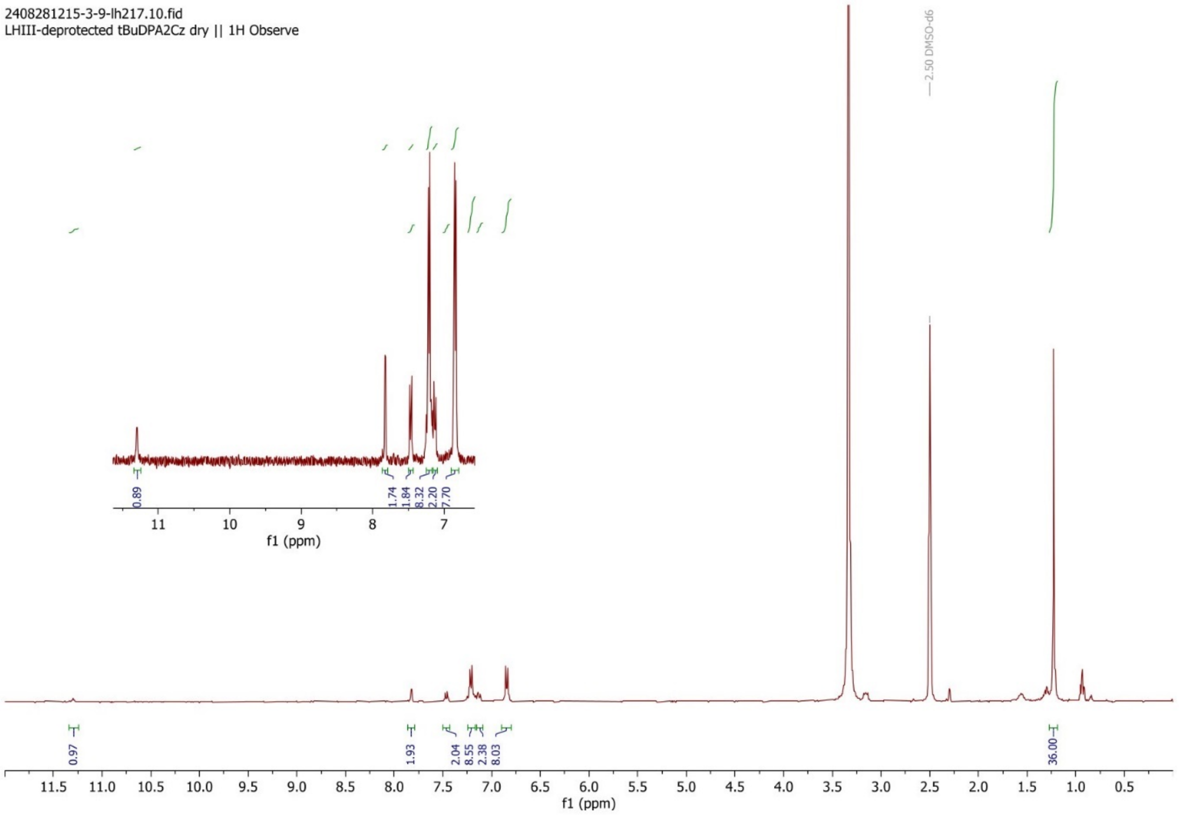

Figure **S9**. ^{1}H NMR spectrum of *N*^{3},*N*^{3},*N*^{6},*N*^{6}-tetrakis(4-(*tert*-butyl)phenyl)-9*H*-carbazole-3,6-diamine in $CDCl_3$.

General Procedure 1: SNAr coupling to 1,4-dibromo-2,5-difluorobenzene using NaH

To an oven dried Schlenk flask was added NaH (60% dispersed in mineral oil, 2.5 equiv.) and then dry DMF to reach a concentration of 0.12 M. The flask was backfilled with nitrogen. The mixture was cooled to 0 °C and the corresponding amine (2.5 equiv.) was added under a positive flow of nitrogen in portions. The solution was allowed to warm to room temperature and then stirred for 1 h before 1,4-dibromo-2,5-difluorobenzene (1 equiv.) was added. The mixture was stirred at 50 °C for 18 h under a nitrogen atmosphere. After the solution was cooled to room temperature, water (100 mL) was slowly added and followed by EtOAc (100 mL). The layers were separated, and the aqueous phase was extracted with EtOAc (3 × 100 mL). The organic layers were combined, dried over $MgSO_4$, filtered and the solvent was concentrated under reduced pressure to afford the crude residue.

9,9'-(2,5-dibromo-1,4-phenylene)bis(3,6-di-*tert*-butyl-9*H*-carbazole)

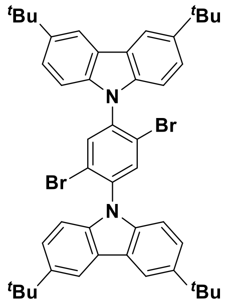

Reaction with 3,6-di-*tert*-butyl-9*H*-carbazole (2.57 g, 9.19 mmol, 2.5 equiv.) using General Procedure 1. The crude residue was purified by silica gel column chromatography (DCM:Hexane 2:98). The corresponding fractions were combined and concentrated under reduced pressure to afford the desired product as a white solid. **Yield**: 86% (2.50 g). **R_f** = 0.14 (DCM:Hexane 2:98). **Mp:** >400 °C.

^{1}H NMR (400 MHz, $CDCl_3$) δ (ppm): 8.18 (d, *J* = 1.3 Hz, 4H), 7.93 (s, 2H), 7.53 (dd, *J* = 8.6, 1.9 Hz, 4H), 7.17 (d, *J* = 8.6 Hz, 4H), 1.49 (s, 36H). The ^{1}H NMR matches that previously reported.[14] **^{13}C NMR (126 MHz, $CDCl_3$) δ (ppm):** 143.71, 139.17, 138.59, 135.73, 124.02, 123.78, 122.86, 116.71, 109.72, 34.96, 32.17. No ^{13}C NMR spectrum has been reported in the literature.

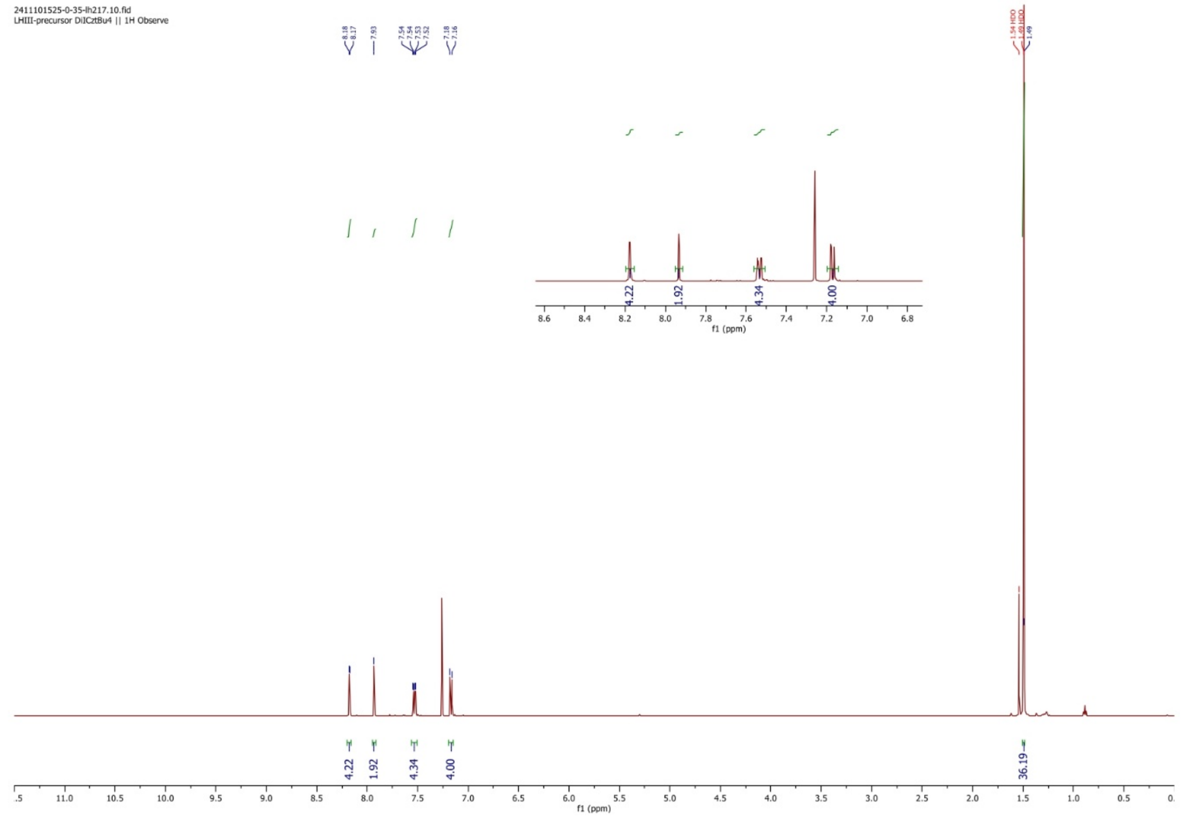

Figure **S10**. ^{1}H NMR spectrum of 9,9'-(2,5-dibromo-1,4-phenylene)bis(3,6-di-*tert*-butyl-9*H*-carbazole) in $CDCl_3$.

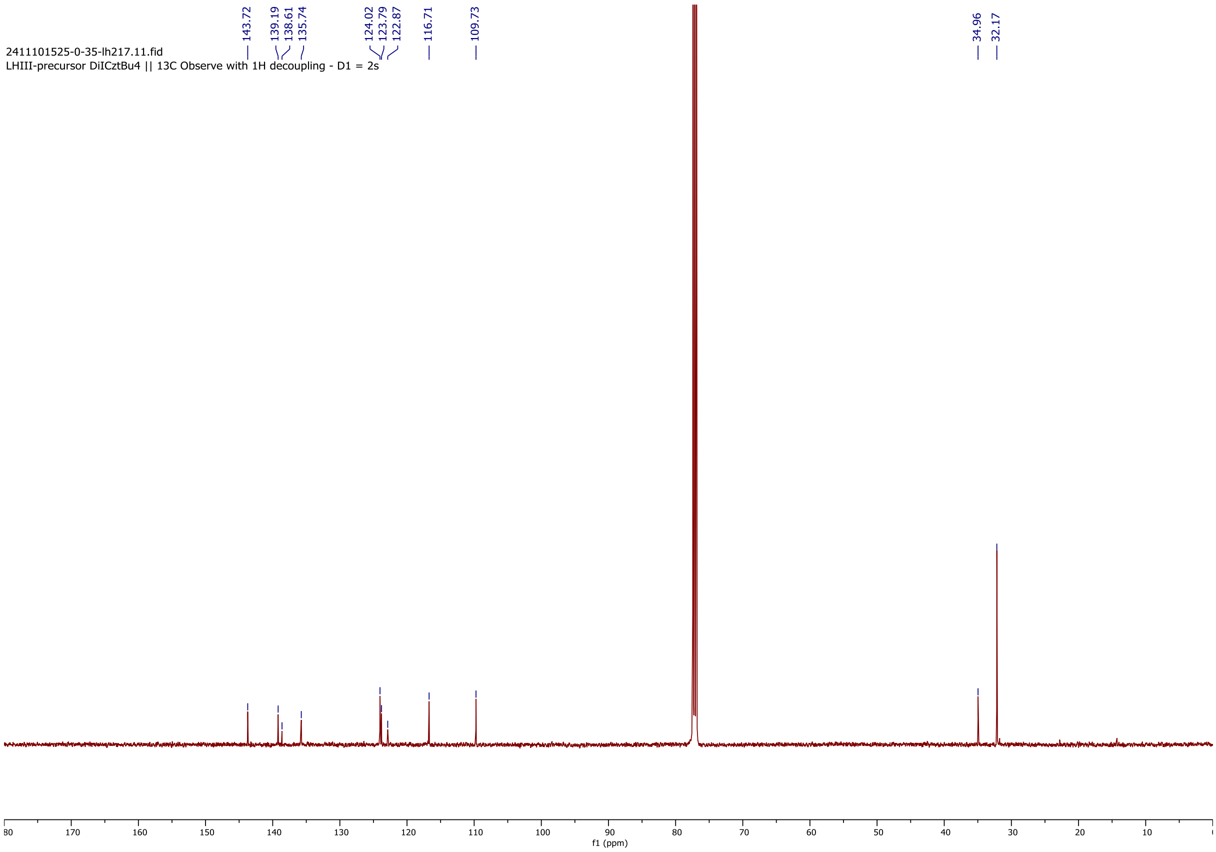

Figure **S11**. ^{13}C NMR spectrum of 9,9'-(2,5-dibromo-1,4-phenylene)bis(3,6-di-*tert*-butyl-9*H*-carbazole) in $CDCl_3$.

General Procedure 2: Oxidative ring closing

To a heat gun-dried Schlenk flask backfilled with nitrogen were added the product from General Procedure 1 (1.0 equiv.) and K_2CO_3 (10 equiv.) under a positive flow of nitrogen. Dry dimethylacetamide was then added to reach a concentration of 0.08 M. The solution was degassed with nitrogen bubbling for 20 min before tetrabutylammonium bromide (2.0 equiv.) was added, and again the reaction mixture was degassed with nitrogen bubbling for another 20 min. $Pd(OAc)_2$ (0.5 equiv.) and PPh_3 (1.0 equiv.) were added and the solution under a positive nitrogen flow. The mixture was heated at 160 °C for 48 h under a nitrogen atmosphere. The mixture was allowed to cool to room temperature before water (100 mL) and EtOAc (150 mL) were added and the phases separated. The aqueous phase was extracted with EtOAc (3 × 100 mL). The combined organic layers were washed with brine (75 mL) before being dried over $MgSO_4$. The mixture was filtered, and the solvent was concentrated under reduced pressure to afford the crude residue.

DiICztBu$_4$

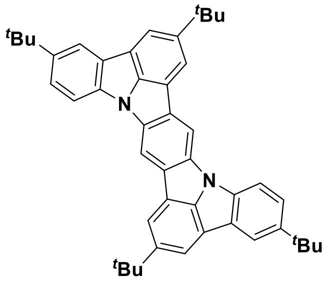

Reaction with 9,9'-(2,5-dibromo-1,4-phenylene)bis(3,6-di-*tert*-butyl-9*H*-carbazole) (1.50 g, 1.90 mmol, 1.0 equiv.) using General Procedure 2. The crude residue was purified via column chromatography (DCM:Petroleum ether 0 – 30% DCM). The resulting solid was recrystallised from a toluene:MeOH mixture (1:1, 10 mL). The reaction mixture was filtered to afford the product as a pale yellow solid. **Yield:** 34% (0.41 g). **R_f:** 0.2 (DCM:Petroleum ether 3:97). **Mp:** > 400 °C (lit. Mp: >300°C).[15] **HPLC:** 99% pure (100% MeCN with a retention time of 11.1 min).

^{1}H NMR (500 MHz, CDCl$_3$) δ (ppm): 8.57 (s, 2H), 8.28 (d, *J* = 1.1 Hz, 2H), 8.21 (d, *J* = 1.2 Hz, 4H), 7.99 (d, *J* = 8.5 Hz, 2H), 7.65 (dd, *J* = 8.5, 2.0 Hz, 2H), 1.63 (s, 18H), 1.52 (s, 18H). **^{13}C NMR (126 MHz, CDCl$_3$) δ (ppm):** 146.67, 144.58, 144.11, 137.42, 135.21, 130.03, 129.48, 124.14, 119.86, 118.34, 118.24, 116.94, 116.47, 111.56, 106.29, 36.17, 35.06, 33.06, 32.10. The characterization matches that previously reported.[15]

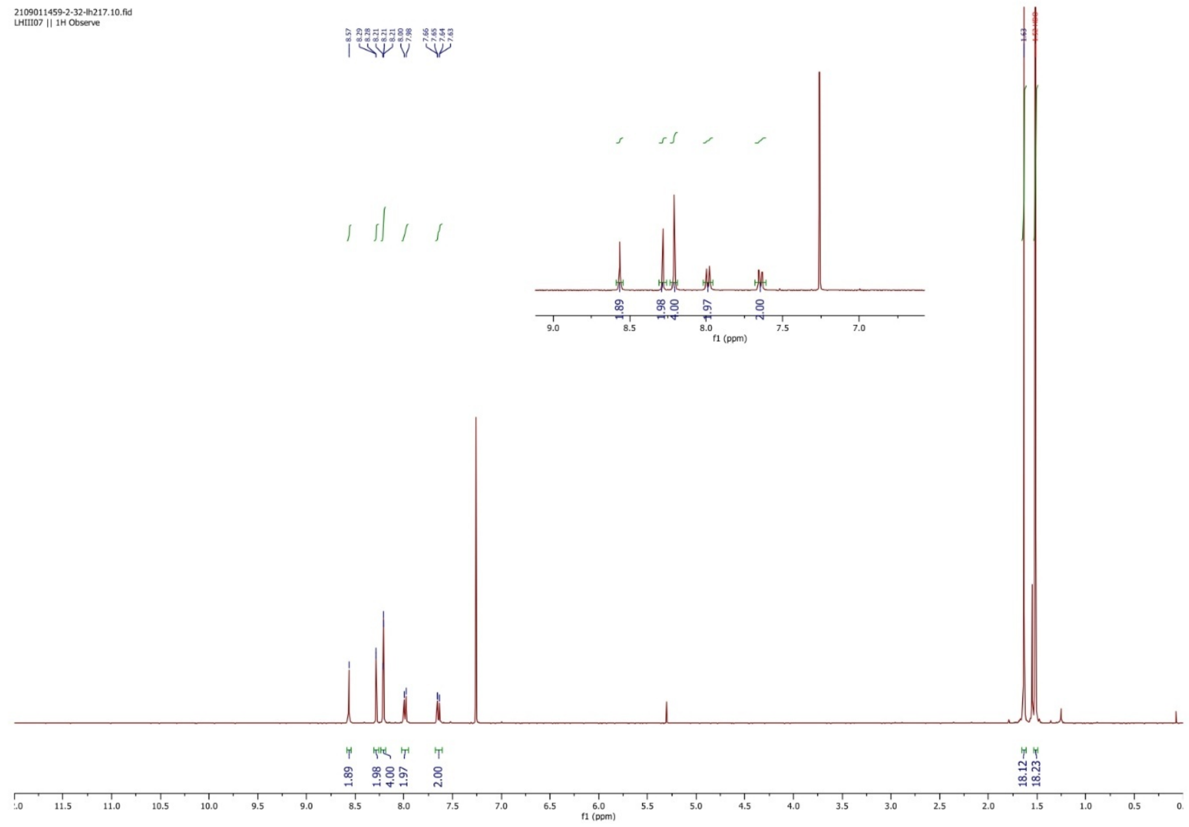

Figure **S12**. ^{1}H NMR spectrum of **DiICztBu$_4$** in CDCl$_3$.

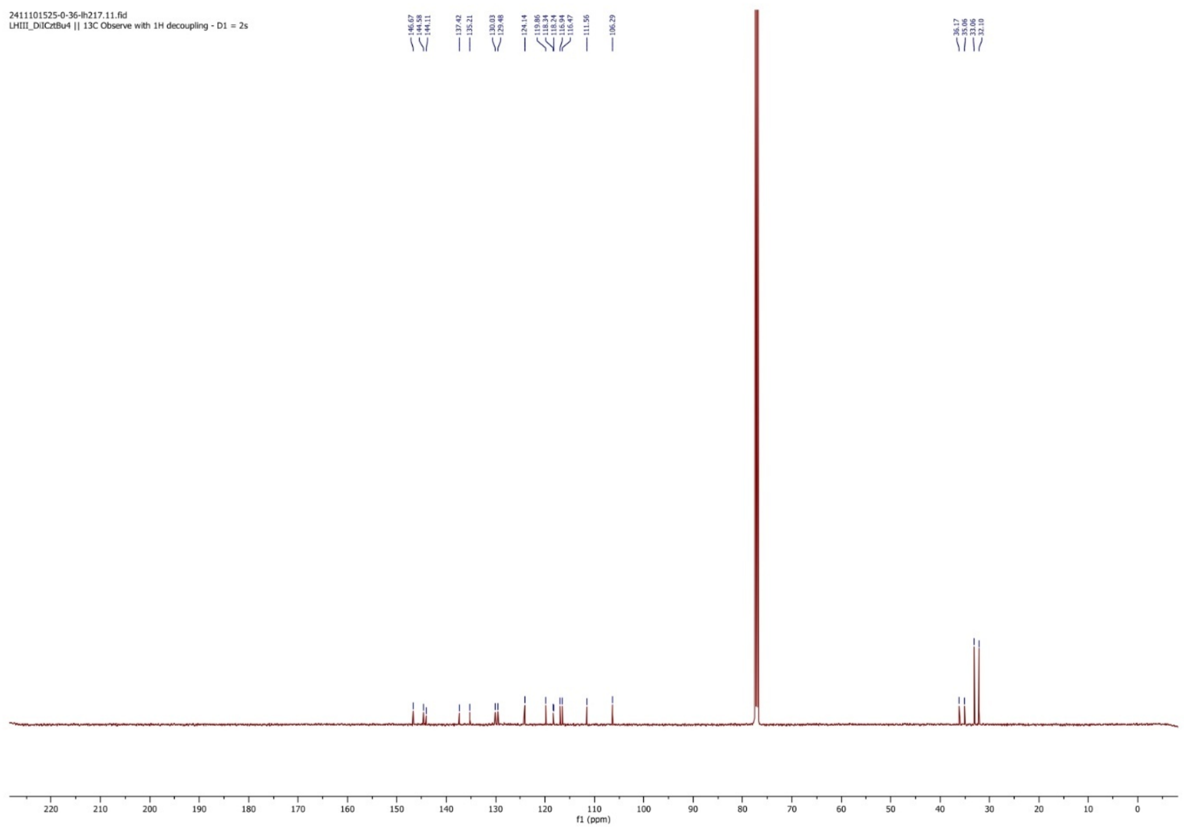

Figure **S13**. ^{13}C NMR spectrum of **DiICztBu$_4$** in $CDCl_3$.

02/09/2021 15:08:27 Page 1 / 1

HPLC Trace Report02Sep2021

<Sample Information>

Sample Name	: LHIII07 prec		
Sample ID	:		
Method Filename	: 100% Acetonitrile B 20 mins.lcm		
Batch Filename	: 26082021.lcb		
Vial #	: 2-20	Sample Type	: Unknown
Injection Volume	: 5 uL		
Date Acquired	: 02/09/2021 12:47:05	Acquired by	: System Administrator
Date Processed	: 02/09/2021 13:07:07	Processed by	: System Administrator

<Chromatogram>

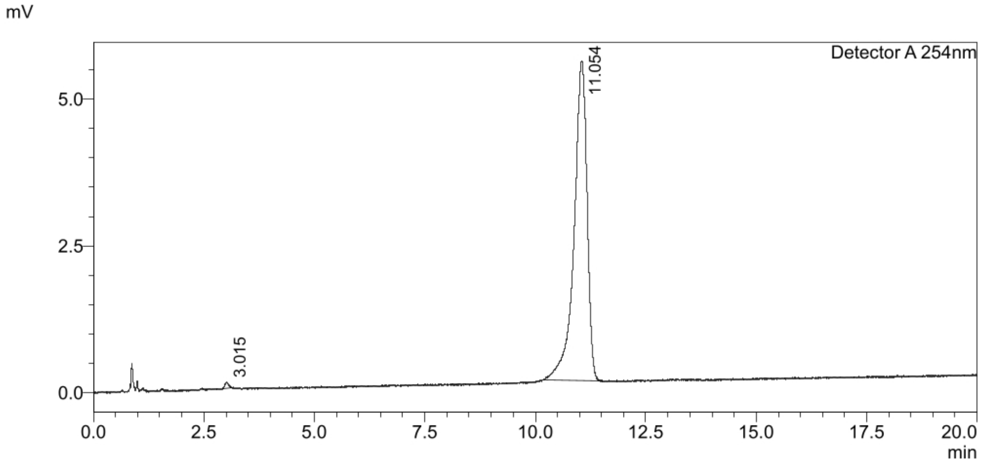

<Peak Table>

Detector A 254nm

Peak#	Ret. Time	Area	Height	Area%	Area/Height	Width at 5% Height
1	3.015	583	99	0.521	5.870	0.168
2	11.054	111314	5446	99.479	20.439	0.804
Total		111896	5545	100.000		

Figure **S14**. HPLC trace of **DiICztBu$_4$**.

9,9'-(2,5-dibromo-1,4-phenylene)bis(3,6-dimesityl-9*H*-carbazole)

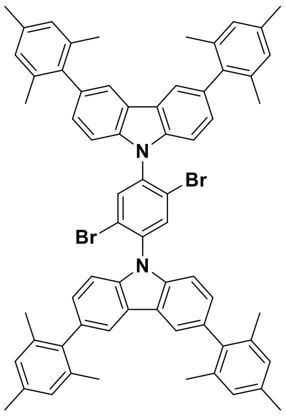

Reaction with 3,6-dimesityl-9*H*-carbazole (0.52 mg, 1.90 mmol, 1.0 equiv.) using General Procedure 1. The crude residue was then purified by silica gel column chromatography (DCM:hexane 10:90). The corresponding fractions were combined and concentrated under reduced pressure to afford the desired product. The product was obtained as a white solid. **Yield:** 73% (1.40 g). **R_f :** 0.25 (DCM:hexane 10:90). **Mp:** 276 – 280 °C, (lit. Mp: 279 – 283 °C).[16] **^{1}H NMR (500 MHz, $CDCl_3$) δ (ppm):** 8.15 (s, 2H), 7.89 (d, J = 1.6 Hz, 4H), 7.34 (d, J = 8.3 Hz, 4H), 7.27 (dd, J = 8.3, 1.6 Hz, 4H), 7.01 (s, 8H), 2.38 (s, 12H), 2.12 (s, 12H), 2.10 (s, 12H). **^{13}C NMR (126 MHz, $CDCl_3$) δ (ppm):** 139.81, 139.46, 138.62, 136.86, 136.76, 136.07, 133.75, 128.26, 127.98, 123.96, 123.20, 121.21, 110.30, 77.41, 77.16, 76.91, 21.25, 21.22. The characterization matches that previously reported.[16]

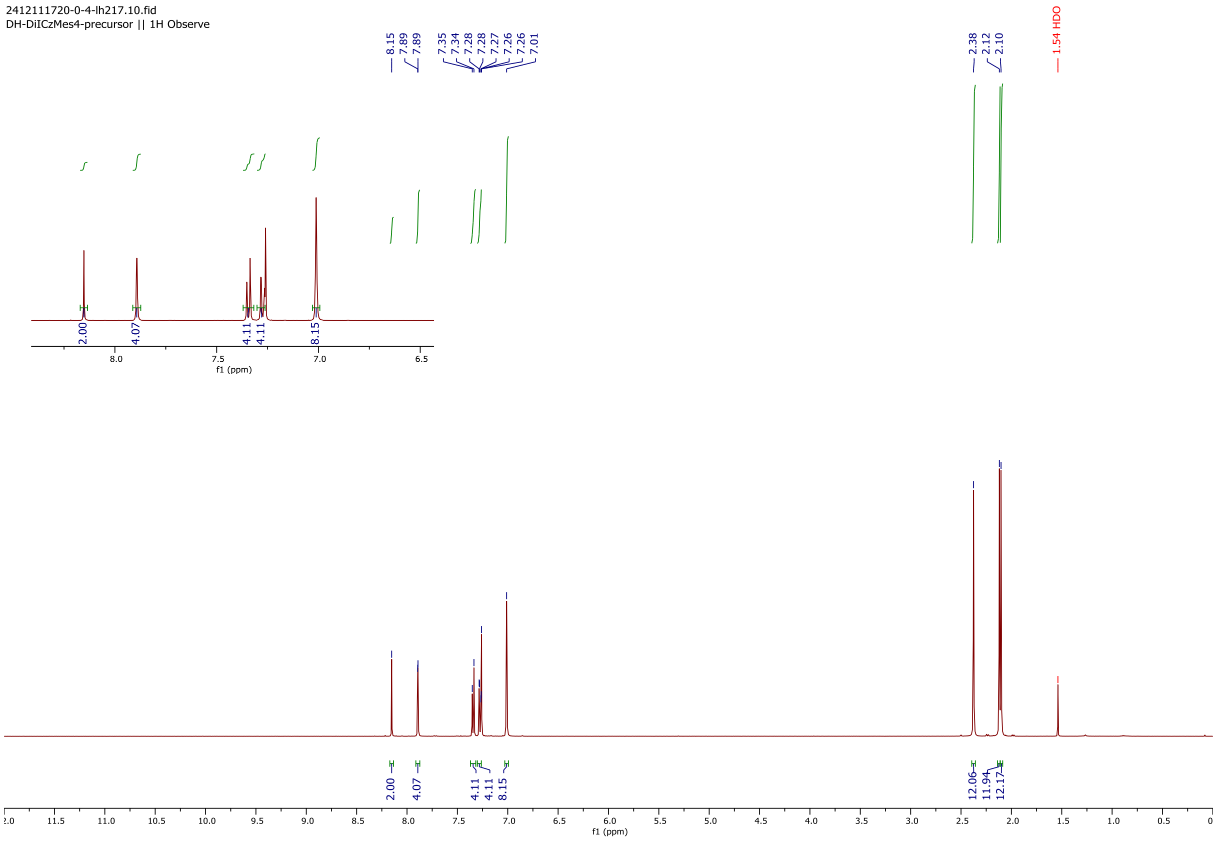

Figure **S15**. ^{1}H NMR spectrum of 9,9'-(2,5-dibromo-1,4-phenylene)bis(3,6-dimesityl-9*H*-carbazole).

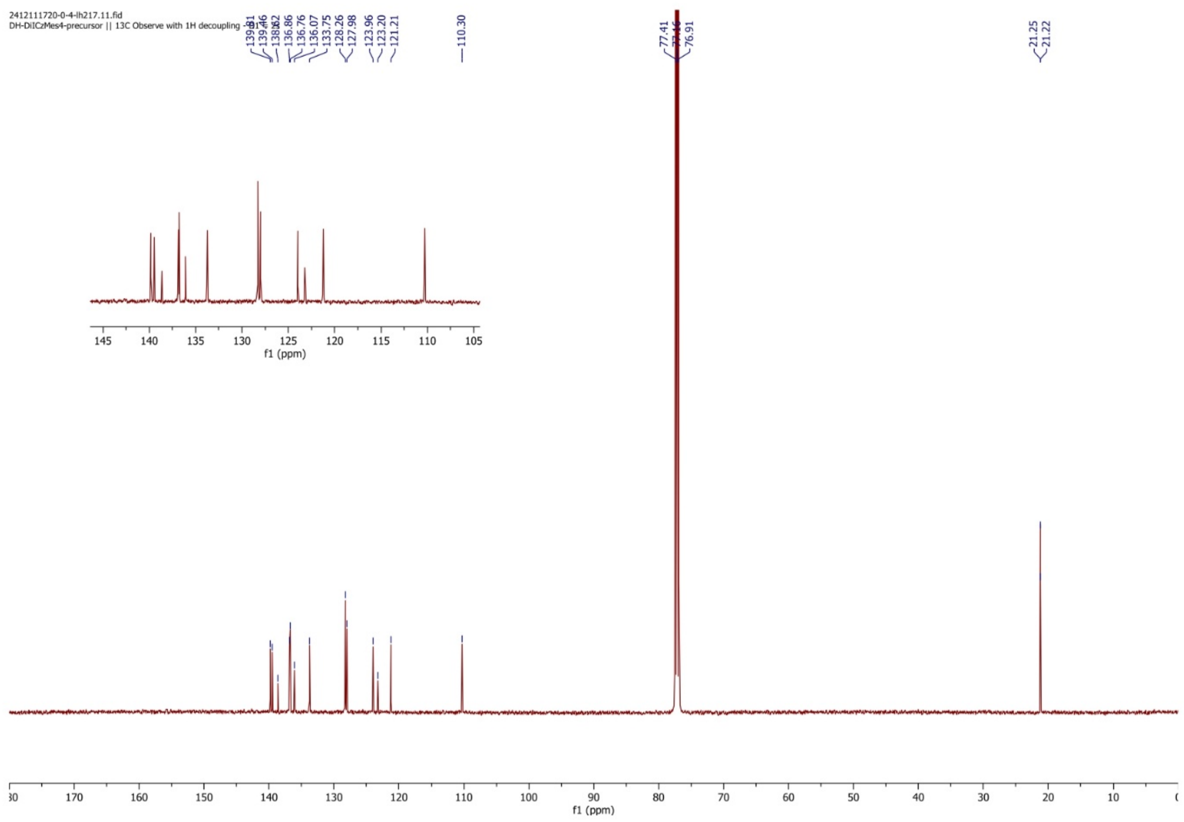

Figure **S16**. ^{13}C NMR spectrum of 9,9'-(2,5-dibromo-1,4-phenylene)bis(3,6-dimesityl-9*H*-carbazole).

DiICzMes$_4$

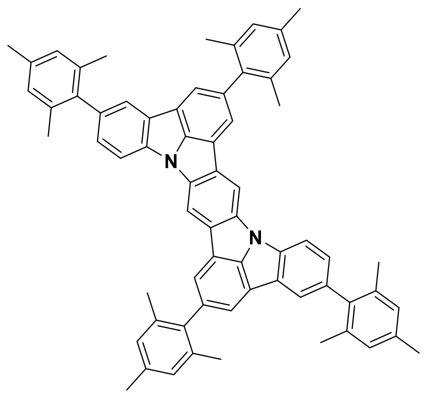

Reaction with 9,9'-(2,5-dibromo-1,4-phenylene)bis(3,6-dimesityl-9*H*-carbazole) (1.35 g, 1.30 mmol, 1.0 equiv.) using General Procedure 2. The crude residue was purified via column chromatography (DCM:Petroleum ether 15:85). The resulting solid was precipitated from a saturated solution of toluene by slow addition of methanol. The mixture was filtered to afford the product as a yellow solid. **Yield:** 52% (0.61 g). **R$_f$:** 0.32 (DCM:Petroleum ether 15:85). **Mp:** Decomposed at 387 °C (lit. Mp: Decomposed 392 °C).[15] **GPC-HPLC:** 99% pure (100% THF with a retention time of 10.5 min). **^{1}H NMR (500 MHz, CDCl$_3$) δ (ppm):** 8.68 (s, 2H), 8.15 (d, *J* = 8.2 Hz, 2H), 8.01 (s, 2H), 7.93 (d, *J* = 1.6 Hz, 2H), 7.84 (s, 2H), 7.43 (dd, *J* = 8.2, 1.6 Hz, 2H), 7.07 (s, 4H), 7.03 (s, 4H), 2.43 (s, 6H), 2.39 (s, 6H), 2.13 (s, 24H). **^{13}C NMR (126 MHz, CDCl$_3$) δ (ppm):** 144.33, 140.44, 139.23, 137.97, 136.96, 136.87, 136.74, 136.67, 136.33, 135.28, 134.76, 130.19, 129.65, 128.33, 128.30, 128.27, 124.15, 120.94, 120.51, 118.98, 118.79, 112.20, 106.77, 21.41, 21.28, 21.23, 21.14. The characterization matches that previously reported.[15]

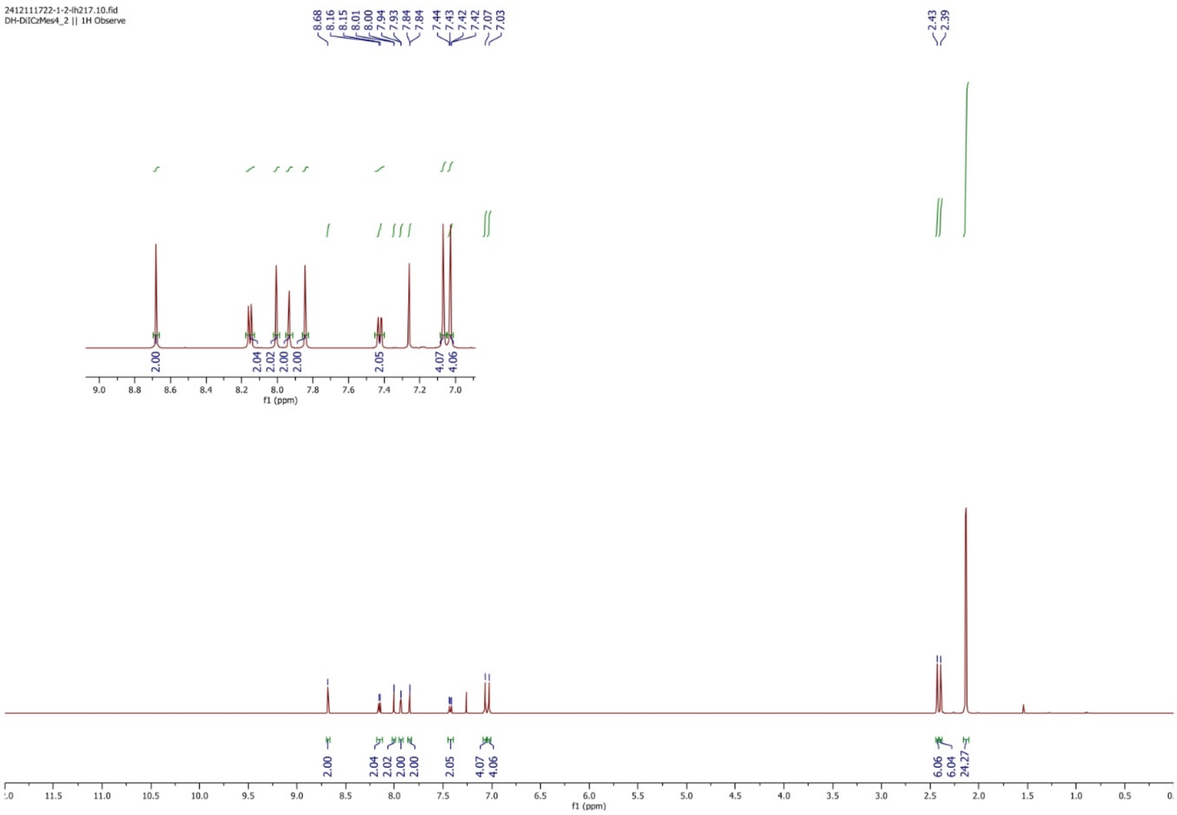

Figure **S17**. ^{1}H NMR spectrum of **DiICzMes$_4$** in $CDCl_3$.

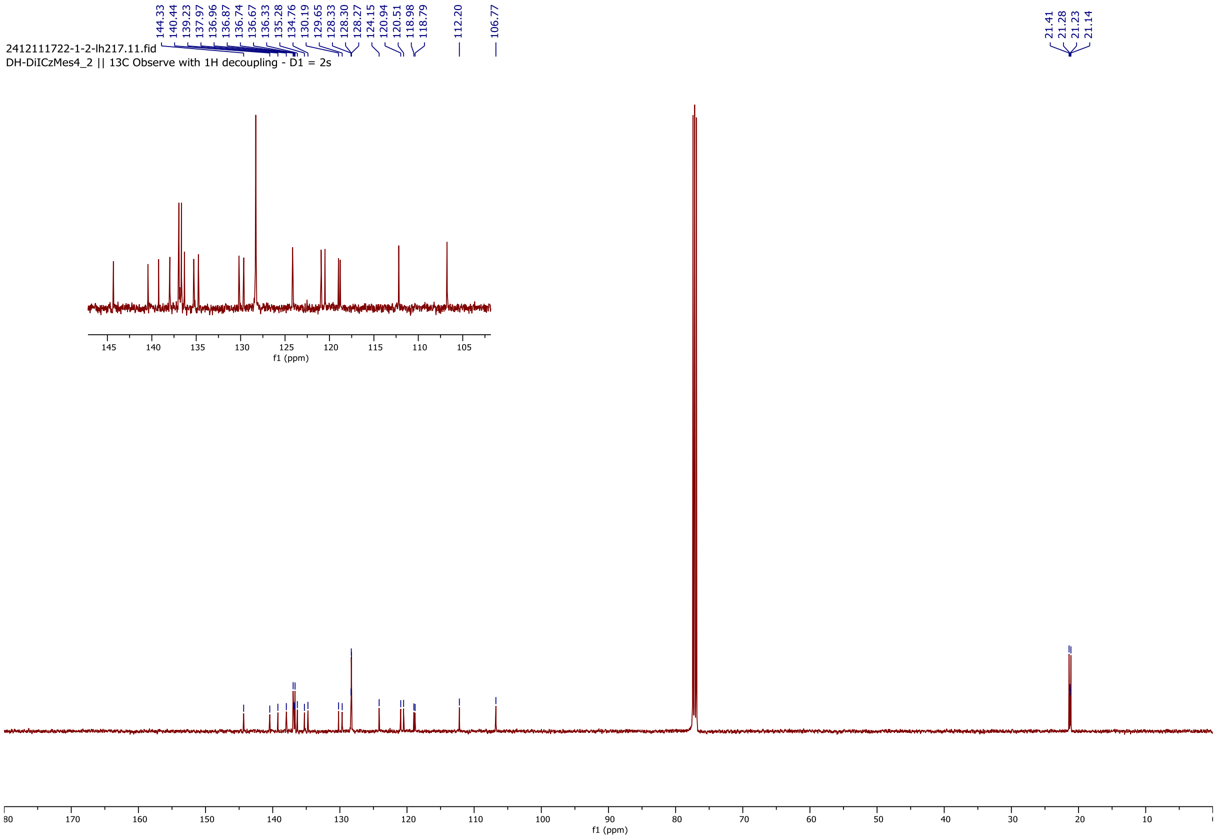

Figure **S18**. ^{13}C NMR spectrum of **DiICzMes$_4$** in $CDCl_3$.

25/09/2024 18:20:18 Page 1 / 1

HPLC Trace Report25Sep2024

<Sample Information>

Sample Name	: diiczmes4-lea		
Sample ID	:		
Method Filename	: 100% THF 20 mins 280nm - DH.lcm		
Batch Filename	: diicz.lcb		
Vial #	: 1-14	Sample Type	: Unknown
Injection Volume	: 5 uL		
Date Acquired	: 05/10/2022 11:56:21	Acquired by	: System Administrator
Date Processed	: 05/10/2022 12:16:22	Processed by	: System Administrator

<Chromatogram>

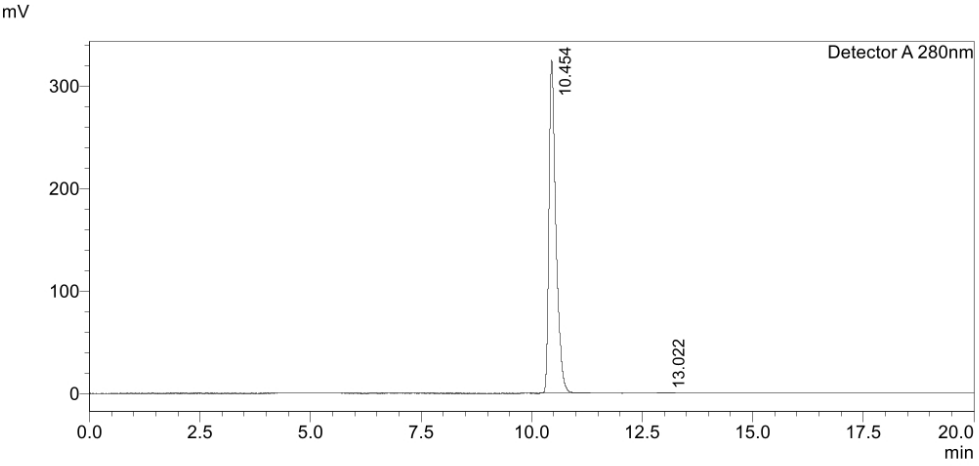

<Peak Table>

Detector A 280nm

Peak#	Ret. Time	Area	Height	Area%	Area/Height	Width at 5% Height
1	10.454	3561331	324555	99.933	10.973	0.394
2	13.022	2371	225	0.067	10.557	0.299
Total		3563701	324780	100.000		

Figure **S19**. GPC-HPLC trace of **DiICzMes$_4$**.

General Procedure 3: SNAr coupling to 1,4-dibromo-2,5-difluorobenzene using Cs_2CO_3

To an oven-dried Schlenk tube were added Cs_2CO_3 (4.0 equiv.), the corresponding amine (2.5 equiv.) and DMF to reach a concentration of 0.08 M. The mixture was backfilled with nitrogen and the mixture stirred for 15 min. 1,4-dibromo-2,5-difluorobenzene (1.0 equiv.) was then added and the reaction mixture heated at 120 °C for 24 h under a nitrogen atmosphere. The reaction was then allowed to cool to room temperature before water (75 mL) was added. The reaction mixture extracted with DCM (3 × 100 mL). The combined organic layers were dried over Na_2SO_4, filtered and the solvent removed under reduced pressure to afford the crude product.

9',9''''-(2,5-dibromo-1,4-phenylene)bis(3,3'',6,6''-tetra-*tert*-butyl-9'H-9,3':6',9''-tercarbazole)

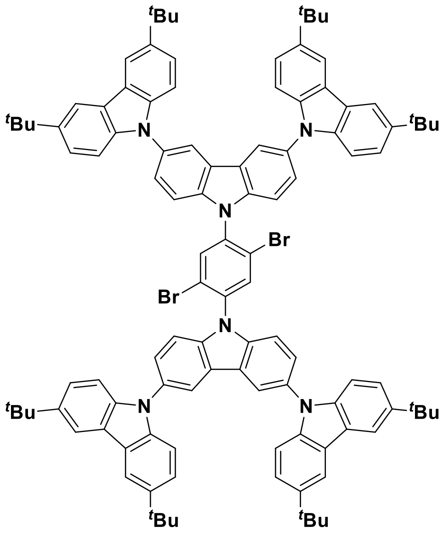

Reaction with 3,3'',6,6''-tetra-*tert*-butyl-9'H-9,3':6',9''-tercarbazole (1.99 g, 2.76 mmol, 2.5 equiv.) using General Procedure 3. The crude product was purified via flash column chromatography (0 – 30% DCM in Hexane) and the product obtained as a white solid. **Yield:** 91% (1.69 g). **R_f :** 0.27 (DCM:hexane 0.15:0.85) **Mp:** Decomposed at 350°C. **GPC-HPLC:** 100% pure (100% THF with 6.24 min retention time). **^{1}H NMR (500 MHz, $CDCl_3$) δ (ppm):** 8.31 (s, 6H), 8.18 (d, J = 1.7 Hz, 8H), 7.73 (dd, J = 8.6, 2.0 Hz, 4H), 7.56 (d, J = 8.6 Hz, 4H), 7.49 (dd, J = 8.7, 1.9 Hz, 8H), 7.41 (d, J = 8.6 Hz, 8H), 1.48 (s, 72H). **^{13}C NMR (126 MHz, $CDCl_3$) δ (ppm):** 142.87, 140.15, 140.03, 138.53, 136.34, 131.92, 126.43, 124.57, 123.77, 123.55, 123.34, 119.72, 116.44, 111.61, 109.22, 77.41, 77.16, 76.91, 34.90, 32.19. **MALDI-TOF HRMS $[M]^+$** Calculated: ($C_{110}H_{110}Br_2N_6$) 1674.71565; Found: 1674.71622.

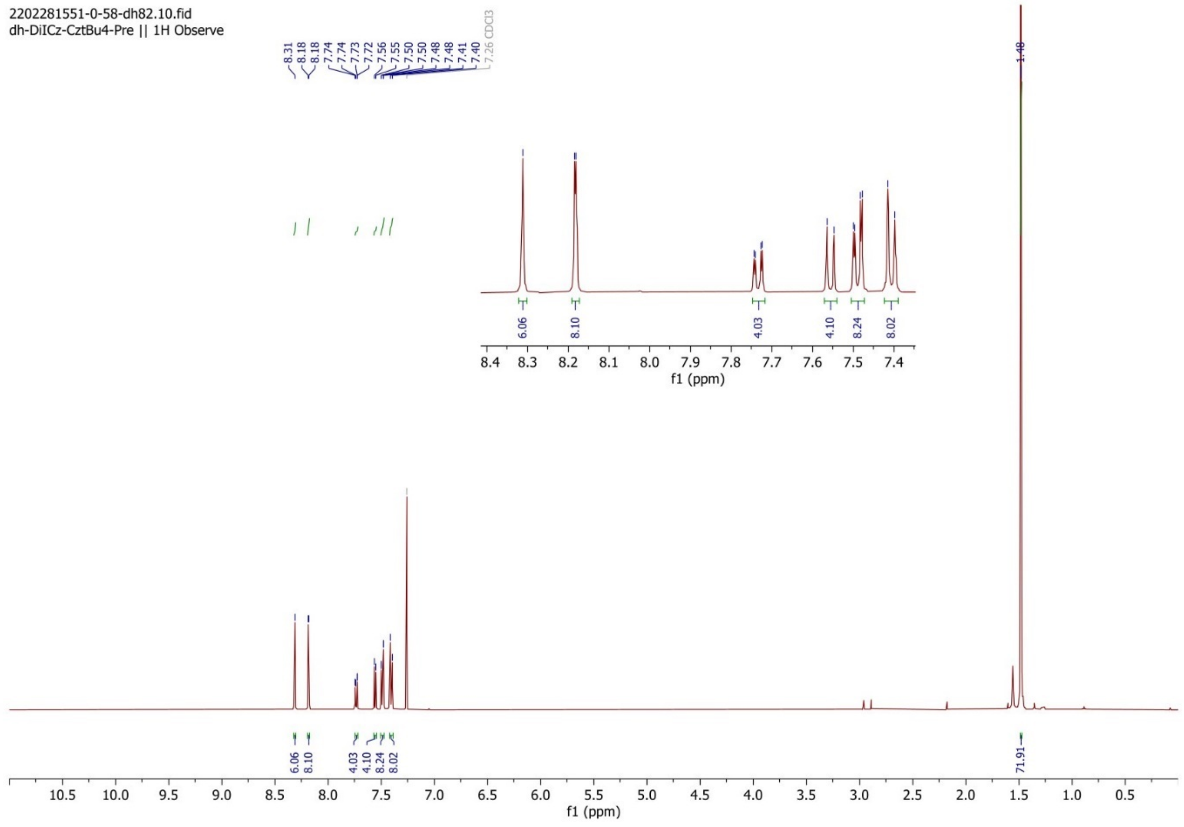

Figure **S20**. ^{1}H NMR spectrum of 9',9''''-(2,5-dibromo-1,4-phenylene)bis(3,3'',6,6''-tetra-*tert*-butyl-9'H-9,3':6',9''-tercarbazole) in $CDCl_3$.

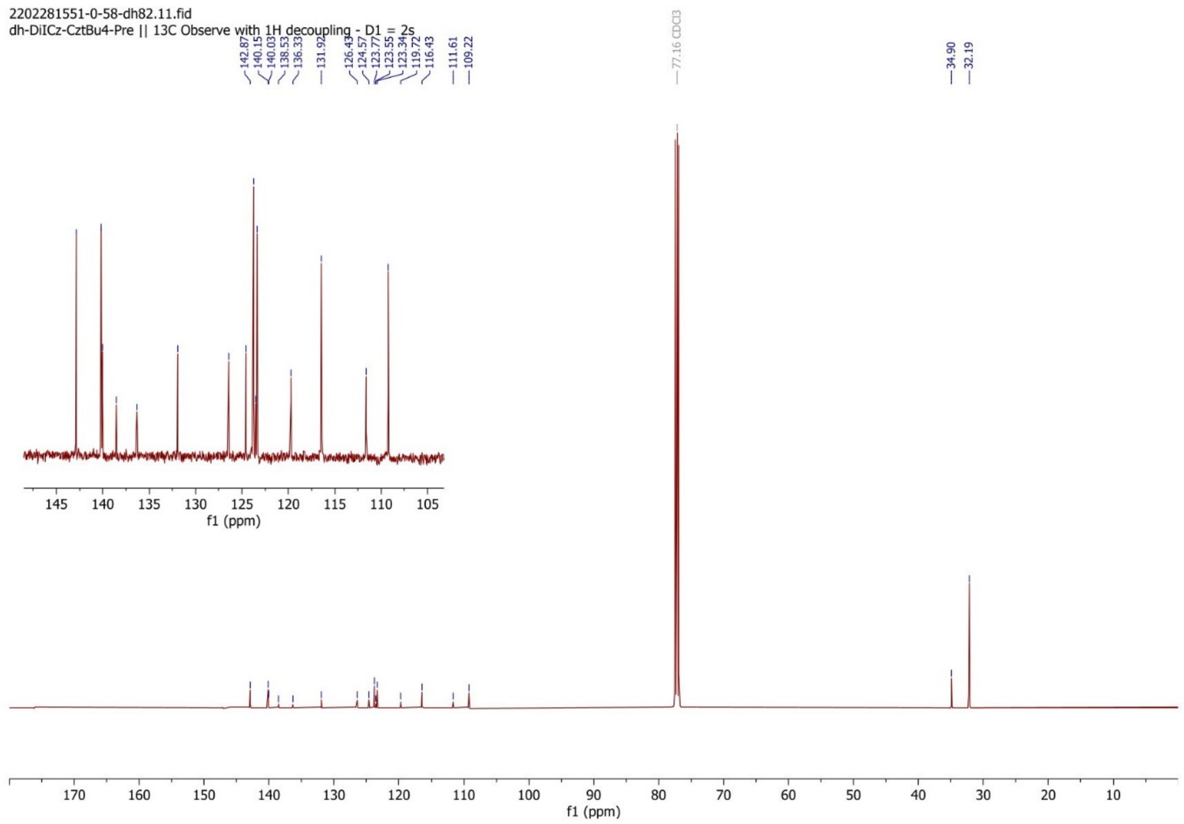

Figure **S21**. ^{13}C NMR spectrum of 9',9''''-(2,5-dibromo-1,4-phenylene)bis(3,3'',6,6''-tetra-*tert*-butyl-9'H-9,3':6',9''-tercarbazole) in $CDCl_3$.

HPLC Trace Report07Dec2024

<Sample Information>

Sample Name	: dh-diicztbucz-precursor-2		
Sample ID	:		
Method Filename	: 100% THF 20 mins 280nm - new-please use.lcm		
Batch Filename	: DH.lcb		
Vial #	: 2-6	Sample Type	: Unknown
Injection Volume	: 10 uL		
Date Acquired	: 07/12/2024 16:50:54	Acquired by	: System Administrator
Date Processed	: 07/12/2024 17:10:56	Processed by	: System Administrator

<Chromatogram>

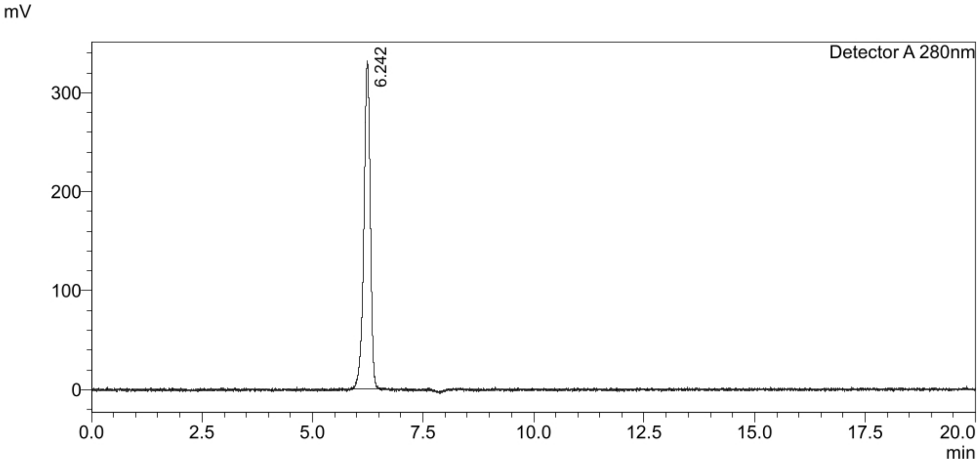

<Peak Table>

Detector A 280nm

Peak#	Ret. Time	Area	Height	Area%	Area/Height	Width at 5% Height
1	6.242	3350788	330201	100.000	10.148	0.360
Total		3350788	330201	100.000		

Figure **S22**. GPC-HPLC trace of 9',9''''-(2,5-dibromo-1,4-phenylene)bis(3,3'',6,6''-tetra-*tert*-butyl-9'H-9,3':6',9''-tercarbazole).

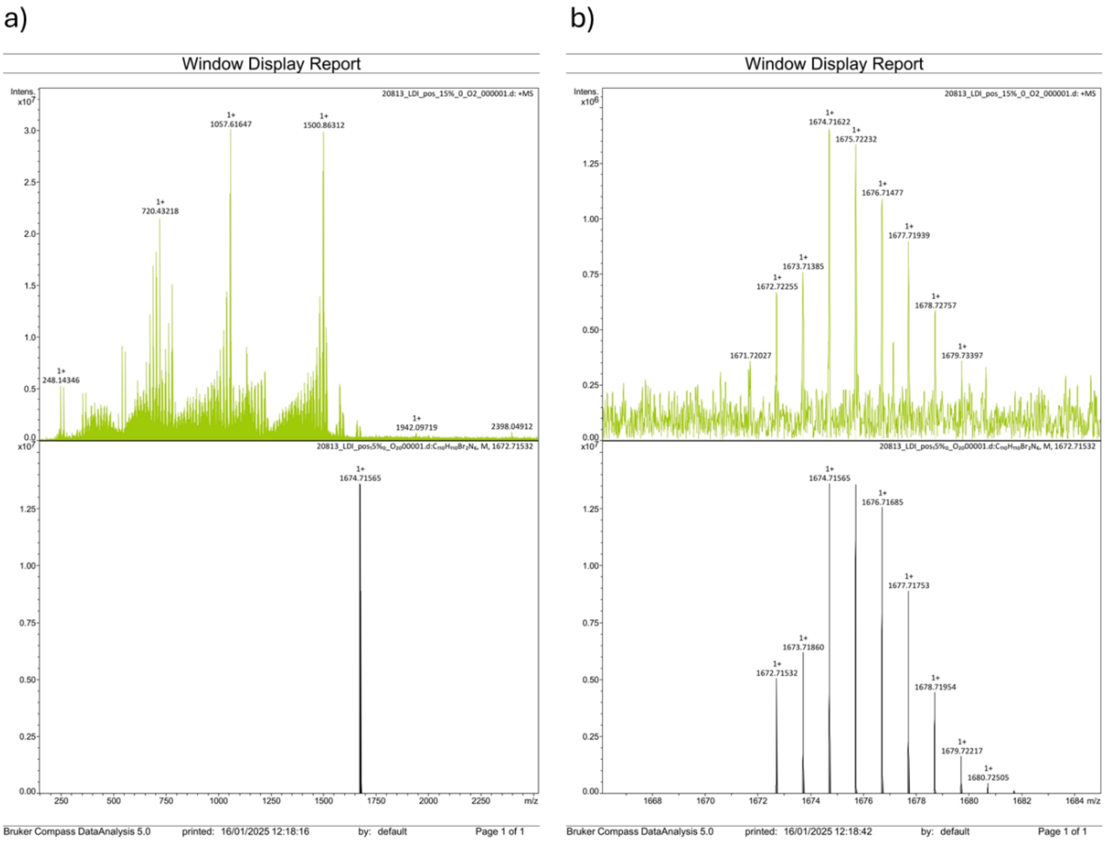

Figure **S23**. MALDI-TOF HRMS of 9',9''''-(2,5-dibromo-1,4-phenylene)bis(3,3'',6,6''-tetra-*tert*-butyl-9'H-9,3':6',9''-tercarbazole. a) Full spectrum and b) zoomed in spectrum.

DiICztBuCz$_4$

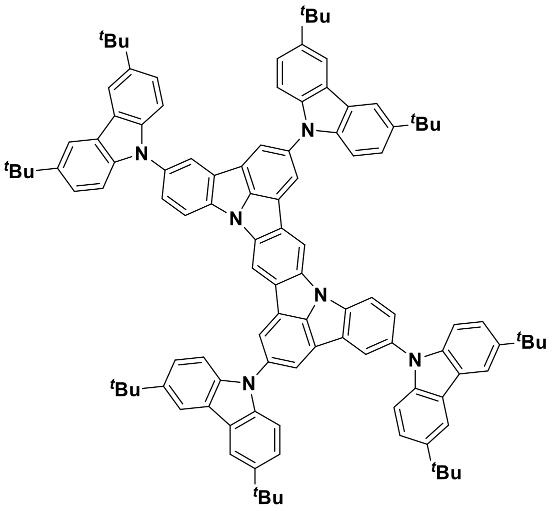

Reaction with 9',9''''-(2,5-dibromo 1,4-phenylene)bis(3,3'',6,6''-tetra-*tert*-butyl-9'H-9,3':6',9''-tercarbazole) using General Procedure 2. The crude residue was purified via column chromatography (DCM:petroleum ether 15:85). The resulting solid was precipitated from a saturated solution of toluene by slow addition of methanol. The mixture was filtered to afford the product as a yellow solid. **Yield:** 82% (0.74 g). **R$_f$:** 0.25 (DCM:petroleum ether 15:85). **Mp:** 378 – 383 °C. **GPC-HPLC:** 96% pure (100% THF with 9.65 min retention time).

^{1}H NMR (500 MHz, CDCl$_3$) δ (ppm): 8.73 (2, 1H), 8.44 (s, 2H), 8.33 (d, *J* = 1.8 Hz, 2H), 8.29 – 8.22 (m, 8H), 8.22 – 8.16 (m, 4H), 7.84 (dd, *J* = 8.5, 1.9 Hz, 2H), 7.52 – 7.45 (m, 8H), 7.45 – 7.40 (m, 8H), 1.50 (s, 36H), 1.48 (s, 36H). **^{13}C NMR (126 MHz, CDCl$_3$) δ (ppm):** 144.32, 143.04, 142.98, 140.83, 139.97, 138.05, 135.61, 134.09, 132.74, 130.95, 129.86, 126.74, 123.88, 123.47, 123.38, 122.49, 120.46, 120.09, 119.61, 119.10, 116.47, 113.19, 109.24, 109.19, 107.29, 34.94, 34.91, 32.22, 32.18.

MALDI-TOF HRMS [M]$^+$ Calculated: ($C_{110}H_{108}N_6$) 1513.86627; Found: 1513.87312.

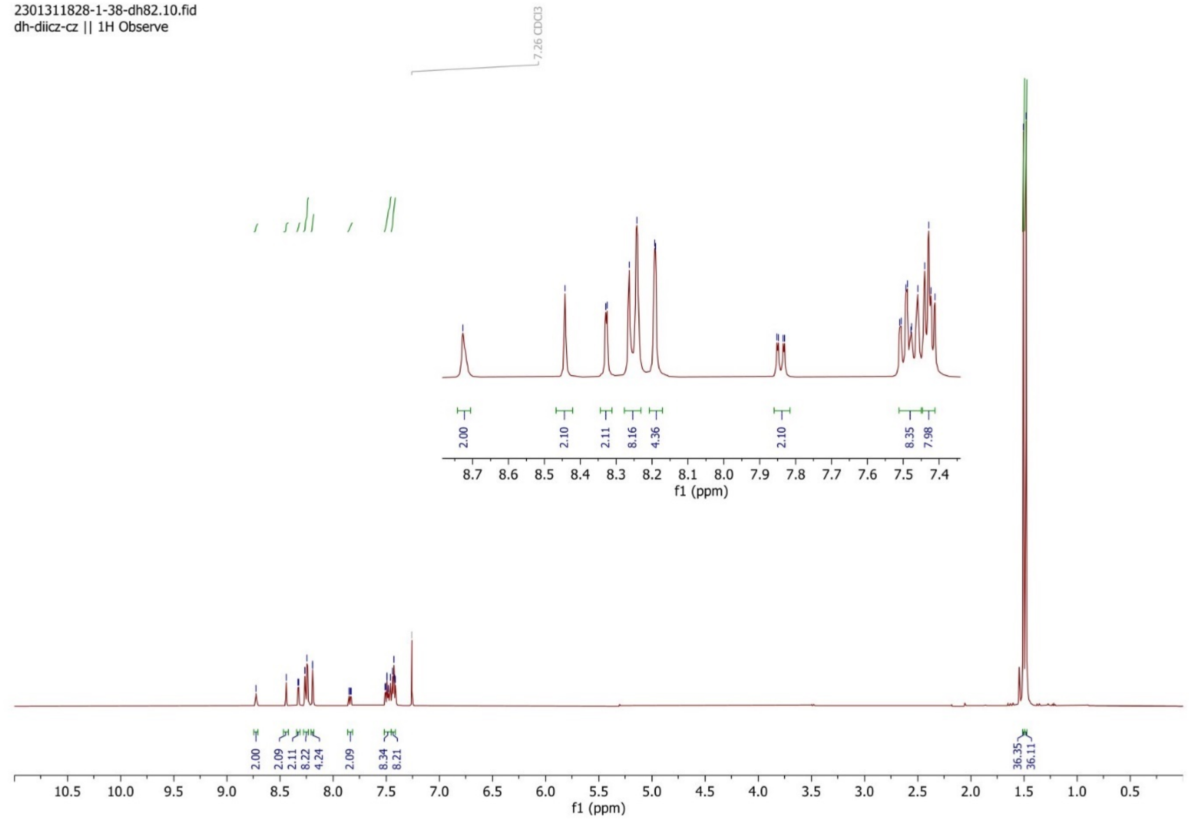

Figure **S24**. ^{1}H NMR spectrum of **DiICztBuCz$_4$** in CDCl$_3$.

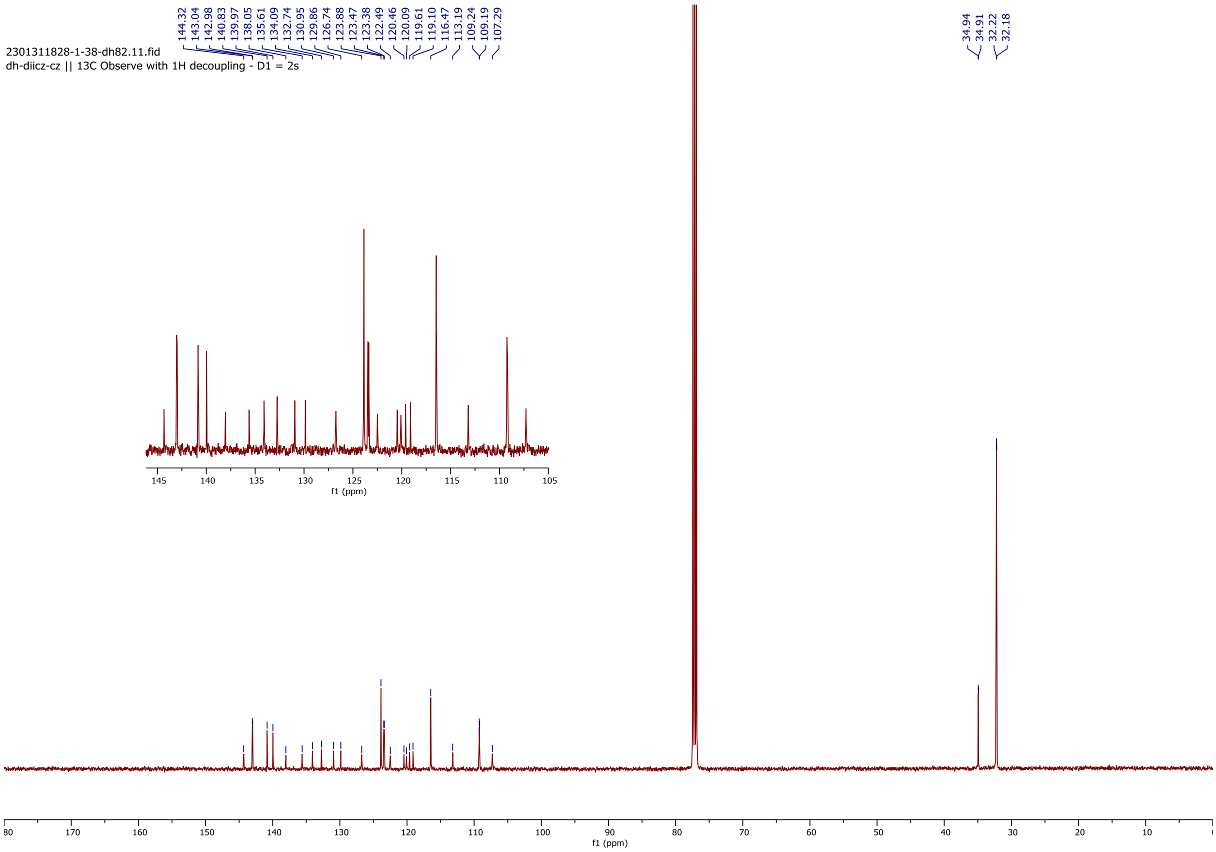

Figure **S25**. ^{13}C NMR spectrum of **DiICztBuCz$_4$** in $CDCl_3$.

HPLC Trace Report12Feb2022

<Sample Information>

Sample Name	: dh-diicz-pure		
Sample ID	:		
Method Filename	: 100% THF 20 mins 280nm - DH.lcm		
Batch Filename	: DH-11022022 - try 2.lcb		
Vial #	: 1-9	Sample Type	: Unknown
Injection Volume	: 5 uL		
Date Acquired	: 11/02/2022 19:48:48	Acquired by	: System Administrator
Date Processed	: 11/02/2022 20:08:50	Processed by	: System Administrator

<Chromatogram>

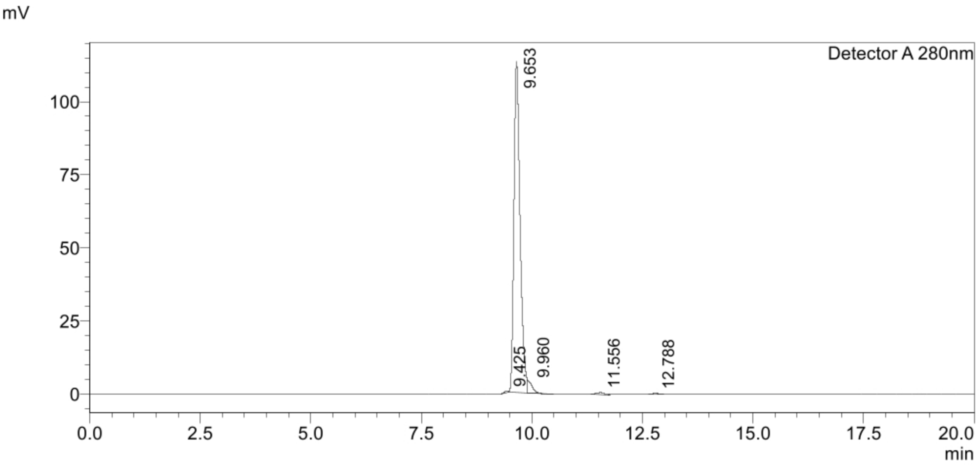

<Peak Table>

Detector A 280nm

Peak#	Ret. Time	Area	Height	Area%	Area/Height	Width at 5% Height
1	9.425	3113	525	0.273	5.930	--
2	9.653	1096838	113020	96.231	9.705	0.352
3	9.960	31200	3681	2.737	8.475	--
4	11.556	6926	617	0.608	11.217	0.345
5	12.788	1722	174	0.151	9.914	0.288
Total		1139800	118017	100.000		

Figure **S26**. GOC-HPLC trace of **DiICztBuCz$_4$**.

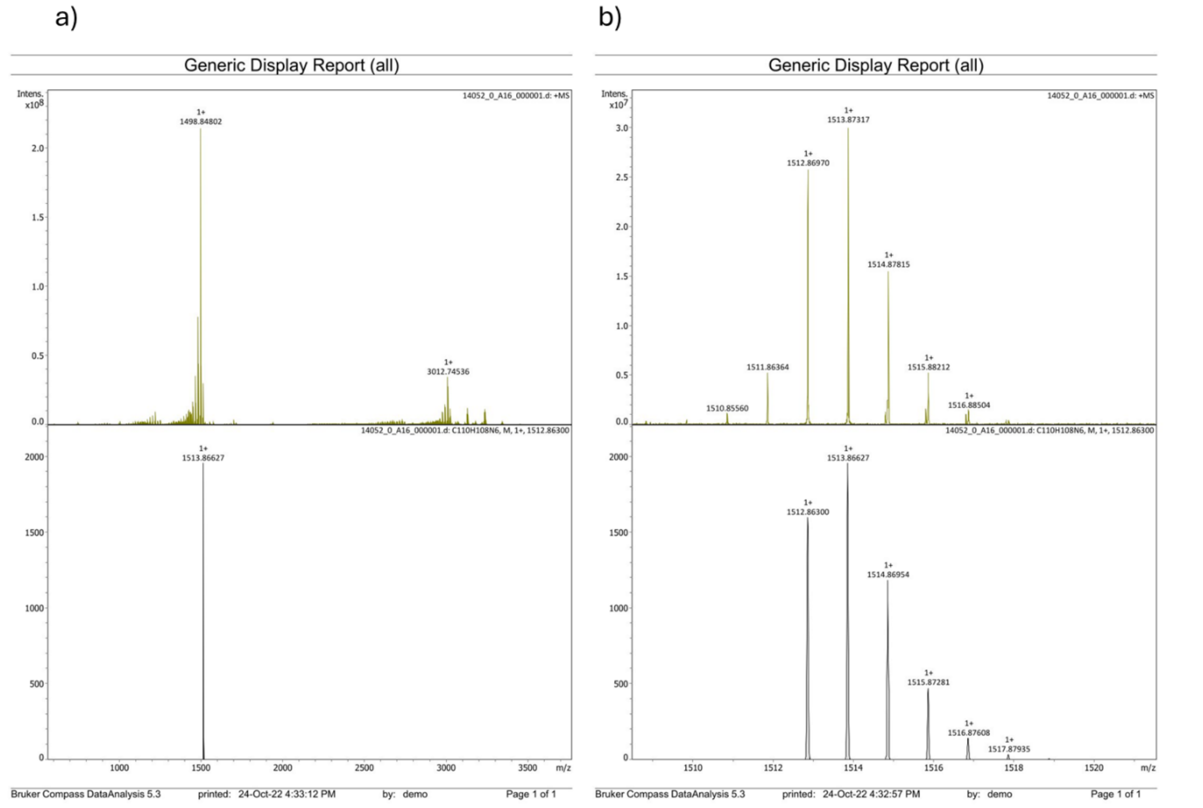

Figure **S27**. MALDI-TOF HRMS of **DiICztBuCz$_4$**. a) Full spectrum and b) zoomed in spectrum.

9,9'-(2,5-dibromo-1,4-phenylene)bis(N^3,N^3,N^6,N^6-tetrakis(4-(*tert*-butyl)phenyl)-9*H*-carbazole-3,6-diamine)

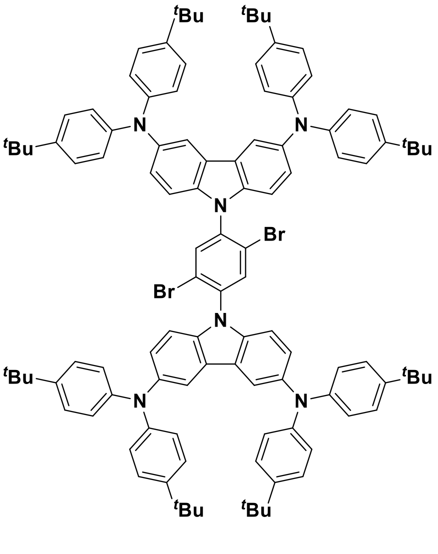

Reaction with N^3,N^3,N^6,N^6-tetrakis(4-(*tert*-butyl)phenyl)-9*H*-carbazole-3,6-diamine (1.74 g, 2.39 mmol, 2.5 equiv.) using General Procedure 3. The crude product was washed with methanol (50 mL) and filtered to obtain the product as a white solid. **Yield:** 87 % (1.40 g). **Mp:** Decomposed at 278 °C. **GPC-HPLC:** 97% pure (100% THF with 12.94 min retention time). **^{1}H NMR (500 MHz, CDCl$_3$) δ (ppm):** 7.97 (s, 2H), 7.78 (s, 4H), 7.25 – 7.20 (m, J = 8.6 Hz, 20H), 7.07 (d, J = 8.8 Hz, 4H), 7.02 (d, J = 8.3 Hz, 16H), 1.30 (s, 72H). **^{1}H NMR (400 MHz, CD$_2$Cl$_2$) δ (ppm):** 8.03 (s, 2H), 7.77 (d, J = 2.1 Hz, 4H), 7.31 – 7.17 (m, 20H), 7.15 (d, J = 8.7 Hz, 4H), 6.98 (d, J = 8.7 Hz, 16H), 1.28 (s, 72H). ^{1}H NMR in CD$_2$Cl$_2$ is included for a clearer analysis of coupling pattern. **^{13}C NMR (126 MHz, CDCl$_3$) δ (ppm):** 146.03, 144.51, 141.75, 137.92, 135.92, 129.19, 128.38, 126.03, 125.94, 125.45, 124.57, 122.44, 118.53, 111.05, 34.32, 31.61. **MALDI-TOF HRMS [M]$^+$** Calculated: ($C_{110}H_{118}Br_2N_6$) 1682.77825; Found: 1682.77763.

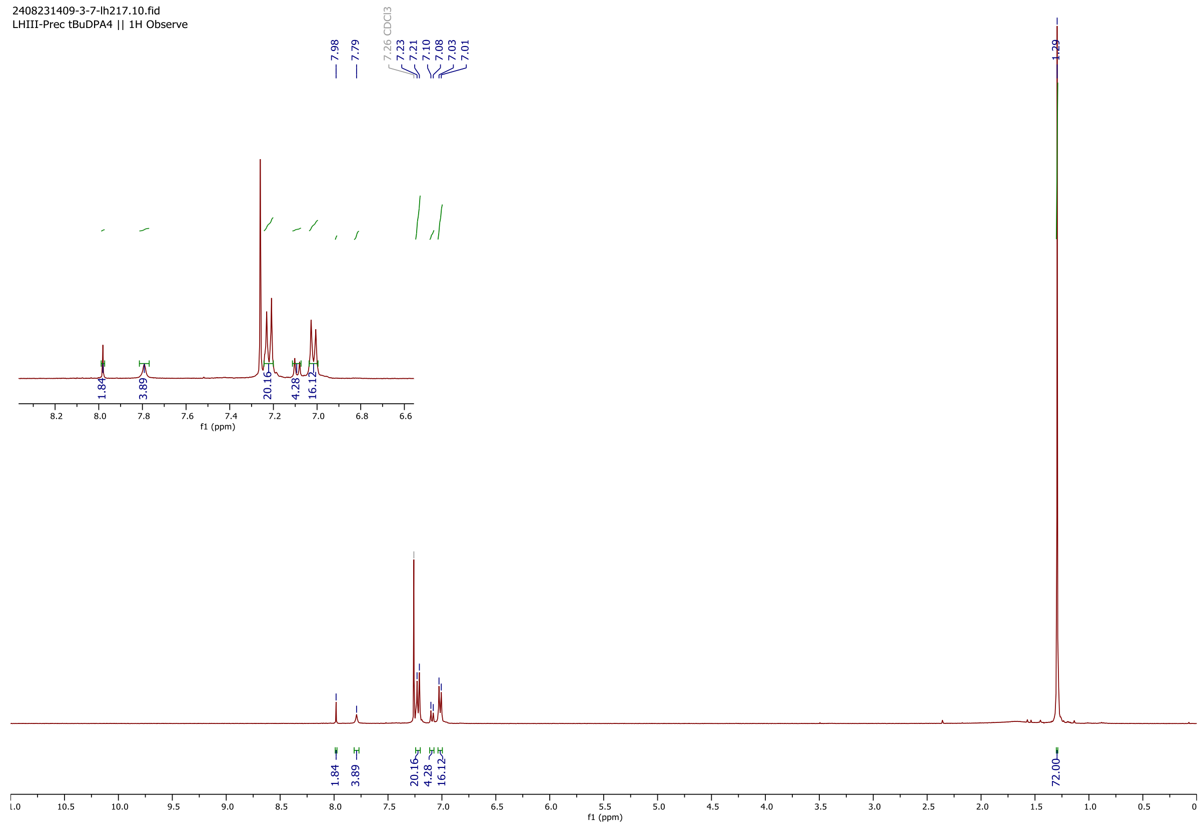

Figure **S28**. ^{1}H NMR spectrum of 9,9'-(2,5-dibromo-1,4-phenylene)bis(N^3,N^3,N^6,N^6-tetrakis(4-(*tert*-butyl)phenyl)-9*H*-carbazole-3,6-diamine) in $CDCl_3$.

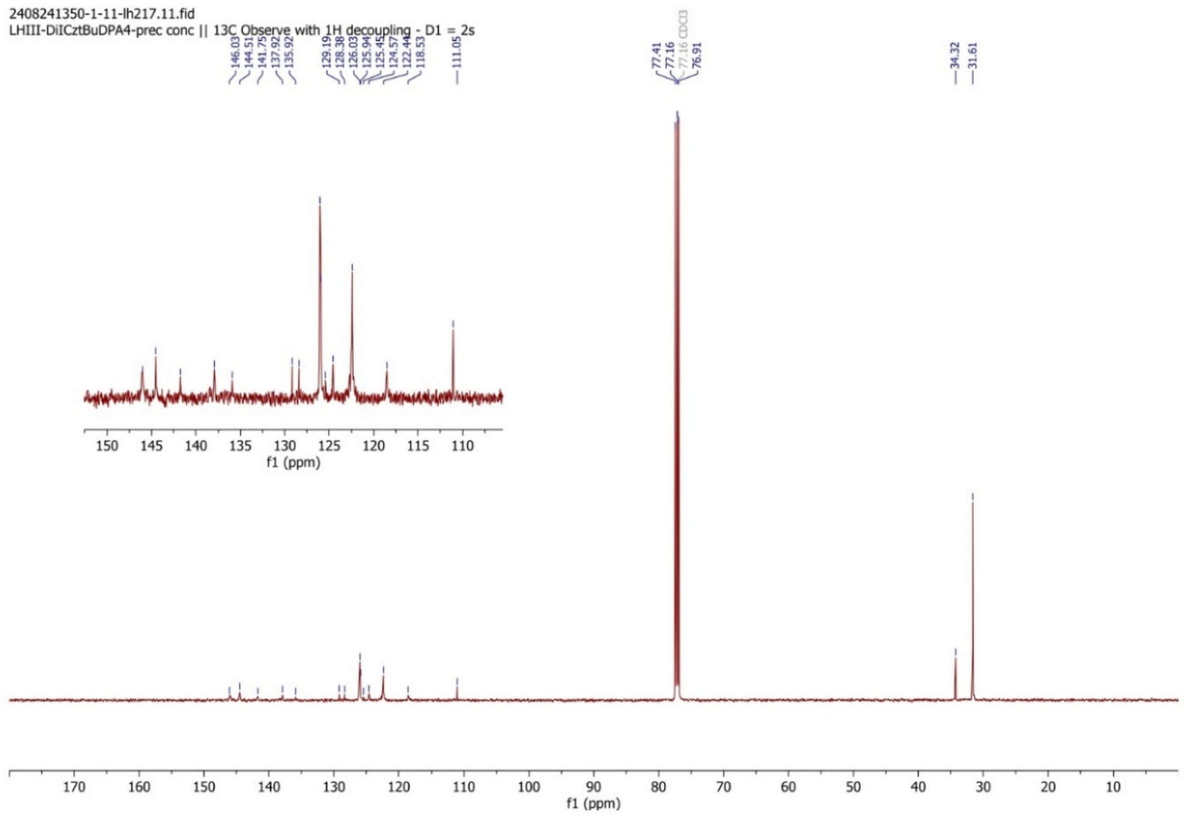

Figure **S29**. ^{13}C NMR spectrum of 9,9'-(2,5-dibromo-1,4-phenylene)bis(N^3,N^3,N^6,N^6-tetrakis(4-(*tert*-butyl)phenyl)-9*H*-carbazole-3,6-diamine) in $CDCl_3$.

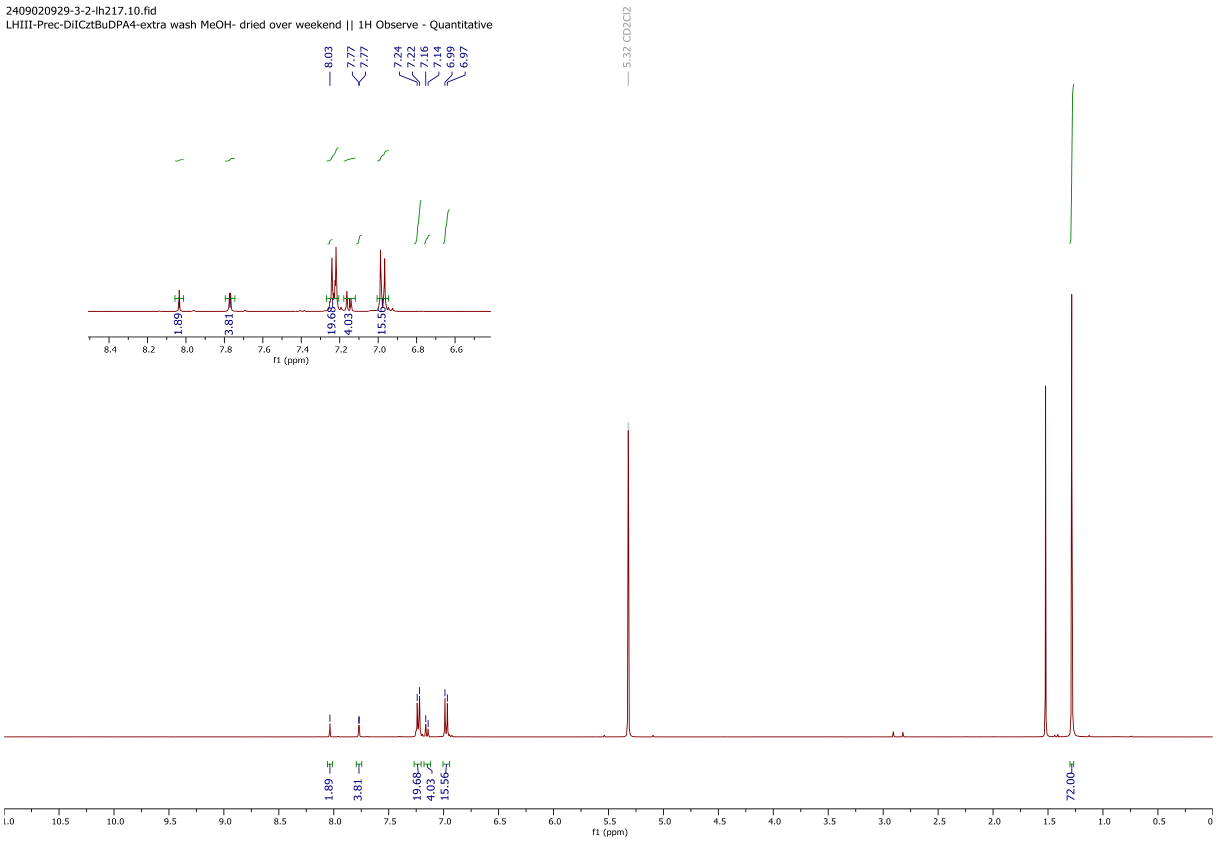

Figure **S30**. ^{1}H NMR spectrum of 9,9'-(2,5-dibromo-1,4-phenylene)bis(N3,N3,N6,N6-tetrakis(4-(*tert*-butyl)phenyl)-9*H*-carbazole-3,6-diamine) in CD_2Cl_2.

HPLC Trace Report05Jul2022

<Sample Information>

Sample Name	: eb-diicz-dpa-pre		
Sample ID	:		
Method Filename	: 100% THF 20 mins 280nm - new-please use.lcm		
Batch Filename	: DiICz-dev-05-07-2022.lcb		
Vial #	: 1-9	Sample Type	: Unknown
Injection Volume	: 5 uL		
Date Acquired	: 05/07/2022 10:12:21	Acquired by	: System Administrator
Date Processed	: 05/07/2022 10:32:23	Processed by	: System Administrator

<Chromatogram>

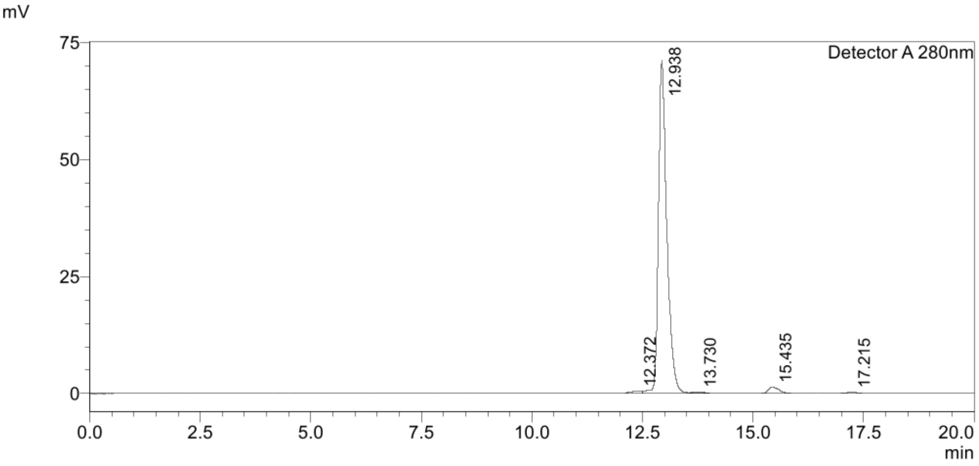

<Peak Table>

Detector A 280nm

Peak#	Ret. Time	Area	Height	Area%	Area/Height	Width at 5% Height
1	12.372	5543	408	0.577	13.600	--
2	12.938	929118	70913	96.777	13.102	0.471
3	13.730	2415	218	0.252	11.081	0.337
4	15.435	20685	1285	2.155	16.091	0.502
5	17.215	2295	165	0.239	13.891	0.401
Total		960056	72989	100.000		

Figure **S31**. GPC-HPLC trace of 9,9'-(2,5-dibromo-1,4-phenylene)bis(N^3,N^3,N^6,N^6-tetrakis(4-(*tert*-butyl)phenyl)-9*H*-carbazole-3,6-diamine).

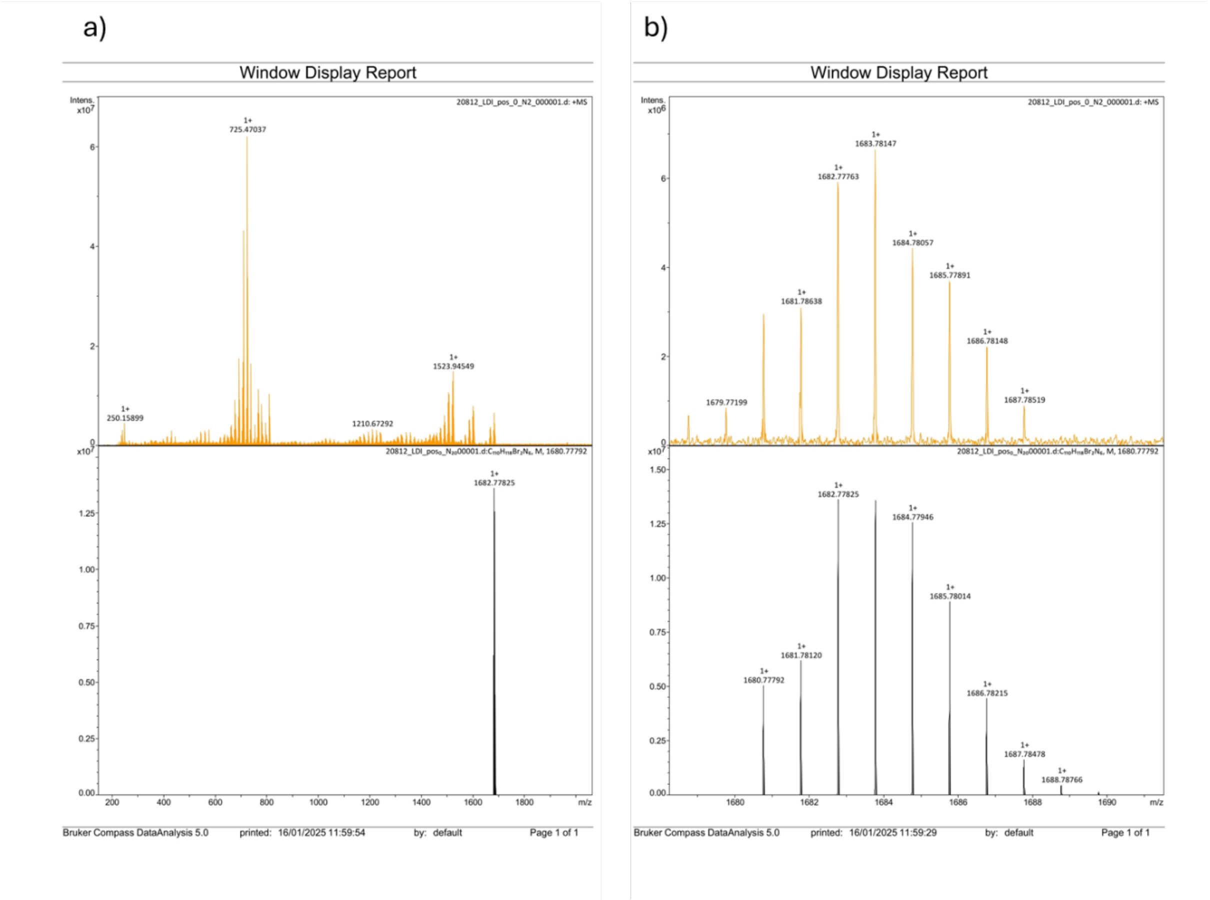

Figure **S32**. MALDI-TOF HRMS of 9,9'-(2,5-dibromo-1,4-phenylene)bis(N^3,N^3,N^6,N^6-tetrakis(4-(*tert*-butyl)phenyl)-9*H*-carbazole-3,6-diamine). a) Full spectrum and b) zoomed in spectrum.

DiICztBuDPA$_4$

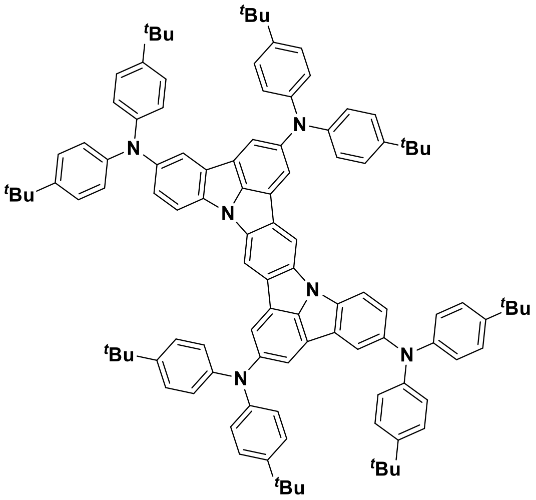

Reaction with 9,9'-(2,5-dibromo-1,4-phenylene) bis(N^3,N^3,N^6,N^6-tetrakis(4-(*tert*-butyl)phenyl)-9*H*-carbazole-3,6-diamine) (1.00 g, 0.59 mmol, 1.0 equiv.) using General Procedure 2. The crude residue was purified by column chromatography (DCM:hexane 0 – 30%). The resulting solid was precipitated from a saturated solution of toluene by slow addition of methanol. The mixture was filtered to afford the product as a yellow solid. **Yield:** 42% (0.16 g). **R_f :** 0.27 (DCM:Hexane 15:85). **Mp:** Decomposed**:** 358 °C. **GPC-HPLC:** 97% pure (100% THF with 9.808 min retention time).

^{1}H NMR (400 MHz, CD_2Cl_2) δ (ppm): 8.53 (s, 2H), 8.05 (s, 2H), 7.89 (d, *J* = 8.6 Hz, 2H), 7.80 (d, *J* = 9.8 Hz, 4H), 7.36 (dd, *J* = 8.6, 2.2 Hz, 2H), 7.26 (dd, *J* = 8.6, 5.8 Hz, 16H), 7.10 – 6.92 (m, 16H), 1.30 (d, *J* = 1.9 Hz, 72H). **^{13}C NMR (126 MHz, CD_2Cl_2) δ (ppm):** 147.28, 146.35, 145.59, 144.91, 144.79, 143.30, 135.96, 135.53, 131.24, 129.66, 126.62, 126.50, 125.53, 123.38, 122.66, 120.89, 120.84, 120.37, 119.81, 119.53, 113.10, 107.15, 34.68, 34.63, 31.75, 31.73.

MALDI-TOF HRMS [M]$^+$ Calculated: ($C_{110}H_{116}N_6$) 1521.92887; Found: 1521.93930

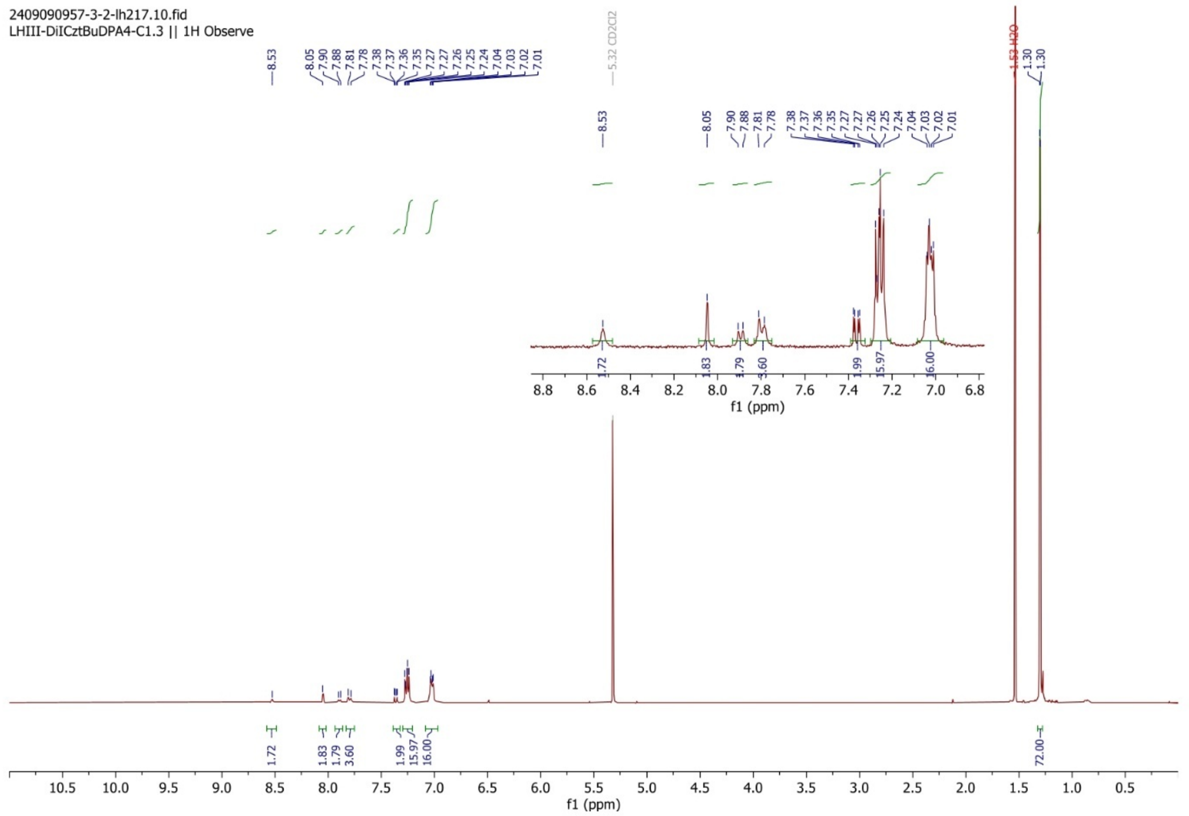

Figure **S33**. ^{1}H NMR spectrum of **DiICztBuDPA$_4$** in CD_2Cl_2.

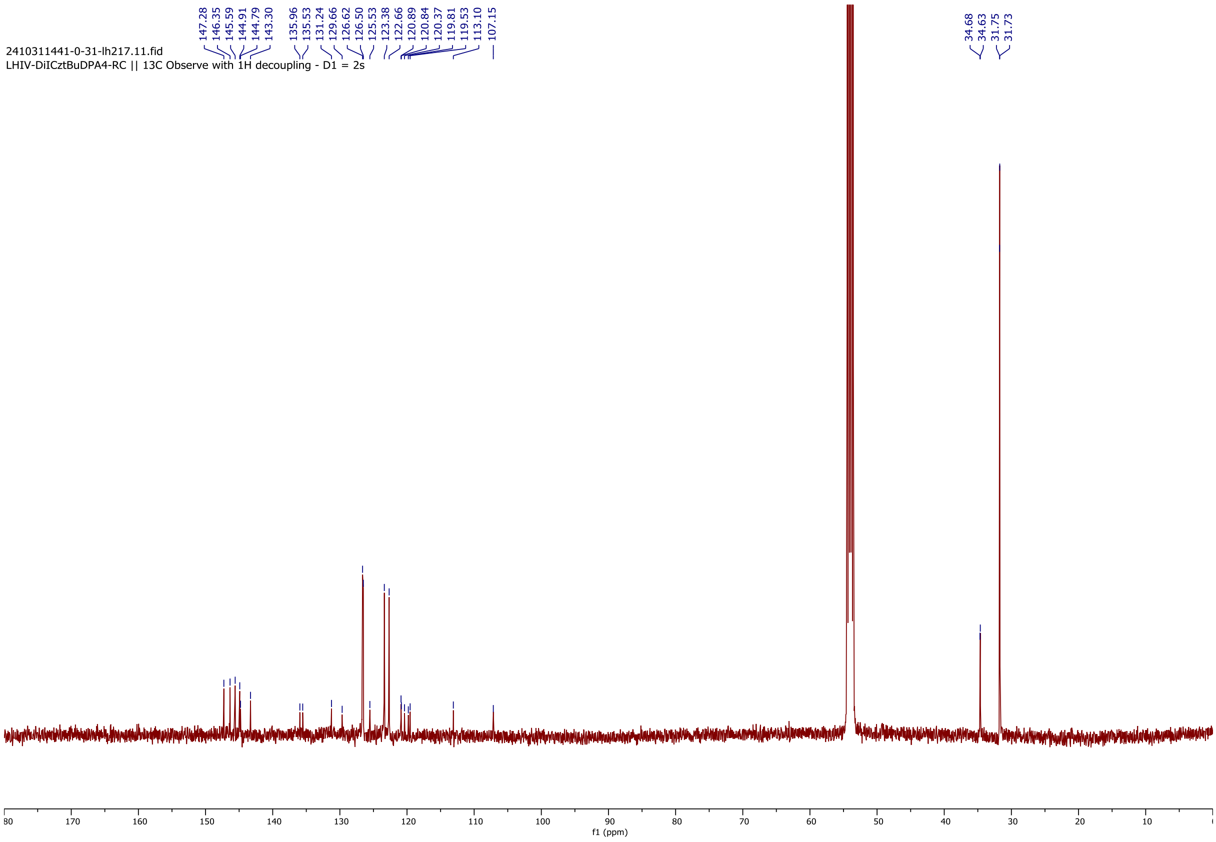

Figure **S34**. ^{13}C NMR spectrum of **DiICztBuDPA$_4$** in CD_2Cl_2.

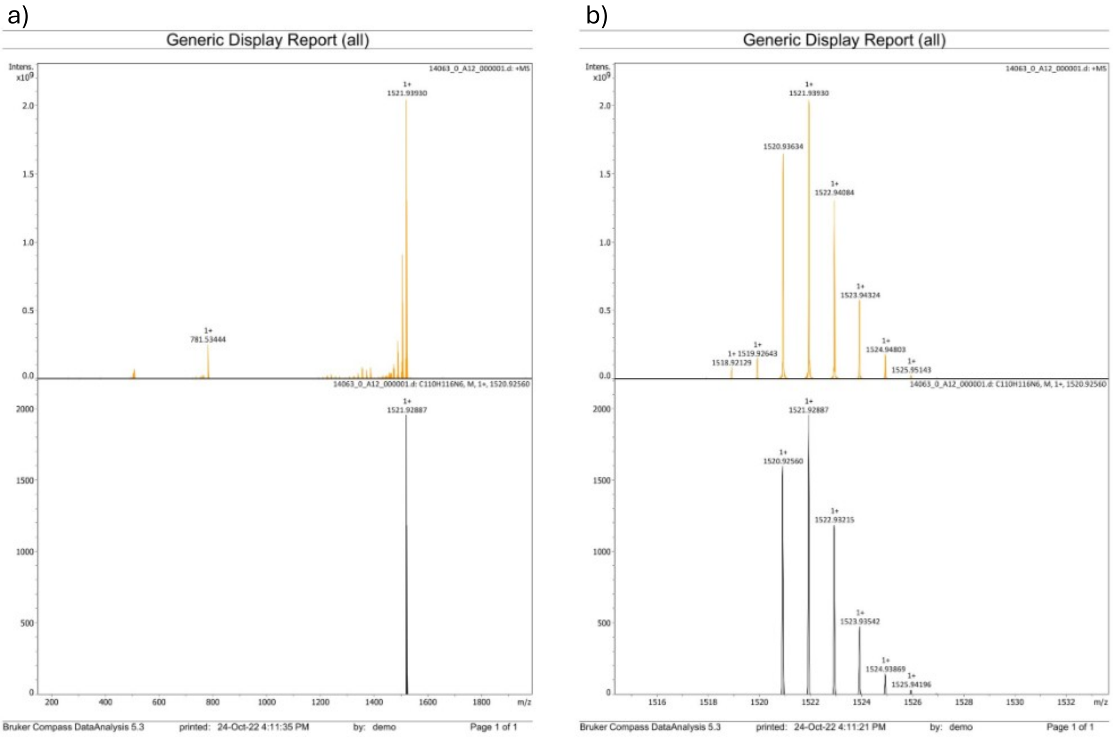

Figure **S35**. MALDI-TOF HRMS $[M]^+$ trace of **DiICztBuDPA$_4$.**

HPLC Trace Report06Oct2022

<Sample Information>

Sample Name	: diicz-dpa4tbu-ppt-meoh-tol try 2		
Sample ID	:		
Method Filename	: 100% THF 20 mins 280nm - DH.lcm		
Batch Filename	: diicz.lcb		
Vial #	: 1-13	Sample Type	: Unknown
Injection Volume	: 10 uL		
Date Acquired	: 05/10/2022 13:30:14	Acquired by	: System Administrator
Date Processed	: 05/10/2022 13:50:15	Processed by	: System Administrator

<Chromatogram>

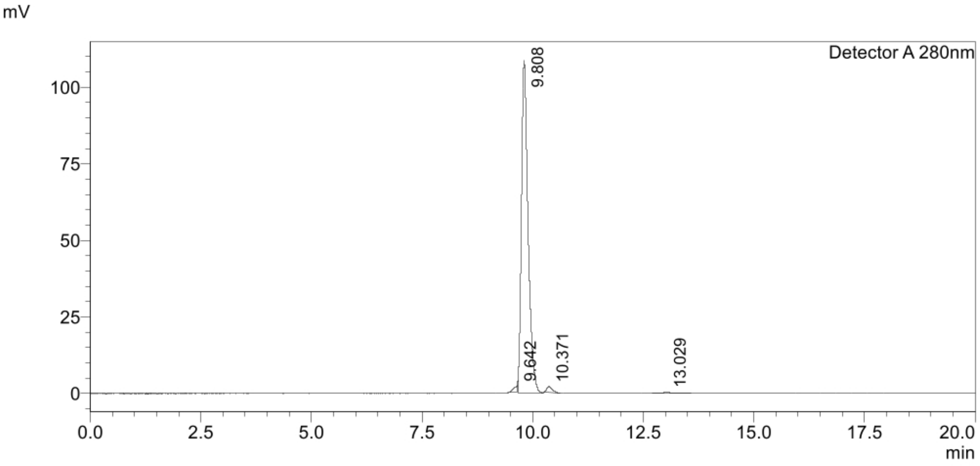

<Peak Table>

Detector A 280nm

Peak#	Ret. Time	Area	Height	Area%	Area/Height	Width at 5% Height
1	9.642	11937	1959	1.031	6.094	--
2	9.808	1122535	108120	96.986	10.382	0.375
3	10.371	18374	1962	1.587	9.365	0.306
4	13.029	4569	432	0.395	10.566	0.345
Total		1157416	112473	100.000		

Figure **S36**. GPC-HPLC trace **DiICztBuDPA$_4$**.

Photophysical measurements

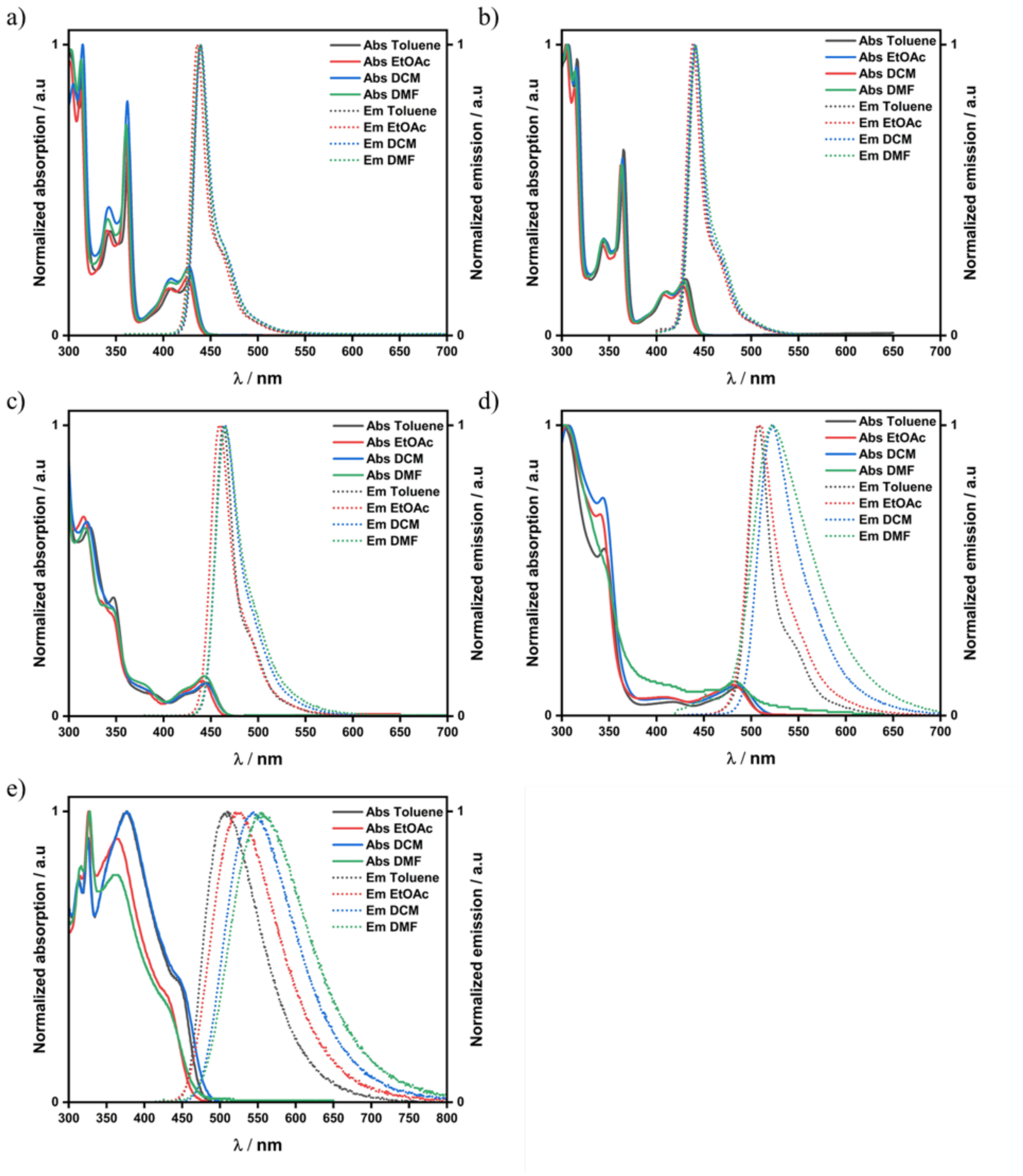

Figure **S37**. Absorption and steady-state emission spectra as a function of solvent for a) **DiICztBu$_4$**, (λ_{exc} = 340 nm) b) **DiICzMes$_4$** spectra taken from ref. [16] (λ_{exc} = 380 nm), c) **DiICztBuCz$_4$** (λ_{exc} = 340 nm), d) **DiICztBuDPA$_4$** (λ_{exc} = 400 nm), and e) **4CzIPN** (λ_{exc} = 378 nm). Measurements performed at room temperature under air.

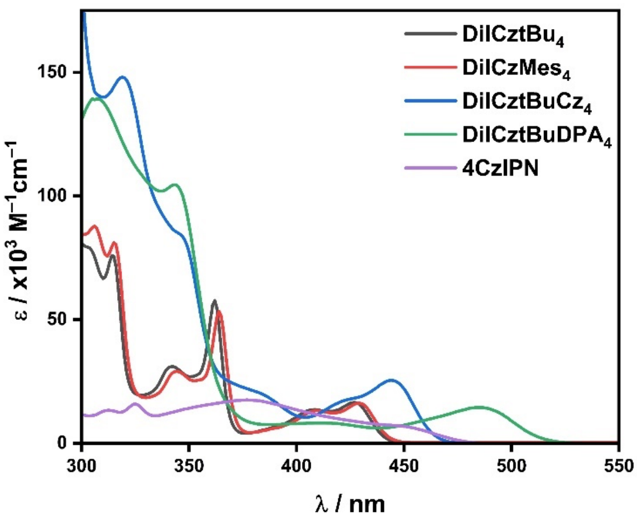

Figure **S38**. Absorption spectra of **DiICztBu$_4$**, **DiICzMes$_4$**, **DiICztBuCz$_4$**, **DiICztBuDPA$_4$**, and **4CzIPN** in DCM.

Table **S1**. Absorption data for **DiICztBu$_4$**, **DiICzMes$_4$**, **DiICztBuCz$_4$**, **DiICztBuDPA$_4$**, and **4CzIPN**.[a]

Solvent	λ_{abs} / nm (ε / 10^3 M^{-1} cm^{-1})				
	**DiICztBu$_4$**	**DiICzMes$_4$**[b]	**DiICztBuCz$_4$**	**DiICztBuDPA$_4$**	**4CzIPN**
Toluene	300, 315, 344, 363, 409, 429	302, 307, 316, 345, 365, 410, 431	322, 347, 390s, 422s, 446	302, 345, 418, 485	313, 325, 376, 449s
EtOAc	313, 341, 360, 406, 425	304, 313, 342, 362, 407, 428	316, 341s, 382s, 418s, 440	306, 340, 409, 480	315, 326, 366, 433s
DCM	305 (58), 315 (67), 343 (29), 362 (54), 408 (13), 427 (16)	300 (84), 306 (88), 315 (81), 344 (29), 364 (53), 409 (13), 430 (16)	319 (152), 347s (84), 385s (20), 420s (17), 444 (25)	305 (141), 343 (104), 411 (7), 485 (14)	313 (14), 325 (16), 377 (18), 450s (7)
DMF	303, 313, 341, 361, 408, 426	300, 305, 314, 344, 363, 409, 429	317, 346, 382s, 420s, 443	303, 413, 481	316, 328, 363, 434s

[a]Solution UV/Vis absorption measurements performed at room temperature. s = shoulder. [b]From ref. [16].

Table **S2**. Steady-state emission data for **DiICztBu$_4$**, **DiICzMes$_4$**, **DiICztBuCz$_4$**, **DiICztBuDPA$_4$**, and **4CzIPN**.[a]

Solvent	λ_{PL} / nm (FWHM)				
	**DiICztBu$_4$**	**DiICzMes$_4$**[b]	**DiICztBuCz$_4$**	**DiICztBuDPA$_4$**	**4CzIPN**
Toluene	439 (17)	441 (17)	463 (23)	507 (27)	509 (87)
EtOAc	436 (18)	439 (18)	459 (26)	508 (37)	525 (102)
DCM	440 (20)	441 (20)	465 (29)	522 (50)	545 (108)
DMF	439 (20)	442 (20)	467 (33)	523 (71)	556 (119)

[a]Solution measurements performed at room temperature at 10^{-5} M concentration. λ_{exc}(**DiICztBu$_4$**) = 340 nm, λ_{exc}(**DiICzMes$_4$**) = 380 nm, λ_{exc}(**DiICztBuCz$_4$**) = 340 nm, and **DiICztBuDPA$_4$** λ_{exc} = 400 nm. [b]From ref. [16]

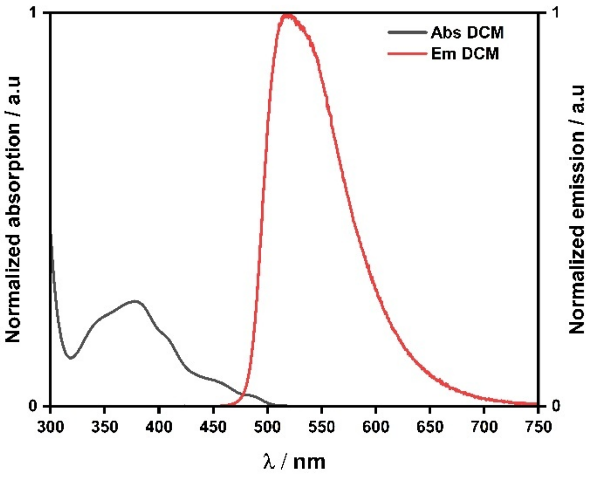

Figure **S39**. Absorption and steady-state emission spectra of ***fac*-Ir(ppy)$_3$** in DCM (λ_{exc} = 375 nm).

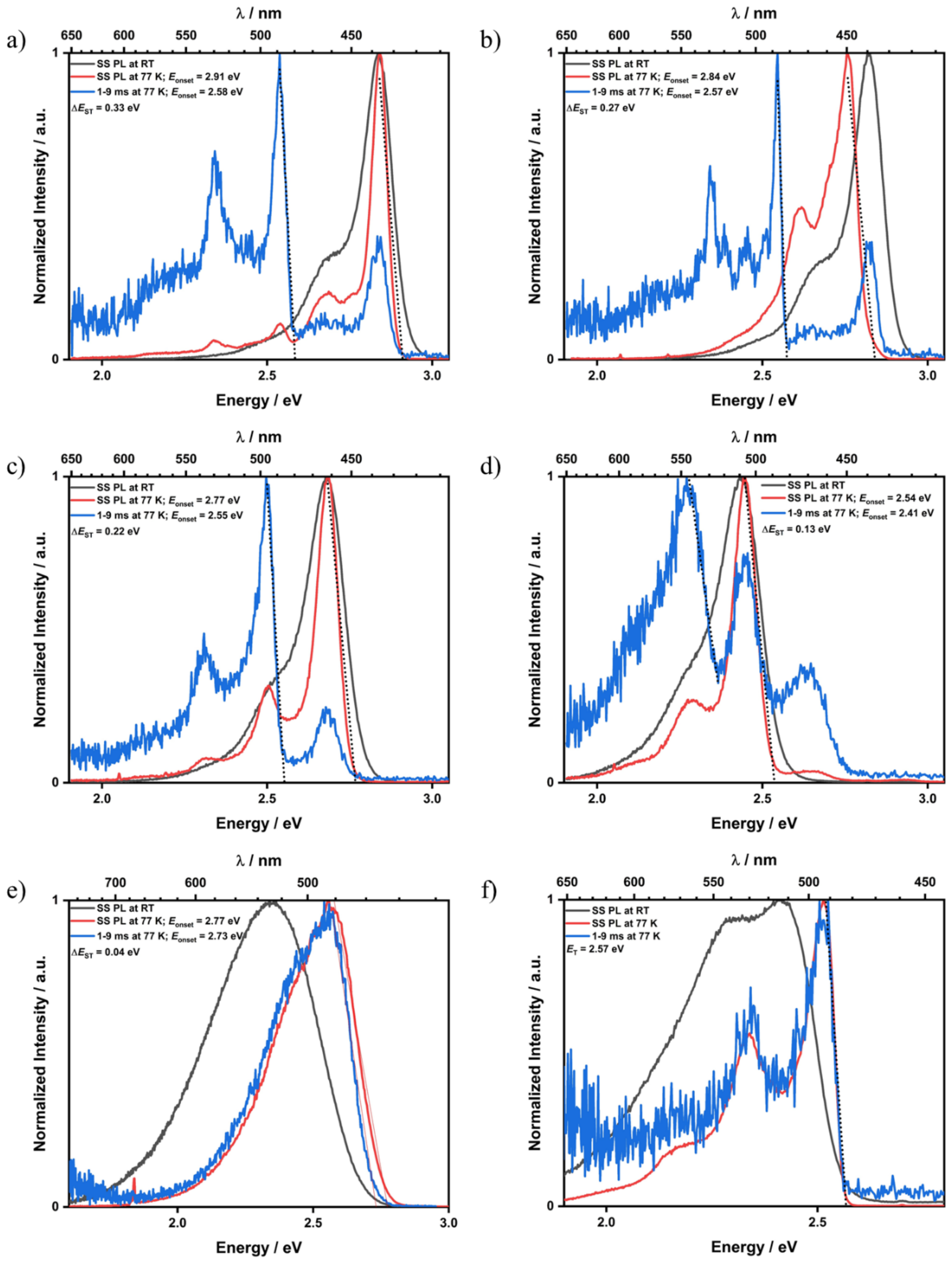

Figure **S40**. Steady-state photoluminescence spectra measured at room temperature and 77 K and phosphorescence spectra measured at 77 K in 2-MeTHF of a) **DiICztBu$_4$** (λ_{exc} = 390 nm), b) **DiICzMes$_4$** (λ_{exc} = 390 nm), c) **DiICztBuCz$_4$** (λ_{exc} = 390 nm), d) **DiICztBuDPA$_4$** (λ_{exc} = 400 nm), e) **4CzIPN** (λ_{exc} = 380 nm), and f) ***fac*-Ir(ppy)$_3$** (λ_{exc} = 375 nm) . The phosphorescence spectra were obtained over a 1-9 ms gated time range.

Table **S3**. Singlet and triplet energies of **DiICztBu$_4$**, **DiICzMes$_4$**, **DiICztBuCz$_4$**, **DiICztBuDPA$_4$**, and **4CzIPN** in 2-MeTHF.[a]

PC	E_{S1} / eV	E_{T1} / eV	ΔE_{ST} / eV
**DiICztBu$_4$**	2.91	2.58	0.33
**DiICzMes$_4$**	2.84	2.57	0.27
**DiICztBuCz$_4$**	2.77	2.55	0.22
**DiICztBuDPA$_4$**	2.54	2.41	0.13
**4CzIPN**	2.77	2.73	0.04
***fac*-Ir(ppy)$_3$**	–	2.57	–

[a]Values for E_{S1} taken from the onset of THE steady-state photoluminescence at 77 K. Values for E_{T1} from the onset of delayed emission at 77 K (gated 1-9 ms). λ_{exc}(**DiICztBu$_4$**) = 390 nm, λ_{exc}(**DiICzMes$_4$**) = 390 nm, λ_{exc}(**DiICztBuCz$_4$**) = 390 nm, λ_{exc}(**DiICztBuDPA$_4$**) = 400 nm, λ_{exc}(**4CzIPN**) = 380 nm, and λ_{exc}(***fac*-Ir(ppy)$_3$**) = 375 nm.

The PL spectra of **DiICztBuCz$_4$** and **DiICztBuDPA$_4$** reveal that there is a minimal positive solvatochromism across the range of solvents of differing polarity that is consistent with their excited states having SRCT character (Figure **S36**). The photoluminescence quantum yields (Φ_{PL}) in DCM are 21, 26, 39, and 36% for **DiICztBu$_4$**, **DiICzMes$_4$**, **DiICztBuCz$_4$** and **DiICztBuDPA$_4$**, respectively, which decrease to 18, 22, 34, and 26% under aerated conditions. There is no delayed emission and PL lifetimes range from 9-16 ns (Figure **S41** and Figure **S42**). Given the moderately large ΔE_{ST}, it is not uncommon for MR-TADF emitters to not show delayed emission in solution as non-radiative decay competes with RISC, while in the solid state this is largely suppressed and delayed emission becomes apparent. **DiICztBu$_4$** and **DiICzMes$_4$** have reported delayed fluorescence lifetimes, τ_{DF}, of 12.5 ms as 1 wt% doped films in mCP:TSPO 1:1 and τ_{DF} = 433 μs as 3 wt% doped films in mCP, respectively, while there was no observed delayed emission in solution.[14, 16]

Table **S4**. Photoluminescence quantum yields in DCM of **DiICztBu$_4$**, **DiICzMes$_4$**, **DiICztBuCz$_4$**, and **DiICztBuDPA$_4$**.[a]

PC	Φ_{PL} / % under N_2	Φ_{PL} / % under Air
**DiICztBu$_4$**	21	18
**DiICzMes$_4$**	26	22
**DiICztBuCz$_4$**	39	34
**DiICztBuDPA$_4$**	36	26

[a]The PLQY of the emitters were determined using the optically dilute method[3] using quinine sulfate as the external reference (Φ_{PL} = 54.6% in 0.5 M H_2SO_4).[4] N_2 measurements were performed after three freeze-pump-thaw cycles and the solutions were then opened to air for aerated samples. λ_{exc} = 360 nm.

Table **S5**. Photophysical data for **DiICztBu$_4$**, **DiICzMes$_4$**, **DiICztBuCz$_4$**, and **DiICztBuDPA$_4$** in DCM.[a]

Compound	λ_{PL}	τ_{PF} / ns (air)	τ_{DF} / μs (air)
**DiICztBu$_4$**	440	9.3 (7.8)	–
**DiICzMes$_4$**	442	9.5 (7.8)	–
**DiICztBuCz$_4$**	466	9.6 (8.3)	–
**DiICztBuDPA$_4$**	523	16.3 (12.8)	–
**4CzIPN**	545	25.6 (20)	3.03 (0.66)
***fac*-Ir(ppy)$_3$**	520	–	1.39 (0.05)

[a]Excitation wavelength for steady-state emission **DiICztBu$_4$** (λ_{exc} = 390 nm), **DiICzMes$_4$** (λ_{exc} = 390 nm), **DiICztBuCz$_4$** (λ_{exc} = 380 nm), **DiICztBuDPA$_4$** (λ_{exc} = 400 nm), **4CzIPN** (λ_{exc} = 375 nm), and ***fac*-Ir(ppy)$_3$** (λ_{exc} = 375 nm). Excitation wavelength for time-resolved measurement λ_{exc} = 374.9 nm for all compounds.

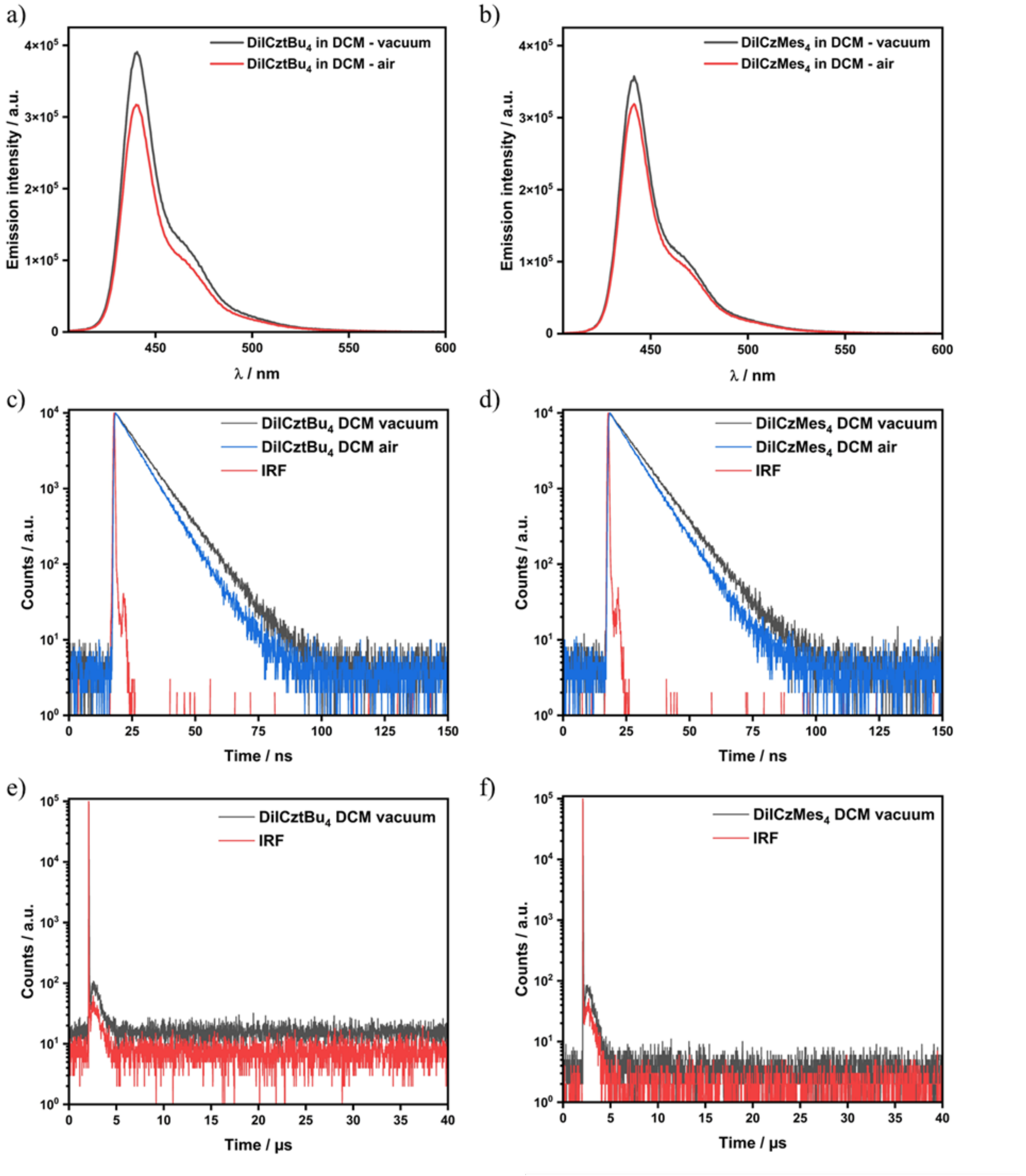

Figure **S41**. Photoluminescence measurements of **DiICzMes$_4$** and **DiCztBu$_4$**. a) Steady-state emission of **DiICztBu$_4$** deaerated and in air in DCM (λ_{exc} = 390 nm). b) Steady-state emission of **DiICzMes$_4$** deaerated and in air in DCM (λ_{exc} = 390 nm). Room temperature time-resolved photoluminescence decays for c) **DiICztBu$_4$** and d) **DiICzMes$_4$** in DCM deaerated and in air (λ_{exc} = 375 nm, 200 ns time range). Room temperature time-resolved photoluminescence decay for e) **DiICztBu$_4$** and f) **DiICzMes$_4$** in DCM deaerated and in air (λ_{exc} = 375 nm, 40 µs time range). IRF is the instrument response function.

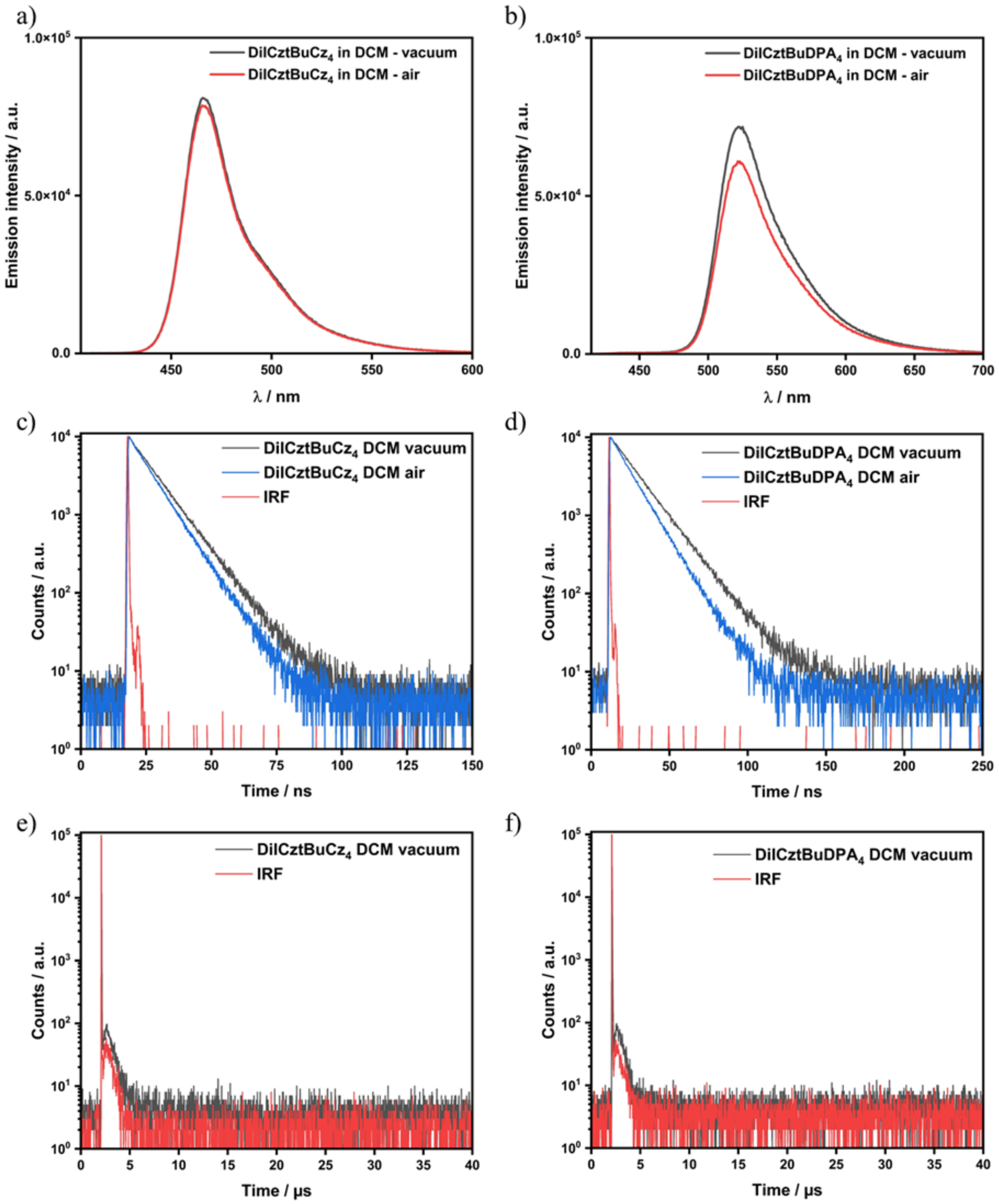

Figure **S42**. Photoluminescence measurements of **DiICztBuCz$_4$** and **DiCztBuDPA$_4$**. a) Steady-state emission spectra of **DiICztBu$_4$** deaerated and in air in DCM (λ_{exc} = 380 nm). b) Steady-state emission spectra of **DiCztBuDPA$_4$** deaerated and air in DCM (λ_{exc} = 400 nm). Room temperature time-resolved photoluminescence decays for c) **DiICztBuCz$_4$** and d) **DiCztBuDPA$_4$** in DCM deaerated and in air (λ_{exc} = 375 nm, 200 ns time range). Room temperature time-resolved photoluminescence decays for e) **DiICztBuCz$_4$** and f) **DiCztBuDPA$_4$** both in DCM deaerated and in air (λ_{exc} = 375 nm, 40 μs time range). IRF is the instrument response function.

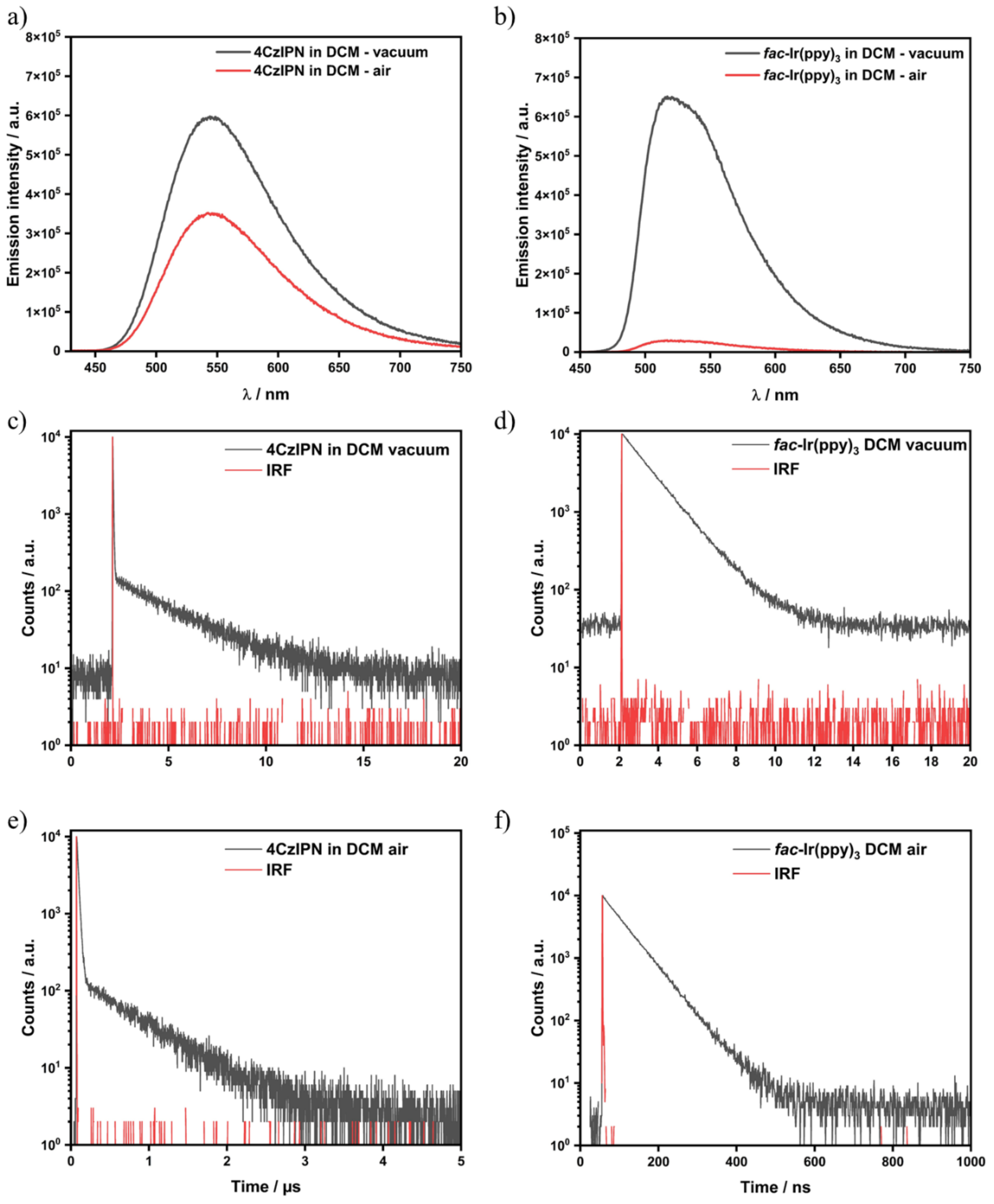

Figure **S43**. Photoluminescence measurements of **4CzIPN** and ***fac*-Ir(ppy)$_3$**. a) Steady-state emission spectra of **4CzIPN** deaerated and in air in DCM (λ_{exc} = 375 nm). b) Steady-state emission spectra of ***fac*-Ir(ppy)$_3$** deaerated and air in DCM (λ_{exc} = 375 nm). Room temperature time-resolved photoluminescence decays for c) **4CzIPN** and d) ***fac*-Ir(ppy)$_3$** in DCM under deaerated (λ_{exc} = 375 nm, 20 μs time range). Room temperature time-resolved photoluminescence decays for e) **4CzIPN** and f) ***fac*-Ir(ppy)$_3$** both in DCM under air (λ_{exc} = 375 nm, 5 μs & 1 μs time range for **4CzIPN** and ***fac*-Ir(ppy)$_3$** respectively). IRF is the instrument response function.

Table **S6**. Time-resolved PL measurements of **4CzIPN** in DCM.[a]

	τ_{PF} / ns	ω_1 / %	τ_{DF} / ns	ω_2 / %	τ_{AVG} / ns
**Deaerated**	25.6	37.8	3033.8	62.2	1895.5
**Air**	20.0	69.1	660.2	30.9	217.8

[a]An EPL-UV picosecond pulsed diode laser (Edinburgh Instruments, EPL-375, λ_{exc} = 375 nm) was used to collect time-resolved PL decays. Time-resolved PL measurements were fitted to a sum of exponentials decay model.

Photocatalysis

Photocatalysis experiments were carried out using a photoreactor containing a 3D printed custom-built vessel holder, itself after a design from ref [17], as shown in Figure **S44**. The vessel holder is placed in a mirrored box with fans on two sides to allow for cooling. This box is placed on a stirring plate. The reactions were carried out in 10 mL pressure vessels (CEM) in which eight reactions can be run in parallel. The reactions were irradiated using a Kessil PR160L LED source (λ_{exc} = 440 nm). The maximum intensity was chosen for all reactions. With a distance of ~ 8 cm between light source and vial this translates to photon flux of between 80 000 and 100 000 mW/cm^2 according to the Kessil Lamp specifications.

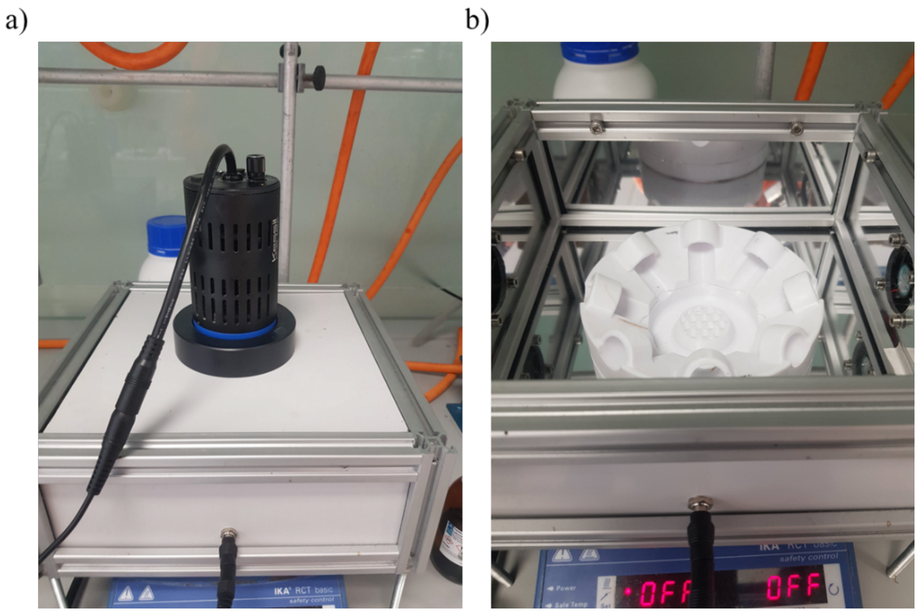

Figure **S44**. Experimental setup for photocatalysis reactions.

After completion of the reaction, the products were analysed by ^{1}H NMR spectroscopy with 1,3,5-trimethoxybenzene as the internal standard. All yields reported are the mean from at least two reactions with the associated standard deviation.

***E/Z* isomerisation**

General procedure 4

To an oven-dried vial was added the PC (1 mol%, 2.00 μmol) . In the case of reactions under a nitrogen atmosphere, three vacuum/nitrogen cycles were carried out before dry DCM (1 mL, 0.2 M) followed by the substrate (0.20 mmol, 1.00 equiv.) were added. The solution was bubbled with N_2 for 5 mins. The solution was then stirred and irradiated (λ_{exc} = 440 nm). The *E*/*Z* ratios were determined by ^{1}H NMR spectroscopy.

General procedure 5

For reactions under air, the PC (1 mol%, 2.00 μmol) was dissolved in DCM (1 mL, 0.2 M) in an oven-dried vial followed by the addition of the substrate (0.20 mmol, 1.00 equiv.). The solution was stirred and irradiated (λ_{exc} = 440 nm). The *E*/*Z* ratios were determined by ^{1}H NMR spectroscopy.

***E/Z* of cinnamyl acetate**

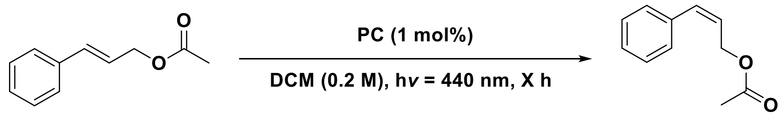

The reactions were carried out following General Procedures 4 and 5 for reactions under N_2 and air, respectively using cinnamyl acetate (35.2 mg, 0.20 mmol, 1.00 equiv.) (Table **S5)**. The *E*/*Z* ratios were determined by ^{1}H NMR spectroscopy.

The reaction progression with **DiICztBuDPA$_4$** was assessed only under N_2 as this reaction is completely quenched when oxygen is present. As a result of the ostensibly poorer spectral overlap due to its lower E_T **DiICztBuDPA$_4$** photocatalyzes the reaction significantly slower than the other three PCs of this family, where after 60 min the *E*/*Z* ratio is 60/40. The slower rate of isomerization may explain why, for this PC, oxygen acts as a more effective competitive quencher. Given that there is no significant difference in the τ_{PL} under N_2 or in air, we can rule out this parameter as governing the reaction rates (Figure **S42**). Photostability studies of **DiICztBuDPA$_4$** reveal a more significant change after the reaction both in air and under N_2 than using the other PCs (Figure **S46d**). This implies that there may be an O_2-induced photochemical degradation of **DiICztBuDPA$_4$**, which would explain why the *E/Z* isomerization reaction does not proceed in air with this PC. Under N_2, there is an observed change in the absorption spectrum of **DiICztBuDPA$_4$** after 24 h of reaction; however, this spectral change is different to the one observed in air. Under N_2, the SRCT band at λ_{abs} = 485 nm remains, yet there is a slight red-shift in its onset compared to before irradiation (new λ_{abs} = 491 nm). It is not clear what effect, if any, this spectral change has on the performance of the PC in the *E*/*Z* isomerization of cinnamyl acetate (Figure **S46d)** nor were we able to detect or isolate any photodegradation products.

Table **S7**. *E*/*Z* isomerisation yields for the isomerisation of cinnamyl acetate.[a]

PC	Conditions	Time	*E*/*Z* ratio
**No PC**	N_2	24 h	99/1 ± 1
**No PC**	Air	24 h	99/1 ± 1
**DiICztBu$_4$**	N_2	24 h	18/82 ± 0
**DiICztBu$_4$**	Air	24 h	19/81 ± 0
**DiICztBu$_4$**	Air	1 h	18/82 ± 0
**DiICztBu$_4$**	Air	0.5 h	19/82 ± 1
**DiICztBu$_4$**	Air	15 min	23/77 ± 2
**DiICztBu$_4$**	Air	10 min	36/64 ± 3
**DiICztBu$_4$**	Air	5 min	61/39 ± 0
**DiICztBu$_4$**	Air	2 min	81/19 ± 2
**DiICzMes$_4$**	N_2	24 h	19/81 ± 0
**DiICzMes$_4$**	Air	24 h	16/84 ± 1
**DiICzMes$_4$**	Air	1 h	19/81 ± 1
**DiICzMes$_4$**	Air	0.5 h	20/80 ± 0
**DiICzMes$_4$**	Air, **Dark**	0.5 h	100/0 ± 0
**DiICzMes$_4$**	Air	15 min	22/78 ± 1
**DiICzMes$_4$**	Air	10 min	31/69 ± 1
**DiICzMes$_4$**	Air	5 min	53/47 ± 2
**DiICzMes$_4$**	Air	2 min	78/22 ± 2
**DiICztBuCz$_4$**	N_2	24 h	19/81 ± 1
**DiICztBuCz$_4$**	Air	24 h	20/80 ± 0
**DiICztBuCz$_4$**	Air	1 h	19/81 ± 0
**DiICztBuCz$_4$**	Air	0.5 h	19/81 ± 1
**DiICztBuCz$_4$**	Air	15 min	27/73 ± 2
**DiICztBuCz$_4$**	Air	10 min	37/63 ± 4
**DiICztBuCz$_4$**	Air	5 min	56/44 ± 2
**DiICztBuCz$_4$**	Air	2 min	85/15 ± 1
**DiICztBuDPA$_4$**	N_2	24 h	28/72 ± 0
**DiICztBuDPA$_4$**	N_2	1 h	60/40 ± 1
**DiICztBuDPA$_4$**	N_2	0.5 h	75/25 ± 2
**DiICztBuDPA$_4$**	N_2	15 min	88/12 ± 1
**DiICztBuDPA$_4$**	N_2	10 min	92/8 ± 1
**DiICztBuDPA$_4$**	N_2	5 min	97/3 ± 1
**DiICztBuDPA$_4$**	N_2	2 min	99/1 ± 0
**DiICztBuDPA$_4$**	Air	24 h	98/2 ± 0

**4CzIPN**	N_2	24 h	14/86 ± 0
**4CzIPN**	Air	24 h	15/85 ± 0
**4CzIPN**	Air	1 h	39/61 ± 2
**4CzIPN**	Air	0.5 h	57/43 ± 2
**4CzIPN**	Air	15 min	78/22 ± 3
**4CzIPN**	Air	10 min	86/14 ± 2
**4CzIPN**	Air	5 min	94/6 ± 1
**4CzIPN**	Air	2 min	97/3 ± 1

[a]*E*/*Z* ratios were determined by ^1H NMR spectroscopy.

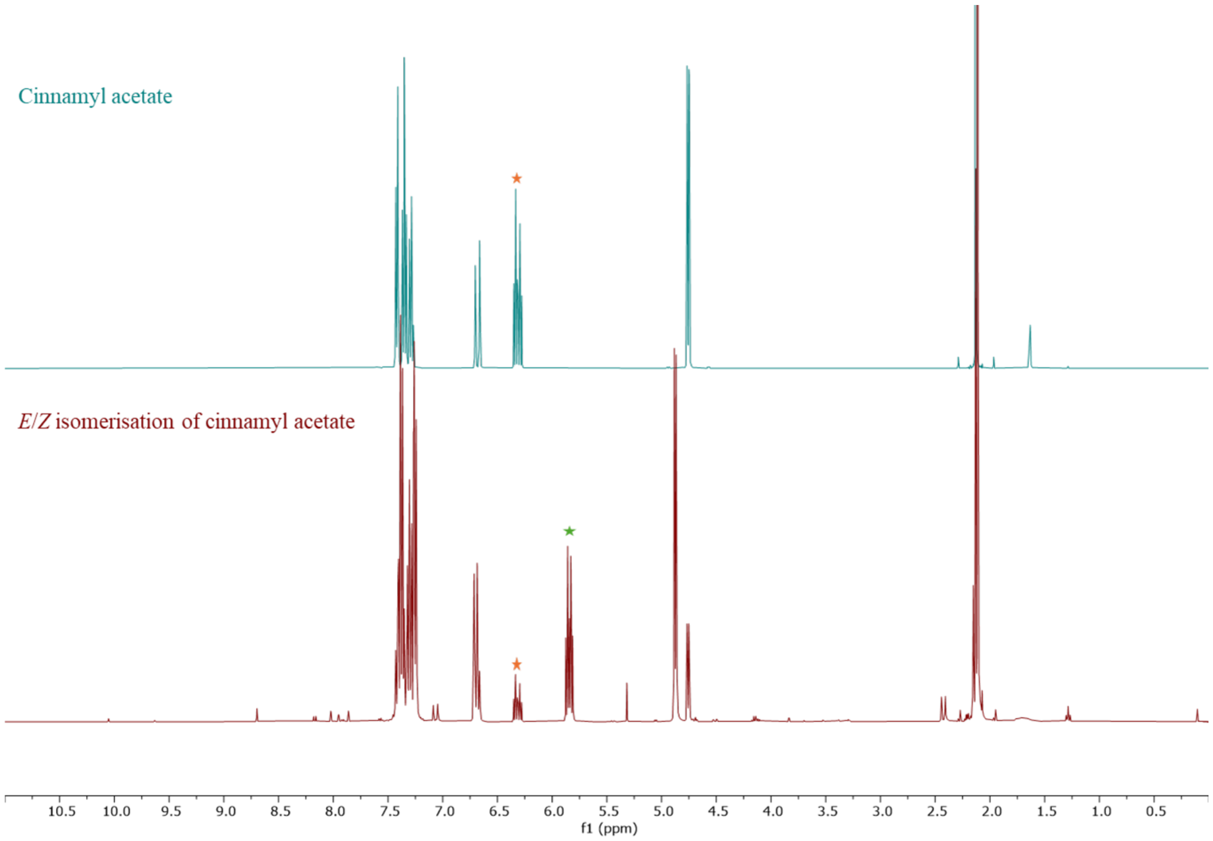

Figure **S45**. Example of the crude NMR spectral analysis for the *E*/*Z* isomerisation of cinnamyl acetate. Cinnamyl acetate (0.20 mmol) and PC (1 mol%) in DCM (1.0 mL). The reaction was irradiated (λ_{exc} = 440 nm) for 2 h. The green star marks the product resonances and the orange star marks the remaining starting material resonances.

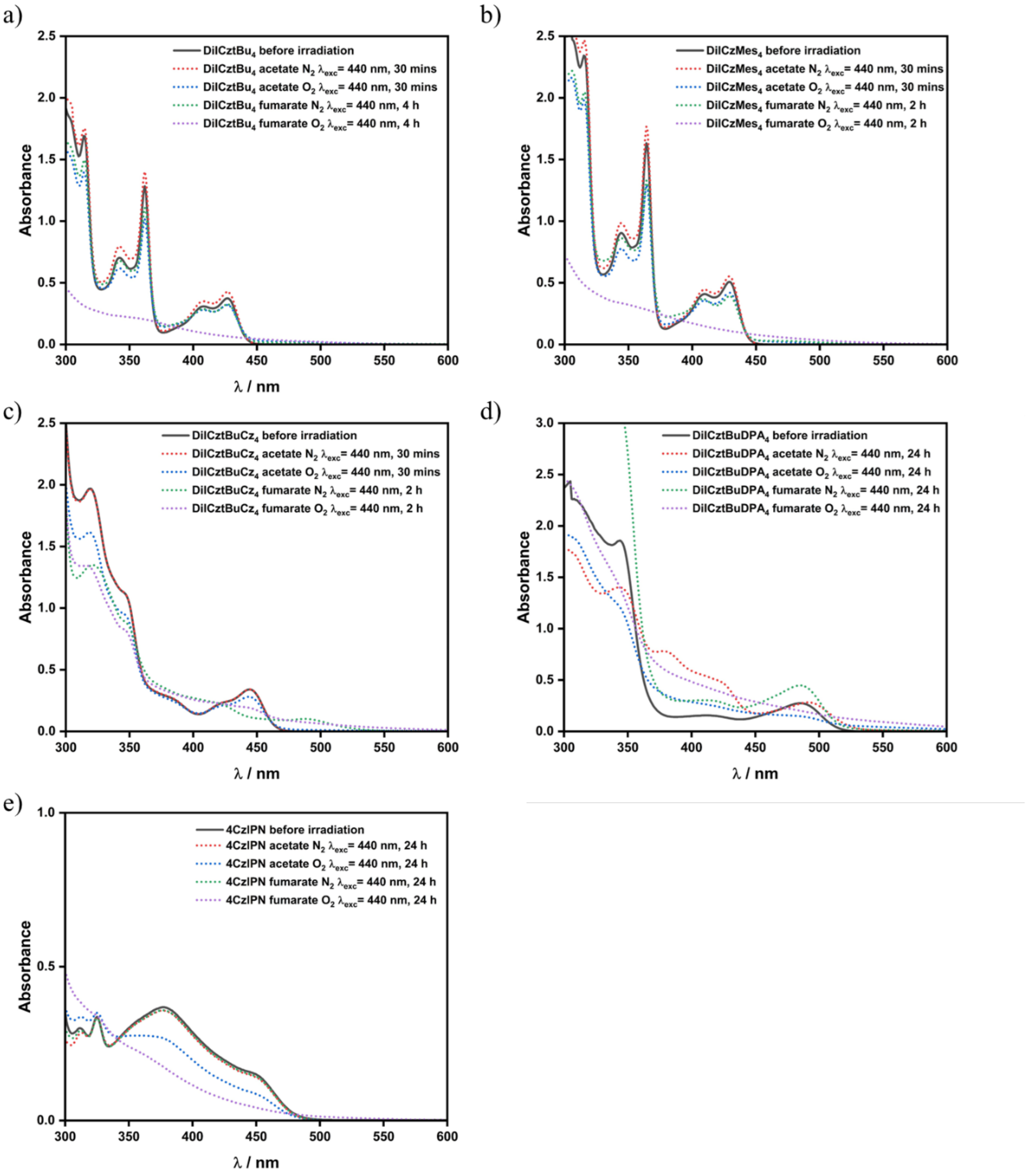

Figure **S46**. Absorption photostability study of the reaction mixture of the *E*/*Z* isomerisation of cinnamyl acetate and di*iso*propylfumarate photocatalysed by a) **DiICztBu$_4$**, b) **DiICzMes$_4$**, c) **DiICztBuCz$_4$**, d) **DiICztBuDPA$_4$**, and e) **4CzIPN** under a N_2 atmosphere. Absorption compared before and after 30 min irradiation at λ_{exc} = 440 nm for **DiICztBu$_4$**, **DiICzMes$_4$**, **DiICztBuCz$_4$**, and after 24 h for **DiICztBuDPA$_4$** and **4CzIPN**.

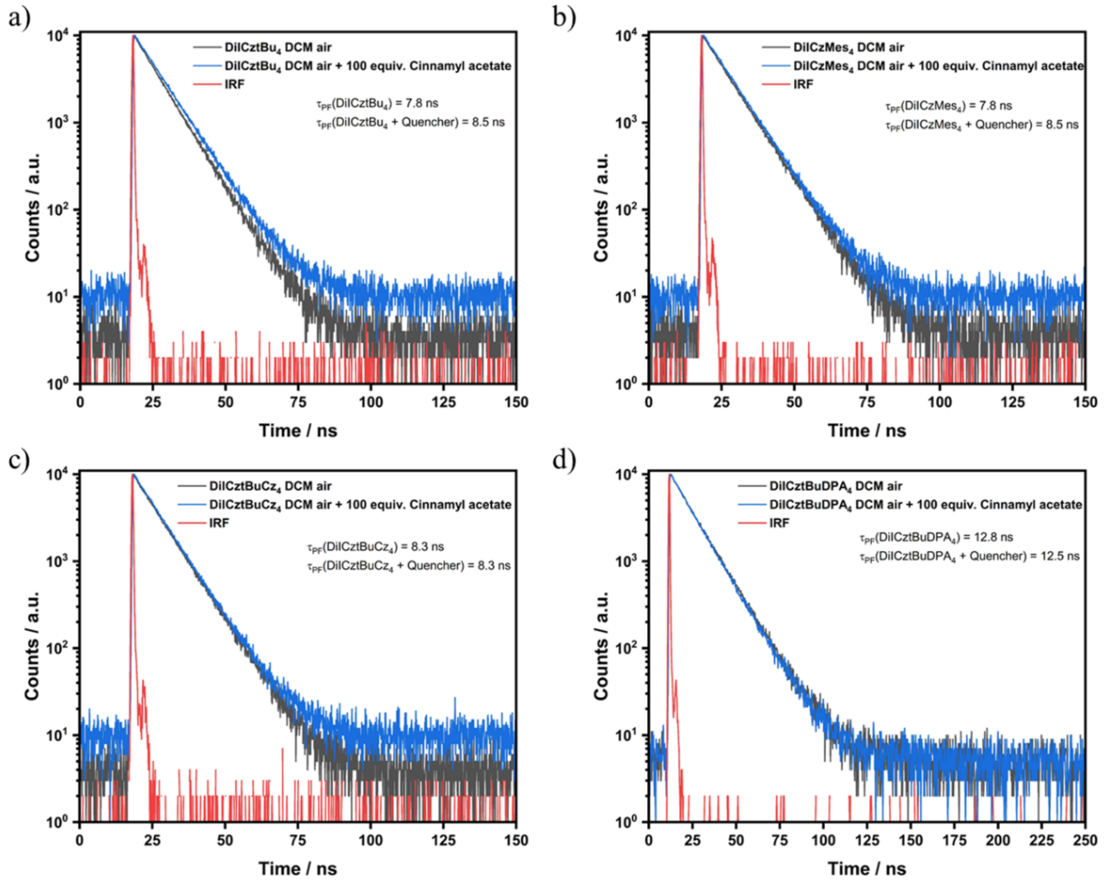

Figure **S47**. Room temperature time-resolved photoluminescence decays for a) **DiICztBu4**, b) **DiCztBuMes4**, c) **DiICztBuCz4**, and d) **DiICztBuDPA4** in DCM in aerated conditions and in aerated conditions in the presence of 100 equiv. *E*-cinnamyl acetate (λ_{exc} = 375 nm, 200 and 500 ns time range). IRF is the instrument response function.

***E/Z* isomerization of di*iso*propyl fumarate**

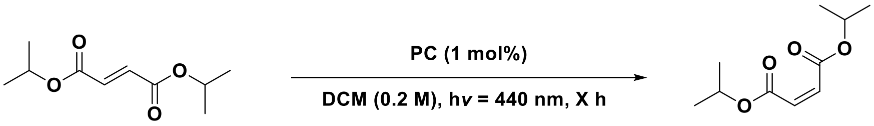

The reactions were carried out following General Procedures 4 and 5 for reactions under N_2 and air, respectively, using cinnamyl acetate (40.1 mg, 0.20 mmol, 1.00 equiv.) (Table **S5)**. The *E*/*Z* ratios were determined by ^{1}H NMR spectroscopy.

Table **S8**. *E*/*Z* isomerisation yields for the isomerisation of di*iso*propyl fumarate.[a]

PC	Conditions	Time	*E*/*Z* ratio
**No PC**	N_2	24 h	100/0 ± 0
**No PC**	Air	24 h	100/0 ± 0
**DiICztBu$_4$**	N_2	24 h	13/87 ± 0
**DiICztBu$_4$**	Air	24 h	95/5 ± 1
**DiICztBu$_4$**	N_2	4 h	13/87 ± 1
**DiICztBu$_4$**	N_2	2 h	26/74 ± 8
**DiICztBu$_4$**	N_2	1 h	59/41 ± 1
**DiICztBu$_4$**	N_2	0.5 h	78/22 ± 6
**DiICztBu$_4$**	N_2	15 min	89/11 ± 0
**DiICzMes$_4$**	N_2	24 h	13/87 ± 0
**DiICzMes$_4$**	Air	24 h	92/8 ± 2
**DiICzMes$_4$**	N_2	4 h	12/88 ± 0
**DiICzMes$_4$**	N_2	2 h	12/88 ± 0
**DiICzMes$_4$**	N_2	2 h, **Dark**	100/0 ± 0
**DiICzMes$_4$**	N_2	1 h	21/79 ± 3
**DiICzMes$_4$**	N_2	0.5 h	58/42 ± 3
**DiICzMes$_4$**	N_2	15 min	80/20 ± 3
**DiICztBuCz$_4$**	N_2	24 h	66/34 ± 4
**DiICztBuCz$_4$**	Air	24 h	88/12 ± 0
**DiICztBuCz$_4$**	N_2	4 h	75/25 ± 0
**DiICztBuCz$_4$**	N_2	2 h	75/25 ± 1
**DiICztBuCz$_4$**	N_2	1 h	75/25 ± 1
**DiICztBuCz$_4$**	N_2	0.5 h	80/20 ± 2
**DiICztBuCz$_4$**	N_2	15 min	87/13 ± 0
**DiICztBuDPA$_4$**	N_2	24 h	100/0 ± 0
**DiICztBuDPA$_4$**	Air	24 h	100/0 ± 0
**4CzIPN**	N_2	24 h	69/31 ± 4

**4CzIPN**	Air	24 h	91/9 ± 4
**4CzIPN**	N_2	4 h	95/5 ± 1
**4CzIPN**	N_2	2 h	97/3 ± 0
**4CzIPN**	N_2	1 h	99/1 ± 0
**4CzIPN**	N_2	0.5 h	99/1 ± 0
**4CzIPN**	N_2	15 min	100/0 ± 0

[a] *E*/*Z* ratios were determined by ^{1}H NMR spectroscopy.

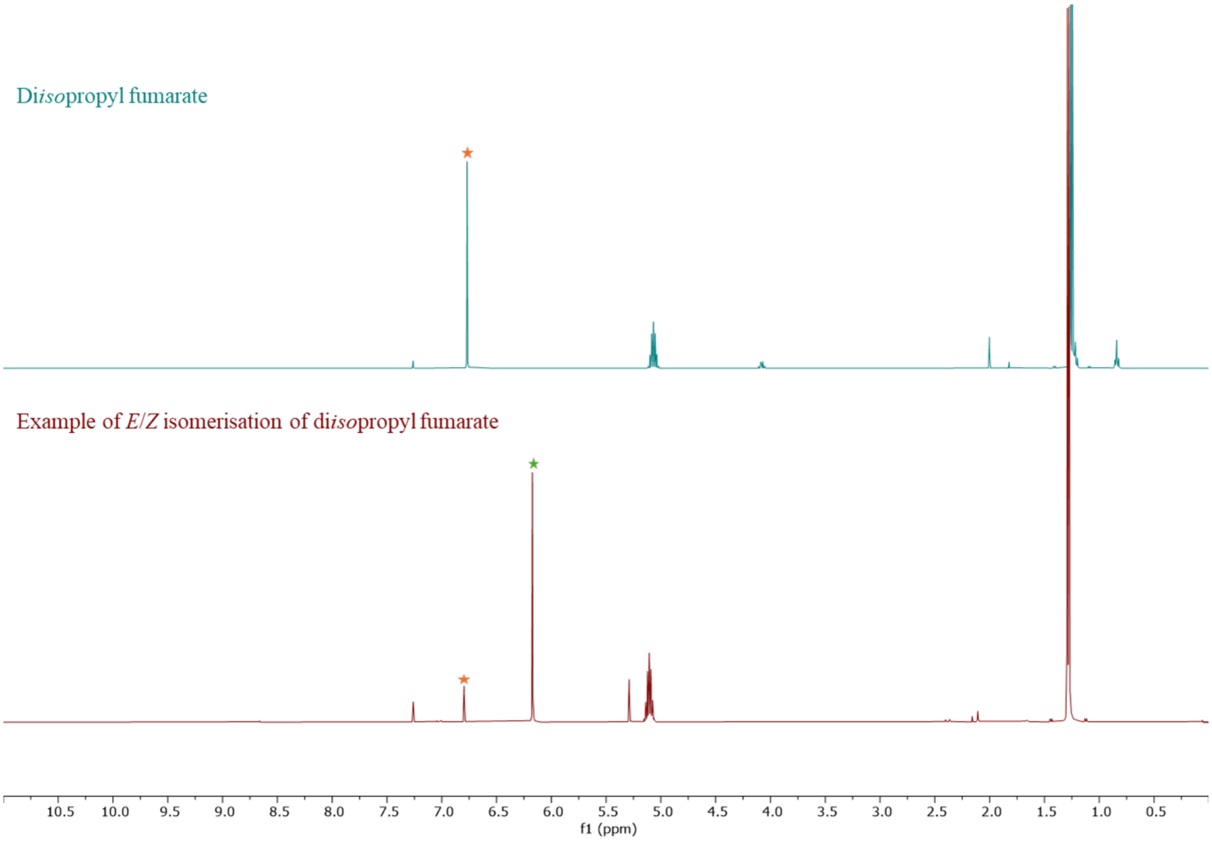

Figure **S48**. Example of the crude NMR spectral analysis of the *E*/*Z* isomerisation of di*iso*proyl fumarate. Di*iso*propyl fumarate (0.20 mmol) and PC (1 mol%) in DCM (1.0 mL). The reaction was irradiated (λ_{exc} = 440 nm) for 2 h. The green star marks the product resonances and the orange star marks the remaining starting material resonances.

[2+2] Cycloaddition of Norbornadiene

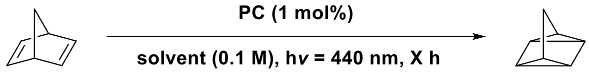

To an oven-dried vial was added the PC (1 mol%, 2.00 µmol) . In the case of reactions under N_2, three vacuum/nitrogen cycles were carried out before dry DCM (2 mL, 0.1 M) and norbornadiene (18.4 mg, 0.20 mmol, 1.00 equiv.) were added. The solution was bubbled with N_2 for 5 mins. The solution was then stirred and irradiated (λ_{exc} = 440 nm).

For reactions under air, the PC (1 mol%, 2.00 µmol) was dissolved in DCM (2 mL, 0.1 M) in an oven-dried vial and then norbornadiene (18.4 mg, 0.20 mmol, 1.00 equiv.) was added. The solution was stirred and irradiated (λ_{exc} = 440 nm). The yields were determined by ^{1}H NMR spectroscopy using 1,3,5-trimethoxybenzene as the internal standard. For **DiICztBuDPA$_4$** no photodegradation is observed, and this is because no reaction takes place either with norbornadiene or O_2 (Figure **S51d**). Instead, the intensity of the absorption spectrum after the reaction is slightly higher than before irradiation, which can be attributed to the poor solubility of this PC in the solution and where the concentration of dissolved PC slightly increases after stirring for 30 min.

Table **S9**. Yields of the [2+2] cycloaddition of norbornadiene.[a]

PC	Conditions	Time / h	Yield / %
**No PC**	N_2	0.5	0 ± 0
**No PC**	Air	0.5	0 ± 0
**DiICztBu$_4$**	N_2	2	87 ± 1
**DiICztBu$_4$**	N_2	0.5	89 ± 2
**DiICztBu$_4$**	Air	0.5	64 ± 4
**DiICzMes$_4$**	N_2	2	87 ± 1
**DiICzMes$_4$**	N_2	0.5	85 ± 5
**DiICzMes$_4$**	Air	0.5	71 ± 6
**DiICztBuCz$_4$**	N_2	2	84 ± 1
**DiICztBuCz$_4$**	N_2	0.5	88 ± 0
**DiICztBuCz$_4$**	Air	0.5	75 ± 2
**DiICztBuDPA$_4$**	N_2	2	4 ± 1
**DiICztBuDPA$_4$**	N_2	0.5	0 ± 0
**DiICztBuDPA$_4$**	Air	0.5	0 ± 0
**4CzIPN**	N_2	0.5	0 ± 0
**4CzIPN**	Air	0.5	0 ± 0

[a]Yields were determined by ^{1}H NMR spectroscopy using 1,3,5-trimethoxybenzene as the internal standard.

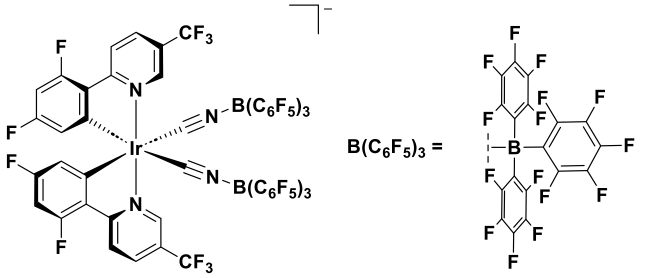

Figure **S49**. Structure of the Ir photocatalyst reported by Wenger and co-workers (E_T = 2.99 eV).[18]

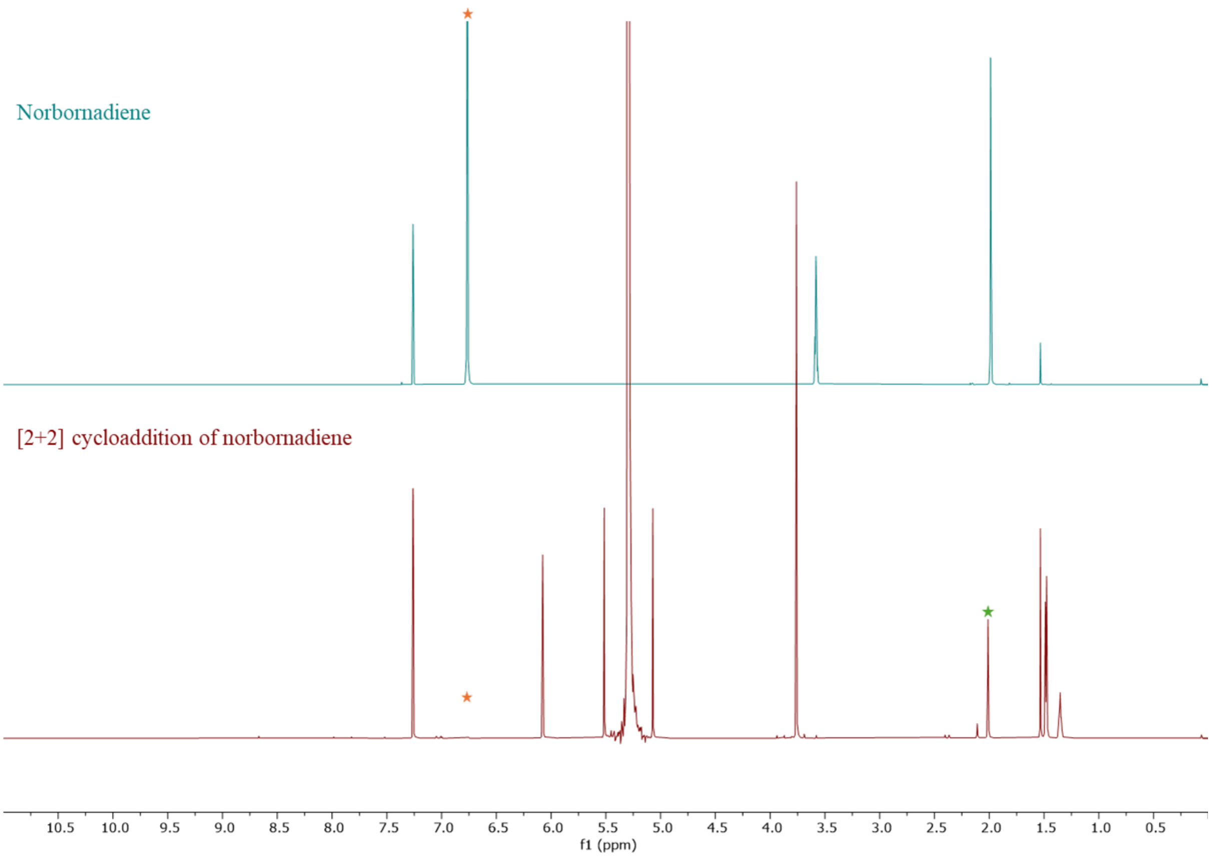

Figure **S50**. Example of the crude NMR spectral analysis of the [2+2] cycloaddition of norbornadiene. Norbornadiene (0.20 mmol) and PC (1 mol%) in DCM (2.0 mL). The reaction was irradiated (λ_{exc} = 440 nm) for 30 mins. The green star marks the product resonances and the orange star marks the remaining starting material resonances.

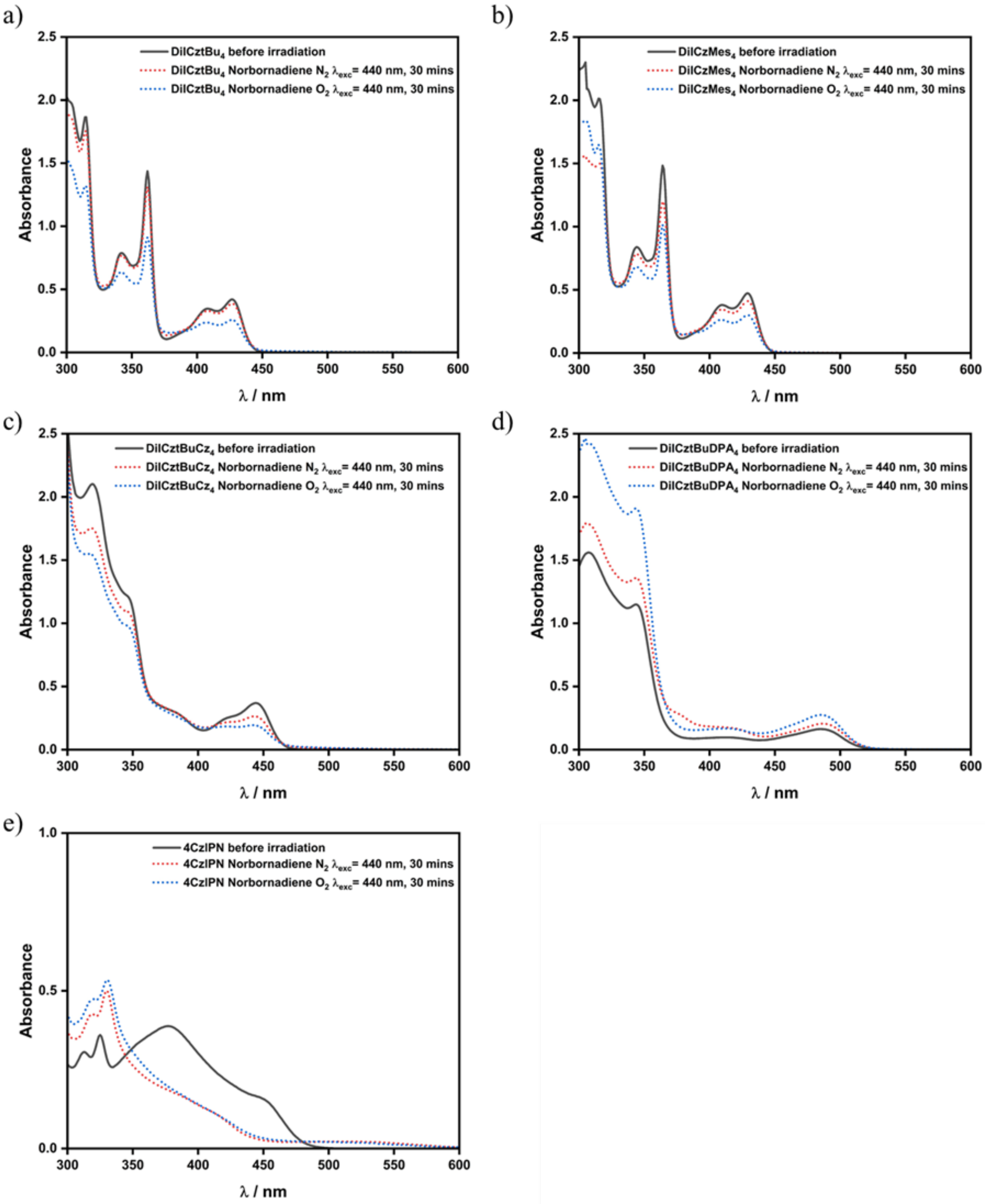

Figure **S51**. Absorbance spectra of the reaction mixture of the [2+2] cycloaddition of norbornadiene photocatalyzed by a) **DiICztBu$_4$**, b) **DiICzMes$_4$**, c) **DiICztBuCz$_4$**, d) **DiICztBuDPA$_4$**, and e) **4CzIPN**. Absorption spectra compared before and after 30 min irradiation at λ_{exc} = 440 nm.

Sigmatropic shift of (*S*)-Verbenone

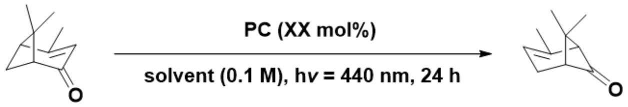

The PC (1 or 5 mol%, 2.00 or 10 µmol) was added to an oven-dried vial. Three vacuum/nitrogen cycles were carried out before dry DCM (2 mL, 0.1 M) and then *S*-verbenone (30.0 mg, 0.20 mmol, 1.00 equiv.) were added. The solution was bubbled with N_2 for 5 mins. The solution was then stirred and irradiated (λ_{exc} = 440 nm). The yields were determined by ^{1}H NMR spectroscopy using 1,3,5-trimethoxybenzene as the internal standard.

Table **S10**. Yields of the sigmatropic shift of (*S*)-verbenone.[a]

PC	PC loading / mol%	Yield / %
**No PC**	–	0 ± 0
**DiICztBu$_4$**	1	29 ± 3
**DiICztBu$_4$**	5	37 ± 1
**DiICzMes$_4$**	1	22 ± 2
**DiICzMes$_4$**	5	38 ± 6
**DiICztBuCz$_4$**	1	18 ± 1
**DiICztBuCz$_4$**	5	21 ± 2
**DiICztBuDPA$_4$**	1	0 ± 0
**4CzIPN**	5	5 ± 1

[a]Yields were determined by ^{1}H NMR spectroscopy using 1,3,5-trimethoxybenzene as the internal standard

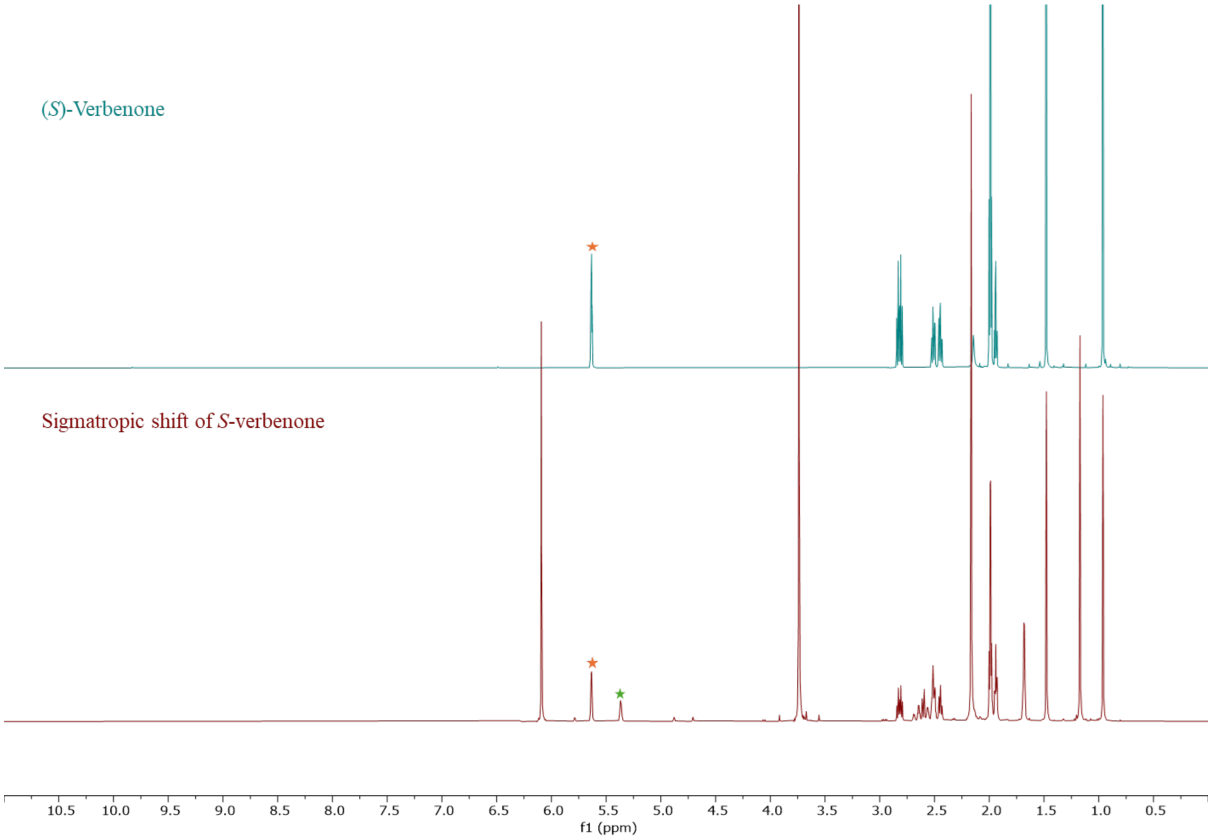

Figure **S52**. Example of the crude NMR spectral analysis of the sigmatropic shift of (*S*)-verbenone. (*S*)-verbenone (0.20 mmol) and PC (1 mol%) in DCM (2.0 mL). The reaction was irradiated (λ_{exc} = 440 nm) for 24 h. The green star marks the product resonances and the orange star marks the remaining starting material resonances.

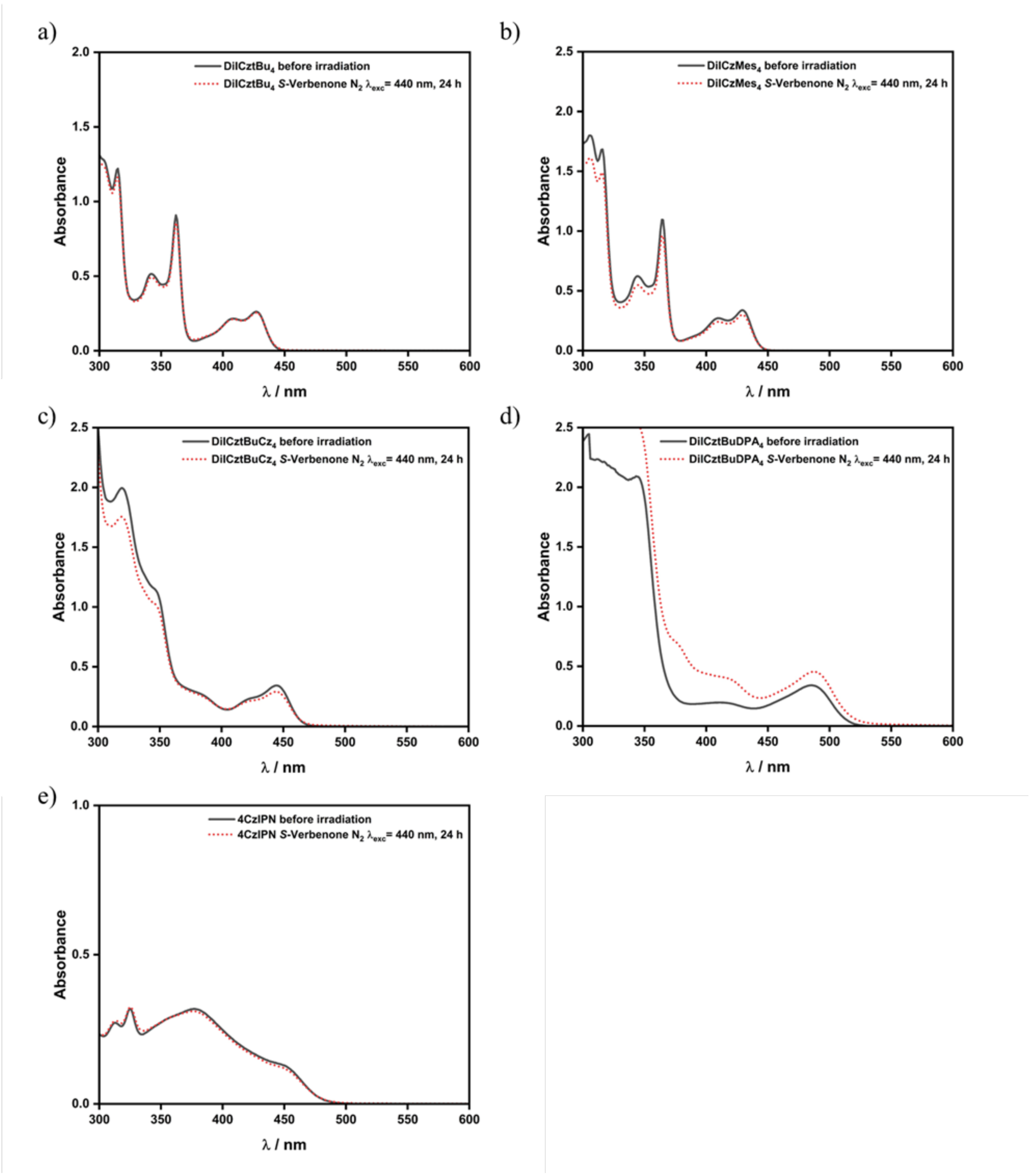

Figure **S53**. Absorbance spectra of the reaction mixture of the simgatropic shift of (*S*)-verbenone photocatalyzed by a) **DiICztBu$_4$**, b) **DiICzMes$_4$**, c) **DiICztBuCz$_4$**, d) **DiICztBuDPA$_4$**, and e) **4CzIPN**. Absorbance spectra compared before and after 24 h irradiation at λ_{exc} = 440 nm.

Esterification via Ni-dual catalysis

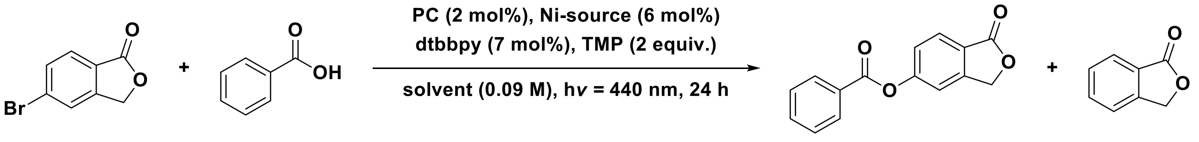

1 2

To an oven-dried vial were added the PC (3.76 µmol, 2 mol%), 5-bromophthalide (40.1 mg, 188 µmol, 1.0 equiv.), benzoic acid (36.7 mg, 301 µmol, 1.6 equiv.), Ni source (11.3 µmol, 6 mol%), 4,4'-di-*tert*-butyl-2,2'-bipyridine (3.53 mg, 13.2 µmol, 7 mol%) followed by three vacuum/nitrogen cycles. Dry solvent (2.00 mL, 91.1 M) and then 2,2,6,6-tetramethylpiperidine (53.1 mg, 376 µmol, 2.0 equiv.) were added, and the mixture bubbled with N_2 for 5 mins. The reaction mixture was stirred and irradiated with λ_{exc} = 440 nm for 24 h. Afterwards, the reactions were filtered over a silica pad and then the yield was determined by ^{1}H NMR spectroscopy using 1,3,5-trimethoxybenzene as the internal standard.

Table **S11**. Yields of the esterification of 5-bromophthalide with benzoic acid.[a]

PC	Ni-source	Solvent	Yield **1** / %	Yield **2** / %
**No PC**	Ni(COD)$_2$	DCM	10 ± 1	11 ± 2
**No PC**	NiBr$_2$•glyme	DCM	0 ± 0	0 ± 0
**DiICztBu$_4$**	Ni(COD)$_2$	DCM	59 ± 2	28 ± 0
**DiICztBu$_4$**	NiBr$_2$•glyme	DCM	31 ± 4	15 ± 1
**DiICzMes$_4$**	Ni(COD)$_2$	DCM	65 ± 3	28 ± 1
**DiICzMes$_4$**	Ni(COD)$_2$	Toluene	10 ± 3	trace
**DiICzMes$_4$**	Ni(COD)$_2$	DMSO	64 ± 5	21 ± 1
**DiICzMes$_4$**	Ni(COD)$_2$	DMSO:toluene 1:1	74 ± 2	15 ± 1
**DiICzMes$_4$**	Ni(COD)$_2$, **3h**	DMSO:toluene 1:1	54 ± 0	13 ± 0
**DiICzMes$_4$**	NiBr$_2$•glyme	DCM	41 ± 0	19 ± 1
**DiICzMes$_4$**	[Ni(dtbbpy)(H$_2$O)$_4$]Cl$_2$	DCM	68 ± 1	18 ± 0
**DiICzMes$_4$**	[Ni(dtbbpy)(H$_2$O)$_4$]Cl$_2$	DMSO:toluene 1:1	81 ± 1	13 ± 1
**DiICztBuCz$_4$**	Ni(COD)$_2$	DCM	60 ± 6	31 ± 2
**DiICztBuCz$_4$**	NiBr$_2$•glyme	DCM	20 ± 1	20 ± 1
**DiICztBuDPA$_4$**	Ni(COD)$_2$	DCM	47 ± 5	14 ± 2
**DiICztBuDPA$_4$**	NiBr$_2$•glyme	DCM	27 ± 3	11 ± 0
**4CzIPN**	Ni(COD)$_2$	DCM	43 ± 2	36 ± 4
**4CzIPN**	NiBr$_2$•glyme	DCM	31 ± 0	19 ± 0
***fac*-Ir(ppy)$_3$**	Ni(COD)$_2$	DCM	65 ± 1	15 ± 1
***fac*-Ir(ppy)$_3$**	NiBr$_2$•glyme	DCM	49 ± 5	16 ± 1
***fac*-Ir(ppy)$_3$**	Ni(COD)$_2$	DMSO:toluene 1:1	77 ± 1	15 ± 0
***fac*-Ir(ppy)$_3$**	Ni(COD)$_2$, **3h**	DMSO:toluene 1:1	68 ± 2	16 ± 2
**SACR-IPTZ**	Ni(COD)$_2$	DMSO:toluene 1:1	99	-

[a]Yields were determined by ^{1}H NMR spectroscopy using 1,3,5-trimethoxybenzene as the internal standard. [b]Yield taken from Ref. [10].

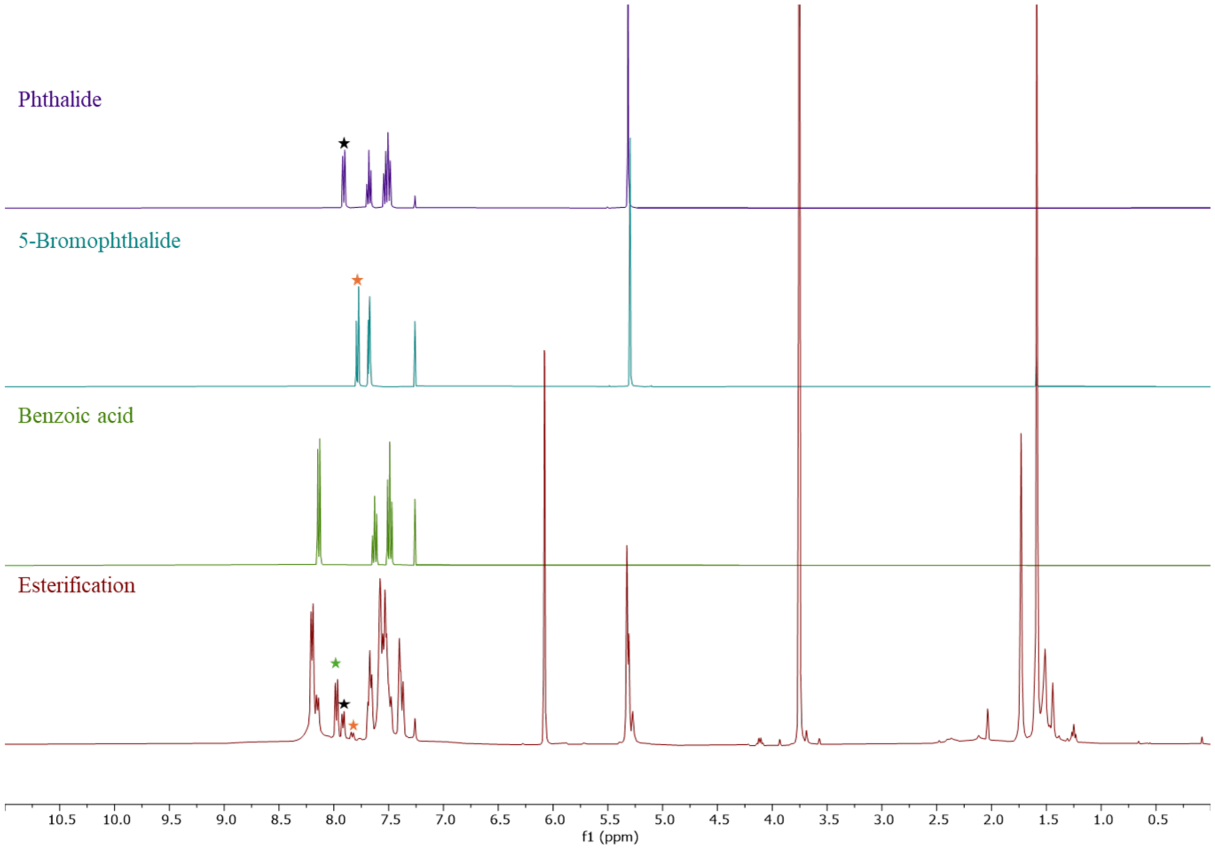

Figure **S54**. Example of the crude NMR spectral analysis of the esterification of 5-bromophthalide with benzoic acid. PC (2 mol%), 5-bromophthalide (188 µmol), benzoic acid (1.6 equiv.), $Ni(COD)_2$ (6 mol%), 4,4'-di-*tert*-butyl-2,2'-bipyridine (7 mol%) and 2,2,6,6-tetramethylpiperidine (2.0 equiv.) in DCM (2.00 mL) The reaction was irradiated (λ_{exc} = 440 nm) for 24 h. The green star marks the product resonances and the orange star marks the remaining starting material resonances. The black star marks the resonances associated with the protodehalogenated product, phthalide.

References

1. M. K. Etherington, N. A. Kukhta, H. F. Higginbotham, A. Danos, A. N. Bismillah, D. R. Graves, P. R. McGonigal, N. Haase, A. Morherr, A. S. Batsanov, C. Pflumm, V. Bhalla, M. R. Bryce and A. P. Monkman, Persistent Dimer Emission in Thermally Activated Delayed Fluorescence Materials, *J. Phys. Chem. C*, 2019, **123**, 11109-11117.
2. M.-j. Bu, C. Cai, F. Gallou and B. H. Lipshutz, PQS-enabled visible-light iridium photoredox catalysis in water at room temperature, *Green Chem.*, 2018, **20**, 1233-1237.
3. G. A. Crosby and J. N. Demas, Measurement of photoluminescence quantum yields. Review, *J. Phys. Chem.*, 1971, **75**, 991-1024.
4. W. H. Melhuish, Quantum efficiencies of fluorescence of organic substances: effect of solvent and concentration of the fluorescent solute1, *J. Phys. Chem.*, 1961, **65**, 229-235.
5. J. Luo and J. Zhang, Donor–Acceptor Fluorophores for Visible-Light-Promoted Organic Synthesis: Photoredox/Ni Dual Catalytic C(sp3)–C(sp2) Cross-Coupling, *ACS Catal.*, 2016, **6**, 873-877.
6. M. Garreau, F. Le Vaillant and J. Waser, C-Terminal Bioconjugation of Peptides through Photoredox Catalyzed Decarboxylative Alkynylation, *Angew. Chem. Int. Ed*, 2019, **58**, 8182-8186.

7. J. Lu, B. Pattengale, Q. Liu, S. Yang, W. Shi, S. Li, J. Huang and J. Zhang, Donor-Acceptor Fluorophores for Energy-Transfer-Mediated Photocatalysis, *J. Am. Chem. Soc.*, 2018, **140**, 13719-13725.
8. M. A. Bryden, F. Millward, T. Matulaitis, D. Chen, M. Villa, A. Fermi, S. Cetin, P. Ceroni and E. Zysman-Colman, Moving Beyond Cyanoarene Thermally Activated Delayed Fluorescence Compounds as Photocatalysts: An Assessment of the Performance of a Pyrimidyl Sulfone Photocatalyst in Comparison to 4CzIPN, *J. Org. Chem.*, 2023, **88**, 6364-6373.
9. S. Sharma and S. Sengupta, Twisted organic TADF triads based on a diindolocarbazole donor for efficient photoisomerization of stilbene and photo-arylation of heteroarenes, *Org. Chem. Front.*, 2023, **10**, 6087-6095.
10. R. Hojo, K. Bergmann, S. A. Elgadi, D. M. Mayder, M. A. Emmanuel, M. S. Oderinde and Z. M. Hudson, Imidazophenothiazine-Based Thermally Activated Delayed Fluorescence Materials with Ultra-Long-Lived Excited States for Energy Transfer Photocatalysis, *J. Am. Chem. Soc.*, 2023, **145**, 18366-18381.
11. S.-J. Woo, Y.-H. Ha, Y.-H. Kim and J.-J. Kim, Effect of ortho-biphenyl substitution on the excited state dynamics of a multi-carbazole TADF molecule, *J. Mater. Chem. C*, 2020, **8**, 12075-12084.
12. E. Duda, D. Hall, S. Bagnich, C. L. Carpenter-Warren, R. Saxena, M. Y. Wong, D. B. Cordes, A. M. Z. Slawin, D. Beljonne, Y. Olivier, E. Zysman-Colman and A. Köhler, Enhancing Thermally Activated Delayed Fluorescence by Fine-Tuning the Dendron Donor Strength, *J. Phys. Chem. B*, 2022, **126**, 552-562.
13. N. D. McClenaghan, R. Passalacqua, F. Loiseau, S. Campagna, B. Verheyde, A. Hameurlaine and W. Dehaen, Ruthenium(II) Dendrimers Containing Carbazole-Based Chromophores as Branches, *J. Am. Chem. Soc.*, 2003, **125**, 5356-5365.
14. V. V. Patil, H. L. Lee, I. Kim, K. H. Lee, W. J. Chung, J. Kim, S. Park, H. Choi, W.-J. Son, S. O. Jeon and J. Y. Lee, Purely Spin-Vibronic Coupling Assisted Triplet to Singlet Up-Conversion for Real Deep Blue Organic Light-Emitting Diodes with Over 20% Efficiency and y Color Coordinate of 0.05, *Adv. Sci.*, 2021, **8**, 2101137.
15. T. Taniguchi, Y. Itai, Y. Nishii, N. Tohnai and M. Miura, Construction of Nitrogen-containing Polycyclic Aromatic Compounds by Intramolecular Oxidative C-H/C-H Coupling of Bis(9H-carbazol-9-yl)benzenes and Their Properties, *Chem. Lett.*, 2019, **48**, 1160-1163.
16. D. Hall, K. Stavrou, E. Duda, A. Danos, S. Bagnich, S. Warriner, A. M. Z. Slawin, D. Beljonne, A. Köhler, A. Monkman, Y. Olivier and E. Zysman-Colman, Diindolocarbazole – achieving multiresonant thermally activated delayed fluorescence without the need for acceptor units, *Mater. Horiz.*, 2022, **9**, 1068-1080.
17. T. Constantin, M. Zanini, A. Regni, N. S. Sheikh, F. Juliá and D. Leonori, Aminoalkyl radicals as halogen-atom transfer agents for activation of alkyl and aryl halides, *Science*, 2020, **367**, 1021-1026.
18. L. Schmid, F. Glaser, R. Schaer and O. S. Wenger, High Triplet Energy Iridium(III) Isocyanoborato Complex for Photochemical Upconversion, Photoredox and Energy Transfer Catalysis, *J. Am. Chem. Soc.*, 2022, **144**, 963-976.
